# Supplementary material for: Deciphering intercellular signaling complexes by interaction-guided chemical proteomics
Source: Nat Commun. 2023 Jul 12;14:4138. doi: 10.1038/s41467-023-39881-9 (PMC10338493; doi:10.1038/s41467-023-39881-9)
Supplement: Supplementary file 1 — Supplementary Information [file 41467_2023_39881_MOESM1_ESM.pdf]

Supplementary Information for

Deciphering intercellular signaling complexes by interaction-guided chemical proteomics

Jiangnan Zheng[1,4,*], Zhendong Zheng[1,2,4], Changying Fu[1,4], Yicheng Weng[1], An He[1], Xueting Ye[1], Weina Gao[1] and Ruijun Tian[1,3,*]

[1] Department of Chemistry, School of Science, Southern University of Science and Technology, Shenzhen 518055, China;

[2] School of Environment, Harbin Institute of Technology, Harbin 150090, China;

[3] Research Center for Chemical Biology and Omics Analysis, School of Science, Southern University of Science and Technology, 1088 Xueyuan Road, Shenzhen 518055, China;

[4] These authors contributed equally: Jiangnan Zheng, Zhendong Zheng, Changying Fu;

* E-mail: tianrj@sustech.edu.cn, zhengjn@sustech.edu.cn

Table of Contents

Supplementary Note 1: Chemical synthesis and characterization 4

Synthesis of probe 1 and probe 2 4

Synthesis of probe 3 10

Supplementary Note 2: LC-MS/MS analysis 13

Supplementary Note 3: Recombinant PLAU expression and purification 15

Supplementary Figures 16

MS Quality Control 31

Quality control of the MS data in Fig. 2b 31

Quality control of the MS data in Fig. 2c 33

Quality control of the MS data in Fig. 2d 35

Quality control of the MS data in Fig. 2e 37

Quality control of the MS data in Fig. 3b 39

Quality control of the MS data in Fig. 3d 40

Quality control of the MS data in Fig. 3f 41

Quality control of the MS data in Fig. 3e 43

Quality control of the MS data in Fig. 4b 45

Quality control of the MS data in Fig. 4c 46

Quality control of the MS data in Fig. 4d 47

Quality control of the MS data in Fig. 6d 50

Quality control of the MS data in Fig. 6e 53

Quality control of the MS data in Supplementary Fig. 7 55

Quality control of the MS data in Supplementary Fig. 8a 56

Quality control of the MS data in Supplementary Fig. 8b 58

Quality control of the MS data in Supplementary Fig. 8c 60

Quality control of the MS data in Supplementary Fig. 8d 63

Quality control of the MS data in Supplementary Fig. 8e 65

Quality control of the MS data in Supplementary Fig. 8f 67

Quality control of the MS data in Supplementary Fig. 8g 69

Quality control of the MS data in Supplementary Fig. 8h 71

Quality control of the MS data in Supplementary Fig. 8i 72
Quality control of the MS data in Supplementary Fig. 8j, k 73
Quality control of the MS data in Supplementary Fig. 9 74
Quality control of the MS data in Supplementary Fig. 10a 76
Quality control of the MS data in Supplementary Fig. 10b 79
Quality control of the MS data in Supplementary Fig. 12a 82
Quality control of the MS data in Supplementary Fig. 12b 85
Quality control of the MS data in Supplementary Fig. 12c 88
Quality control of the MS data in Supplementary Fig. 12d 91
Quality control of the MS data in Supplementary Fig. 13a 93
Quality control of the MS data in Supplementary Fig. 13b 96
Quality control of the MS data in Supplementary Fig. 13c 99
Quality control of the MS data in Supplementary Fig. 13d 102
Quality control of the MS data in Supplementary Fig. 14a 105
Quality control of the MS data in Supplementary Fig. 14b 105
Quality control of the MS data in Supplementary Fig. 14c 106
Quality control of the MS data in Supplementary Fig. 14d 107
Quality control of the MS data in Supplementary Fig. 14e 107
Quality control of the MS data in Supplementary Fig. 14f 108
Supplementary Tables 109
References 110

Supplementary Note 1: Chemical synthesis and characterization

Synthesis of probe 1 and probe 2

The synthesis route of probe 1[1] and probe 2 is shown in **Supplementary Fig. 1**.

To a solution of 6-(6-Boc-aminohexanamido)hexanoic acid (3.9 g, 11.3 mmol) in 40 mL dichloromethane, hydroxysuccinimide (2.6 g, 22.6 mmol) was added at room temperature (RT). Then EDCI (6.47 g, 33.9 mmol) was added in several portions. MS result indicated the starting material disappeared after overnight stirring. The mixture was diluted with an additional 40 mL of dichloromethane and washed with 60 mL of water three times. The organic layers were dried over Na_2SO_4 and evaporated to dryness. The crude product was chromatographed on silica gel (acetyl acetate) to yield compound c1 (3.5 g, yield 70.0%).

To a solution of Fmoc-Lys (Boc)-OH (1.39 g, 2.97 mmol) in DMF, hexafluorophosphate benzotriazole tetramethyl uronium (HBTU; 1.35 g, 3.56 mmol) and DIPEA (0.46 g, 3.56 mmol) were added and stirred for 20 minutes. Then Biotin-PEG3-$(CH_2)_3$-NH_2 in DMF was added to the mixture and incubated for 3 h. TLC analysis confirmed the disappearance of the starting material Fmoc-Lys (Boc)-OH. The mixture was then evaporated to dryness under vacuum. The crude product was subjected to chromatography using silica gel, resulting in the isolation of c2 (1.62 g, 61.1% yield).

To a solution of c2 (3.27 g, 3.65 mmol) in DCM, TFA was added at an ice bath. After stirring for 3 h, TLC analysis indicated the disappearance of starting material c3. The mixture was evaporated to dryness under vacuum. After repeated solvent evaporation (20 mL MeOH, three times), the remaining mixture was dried under an oil pump. The mixture was suspended in MeOH, and TEA was added to the mixture to adjust the pH>8. Then c1 (2.25 g, 5.11 mmol) was added, and the mixture was incubated at RT for 2h. TLC analysis indicated the disappearance of c2-NH_2. The mixture was evaporated to dryness and subjected to chromatography using silica gel to get c3 (1.585 g, 38.7% yield).

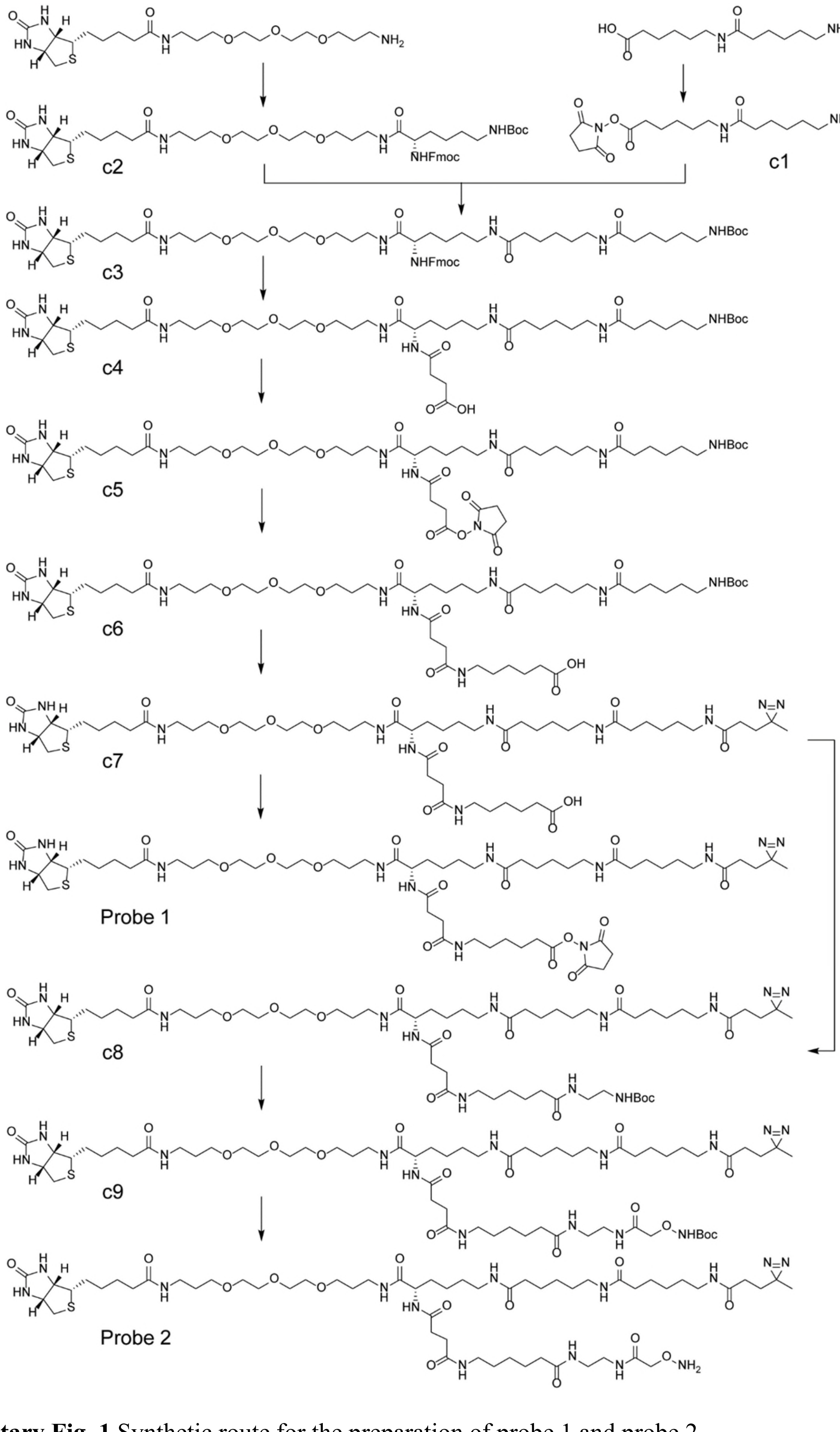

Supplementary Fig. 1 Synthetic route for the preparation of probe 1 and probe 2.

To a solution of c3 (2 g, 1.78 mmol) in DCM, N,N-diethylamine (2.6 g, 35.6 mmol) was added at RT. After stirring for 5 hours, TLC analysis indicated the disappearance of the starting material c4. The mixture was evaporated to dryness under vacuum. After repeated solvent evaporation (20 mL ACN, three times), the remaining mixture was dried under an oil pump. The dried mixture was suspended in a mixture of DMF and DCM in a 1:1 ratio, and succinic anhydride (214 mg, 2.14 mmol) and DIPEA were added to the mixture. The mixture was incubated at RT overnight. TLC analysis indicated the disappearance of c3-NH_2. The mixture was evaporated to dryness and subjected to chromatography using silica gel to get c4 (1 g, 56.18% yield).To a solution of c4, hydroxysuccinimide in DMF, EDCI was added at RT. After stirring for 12 hours, TLC analysis indicated the disappearance of the starting material c4. The mixture was evaporated to dryness and subjected to chromatography using silica gel (using a ACN:DMF solvent mixture in a ratio of 15:1 to 10:1). The solvents containing c5 were evaporated to remove ACN. The residue was then added to 6-aminocaproic acid. After stirring for 12 hours, TLC analysis indicated the disappearance of the starting material c5. The mixture was dried using an oil pump and subjected to chromatography using silica gel, resulting in the isolation of c6 (100 mg, 17.2% yield).

TFA (1.026 g, 9 mmol) was added to a solution of c6 (100 mg, 0.09 mmol) in dichloromethane (5 mL) at 0 °C. After stirring for 2 hours, TLC analysis indicated the disappearance of the starting material c6. The mixture was evaporated under vacuum. The residue was then dissolved in DMF (5 mL), and triethylamine was added to adjust the pH to greater than 8. Then, 2,5-dioxopyrrolidin-1-yl 3-(3-methyl-3H-diazirin-3-yl)propanoate (30.4 mg, 0.135 mmol) was added, and the mixture was incubated overnight. The solvent was removed under vacuum, and the resulting residue was subjected to chromatography using silica gel to get c7 (61 mg, 60.5% yield).

EDCI (30 mg, 0.158 mmol) was added to a solution of compound c7 (60 mg, 0.053 mmol) and

hydroxysuccinimide (12 mg, 0.105 mmol) in DMF (5 mL). The mixture was incubated overnight. When the compound c7 disappeared (monitored by MS results), the solvent was removed under vacuum. The residue was then separated and purified using semi-preparative high-performance liquid chromatography, resulting in the isolation of compound probe 1 (16 mg, 24.5% yield).

A mixture of c7 (302 mg, 0.269 mmol), HBTU (153 mg, 0.403 mmol), hydroxybenzotriazole (HOBT, 55 mg, 0.403 mmol) and *N,N*-Diisopropylethylamine (DIPEA, 139 mg, 0.403 mmol) was dissolved in dimethylformamide (DMF, 10 mL) and stirred at RT for 50 min. The *N*-Boc-ethylenediamine (65 mg, 0.403 mmol) in 5 mL DMF was added dropwise to the mixture. The reaction was incubated overnight. Then, DMF was removed, and the residue was separated by silica gel to obtained c8 in 82.5% yield (281 mg).

Compound c8 (140 mg, 0.111 mmol) was dissolved in dichloromethane (DCM) and cooled to 0 °C. Then, TFA (3.8 g, 33.3 mmol) was added and stirred at 0 °C for 2 h. After the Boc protecting group was removed from c7, the DCM was removed and the sample was re-dissolved in DMF (5 mL). Then, the pH was adjusted to greater than 7 using DIPEA.

A mixture of Boc-3-(aminooxy)acetic acid (32 mg, 0.167 mmol), HBTU (95 mg, 0.25 mmol), HOBT (31 mg, 0.25 mmol) and DIPEA (86 mg, 0.666 mmol) was dissolved in DMF (10 mL) and stirred at RT for 50 minutes. The deprotection c8 was added dropwise to the mixture and incubated overnight. Then, DMF was removed, and the residue was separated by silica gel to obtained c9 in 81.2% yield (120 mg).

The c9 (120 mg, 0.09 mmol) was dissolved in DCM and cooled to 0 °C. Then TFA (3.1 g, 27 mmol) was added and stirred at 0 °C for 2 h to remove Boc protecting group. After removal of DCM and TFA, the product was re-dissolved in ACN/H_2O (v/v=1:1, 5 mL). Finally, the mixture was separated and purified via semi-preparative high-performance liquid chromatography (HPLC, Agilent) to obtain probe 2 with a yield of 15.9% (17.7 mg). Mass spectrometry analysis was performed on a Q-Exactive Orbitrap mass spectrometer (Thermo

Fisher). NMR spectra were acquired using a 400 MHz machine (Bruker). **^{1}H NMR** (400 MHz, DMSO-d6) δ 8.22 (q, J = 5.6 Hz, 3H), 7.98 (d, J = 8.0 Hz, 1H), 7.86 (q, J = 5.6 Hz, 2H), 7.74 (d, J = 36.2 Hz, 4H), 4.36 (d, J = 57.8 Hz, 3H), 4.09 (d, J = 37.2 Hz, 2H), 3.82 (dd, J = 5.4, 2.4 Hz, 2H), 3.47 (d, J = 29.0 Hz, 11H), 3.36 (d, J = 5.6 Hz, 2H), 3.04 (d, J = 51.9 Hz, 13H), 2.81 (dd, J = 12.5, 5.0 Hz, 2H), 2.57 (d, J = 12.4 Hz, 1H), 2.31 (d, J = 34.8 Hz, 4H), 2.03 (d, J = 30.3 Hz, 8H), 1.19 (s, 36H). **^{13}C NMR** (101 MHz, DMSO) δ 172.41, 172.29, 171.85, 167.06, 163.22, 159.39, 159.01, 158.64, 158.26, 120.02, 117.14, 114.26, 111.39, 81.79, 73.22, 71.62, 70.21, 70.19, 70.06, 69.99, 69.91, 68.54, 68.47, 67.77, 61.52, 59.67, 55.89, 53.10, 38.86, 38.71, 36.32, 36.17, 35.81, 35.75, 35.65, 35.44, 31.14, 29.85, 29.66, 29.43, 29.33, 29.13, 28.68, 28.49, 28.16, 26.61, 26.53, 25.77, 25.52, 25.45, 25.33, 23.42. **HRMS** (*m/z*): $[M+H]^+$ calcd for $C_{57}H_{103}N_{14}O_{14}S$ 1239.7499, found 1239.7514.

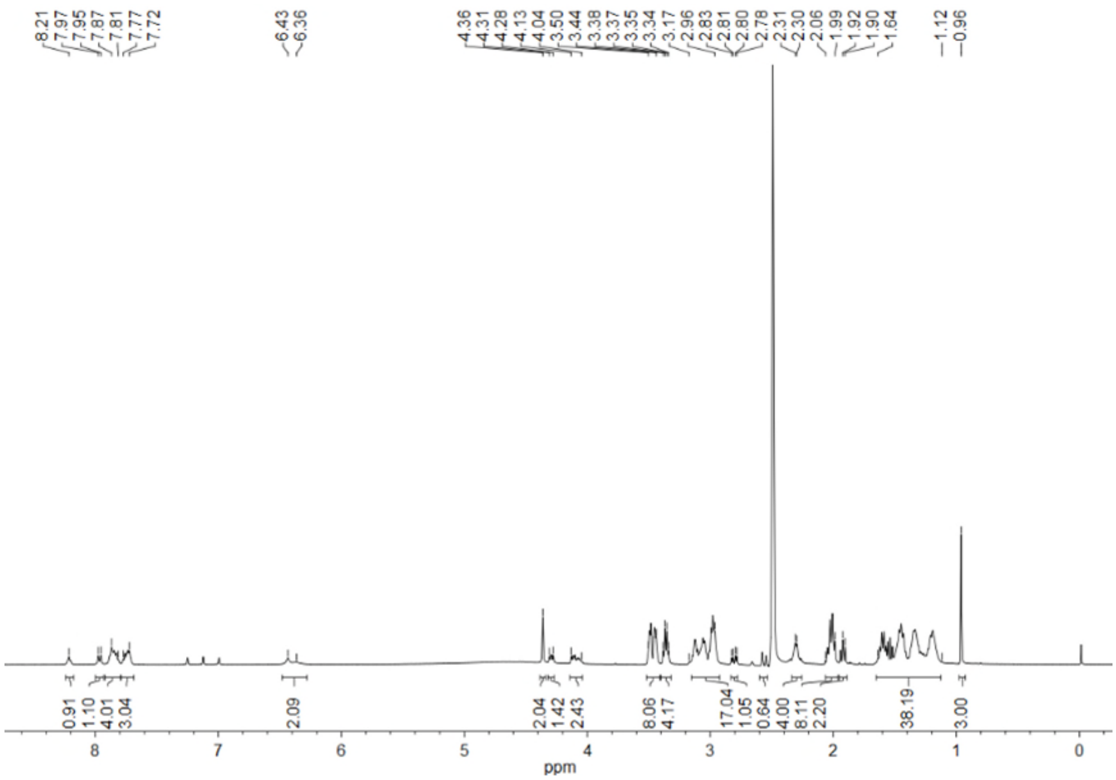

Supplementary Fig. 2 ^{1}H NMR spectrum of probe 2.

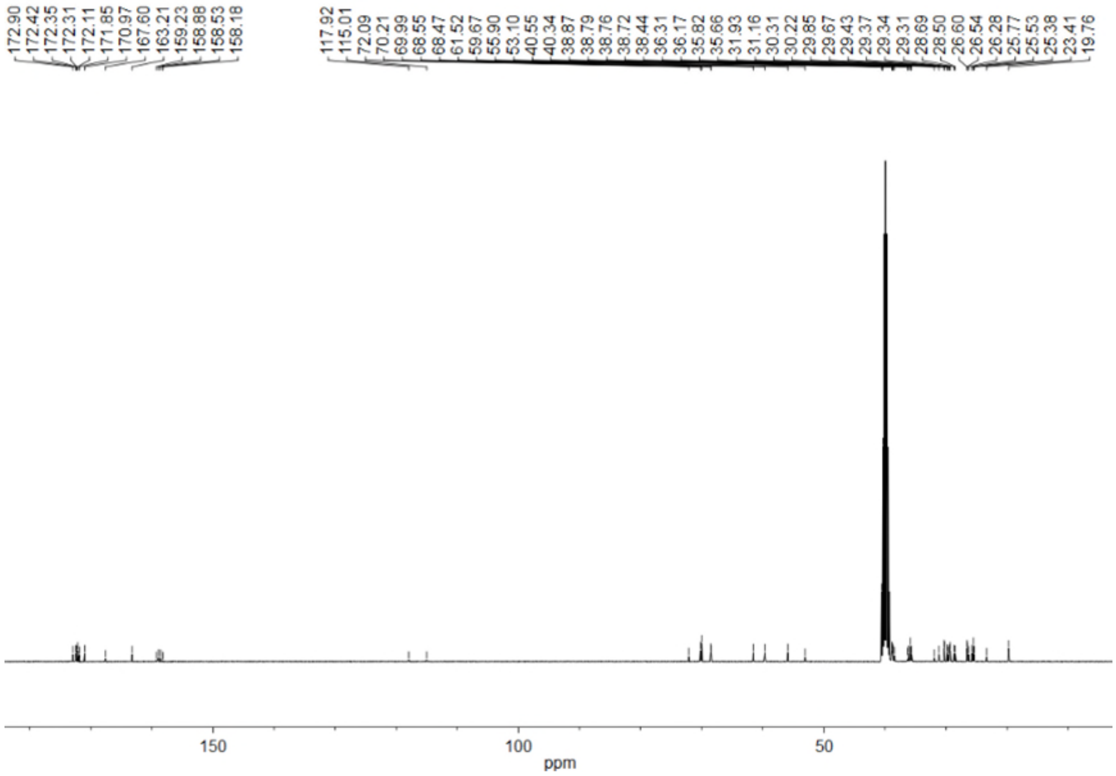

Supplementary Fig. 3 ^{13}C NMR spectrum of probe 2.

Synthesis of probe 3

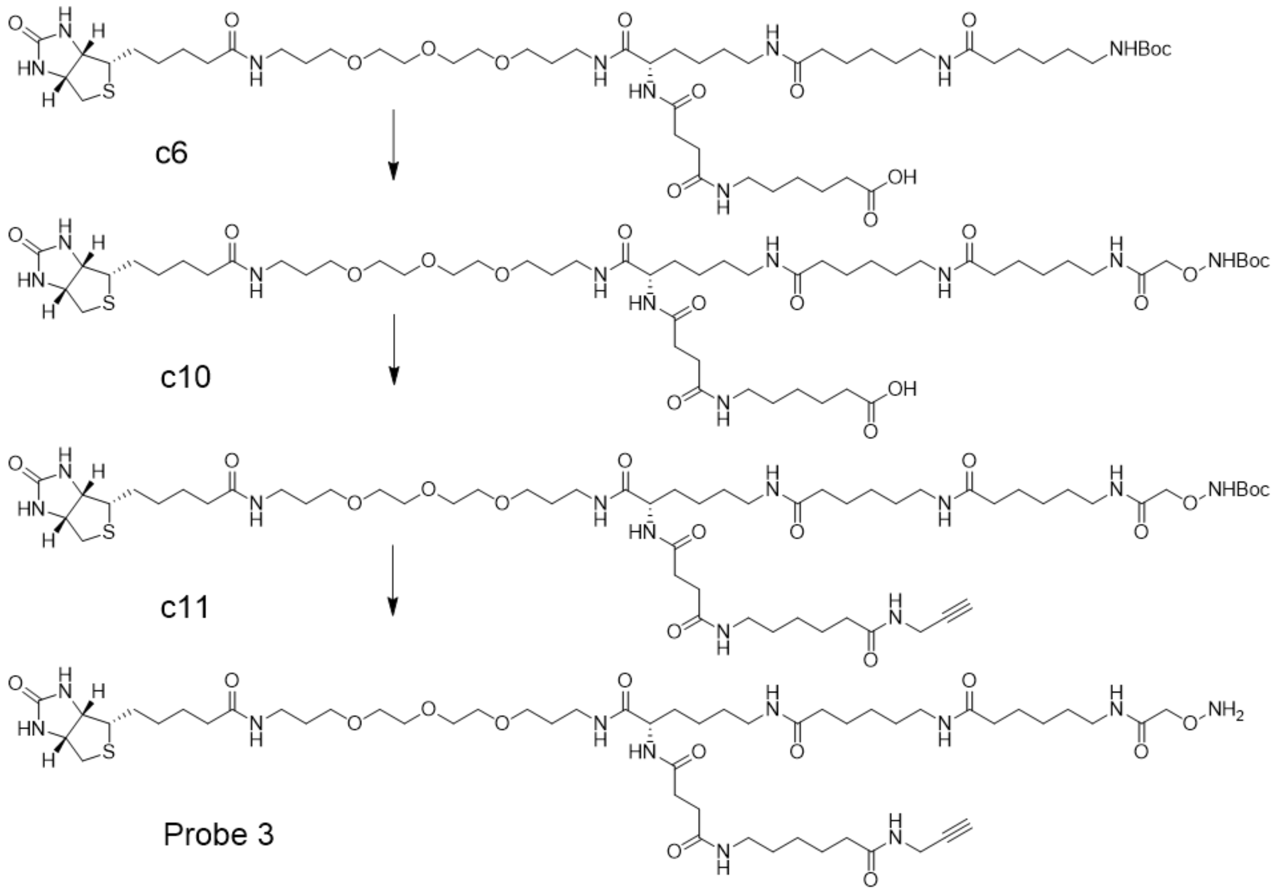

Supplementary Fig. 4 Roadmap for probe 3 synthesis.

A mixture of c6 (200 mg, 0.179 mmol), HBTU (102 mg, 0.268 mmol), HOBT (36 mg, 0.268 mmol) and DIPEA (34.5 mg, 0.268 mmol) was dissolved in DMF (10 mL) and stirred at RT for 50 min. The mono-propargylamine (15 mg, 0.268 mmol) was dissolved in 2 mL DMF and added dropwise to the mixture. The reaction was incubated overnight. Then, DMF was removed, and the residue was separated by silica gel to obtained c10 in 72.6% yield (150 mg).

Compound c10 (150 mg, 0.13 mmol) was dissolved in DCM and cooled to 0 °C. Then, TFA (1.482 g, 13 mmol) was added and stirred at 0 °C for 2 h. The DCM was removed and the sample was re-dissolved in DMF (5 mL). Then, the pH was adjusted to greater than 7 using DIPEA. A mixture of Boc-3-(aminooxy)acetic acid (37.3 mg, 0.195 mmol), HBTU (111 mg, 0.293 mmol), HOBT (39.6 mg, 0.293 mmol) and DIPEA (37.8 mg, 0.293 mmol) was dissolved in DMF (10 mL) and stirred at RT for 50 min. The deprotected c10 was added

dropwise to the mixture and incubated overnight. Then DMF was removed, and the residue was separated by silica gel to obtained c11 in 86.6% yield (137.7 mg).

Compound c11 (137.7 mg, 0.113 mmol) was dissolved in DCM and cooled to 0 °C. Then, TFA (1.3 g, 11.3 mmol) was added and stirred at 0 °C for 2 h. After removal of DCM and TFA, the product was re-dissolved in ACN/H_2O (v/v=1:1, 5 mL). Finally, the mixture was separated and purified via semi-preparative HPLC to obtain probe 3 with a yield of 40.5% (51.4 mg). **^{1}H NMR** (400 MHz, DMSO-d6) δ 8.21 (s, 1H), 7.96 (d, J = 8.0 Hz, 1H), 7.84 (d, J = 21.9 Hz, 4H), 7.75 (d, J = 19.4 Hz, 3H), 6.40 (d, J = 28.6 Hz, 2H), 4.36 (s, 2H), 4.29 (d, J = 12.5 Hz, 1H), 4.09 (d, J = 33.6 Hz, 2H), 3.47 (d, J = 25.7 Hz, 8H), 3.36 (q, J = 6.1 Hz, 4H), 2.96 (s, 17H), 2.80 (dd, J = 12.4, 5.0 Hz, 1H), 2.56 (d, J = 12.4 Hz, 1H), 2.30 (d, J = 5.1 Hz, 4H), 2.02 (d, J = 30.5 Hz, 8H), 1.91 (d, J = 8.0 Hz, 2H), 1.64 (s, 38H), 0.96 (s, 3H). **^{13}C NMR** (101 MHz, DMSO) δ 172.90, 172.42, 172.35, 172.31, 172.11, 171.85, 170.97, 167.60, 163.21, 159.23, 158.88, 158.53, 158.18, 117.92, 115.01, 72.09, 70.21, 69.99, 68.55, 68.47, 61.52, 59.67, 55.90, 53.10, 40.55, 40.34, 38.87, 38.79, 38.76, 38.72, 38.44, 36.31, 36.17, 35.82, 35.66, 31.93, 31.16, 30.31, 30.22, 29.85, 29.67, 29.43, 29.37, 29.34, 29.31, 28.69, 28.50, 26.60, 26.54, 26.28, 25.77, 25.53, 25.38, 23.41, 19.76. **HRMS** (*m/z*): $[M+H]^+$ calcd for $C_{53}H_{94}N_{11}O_{13}S$ 1124.6753, found 1124.6772.

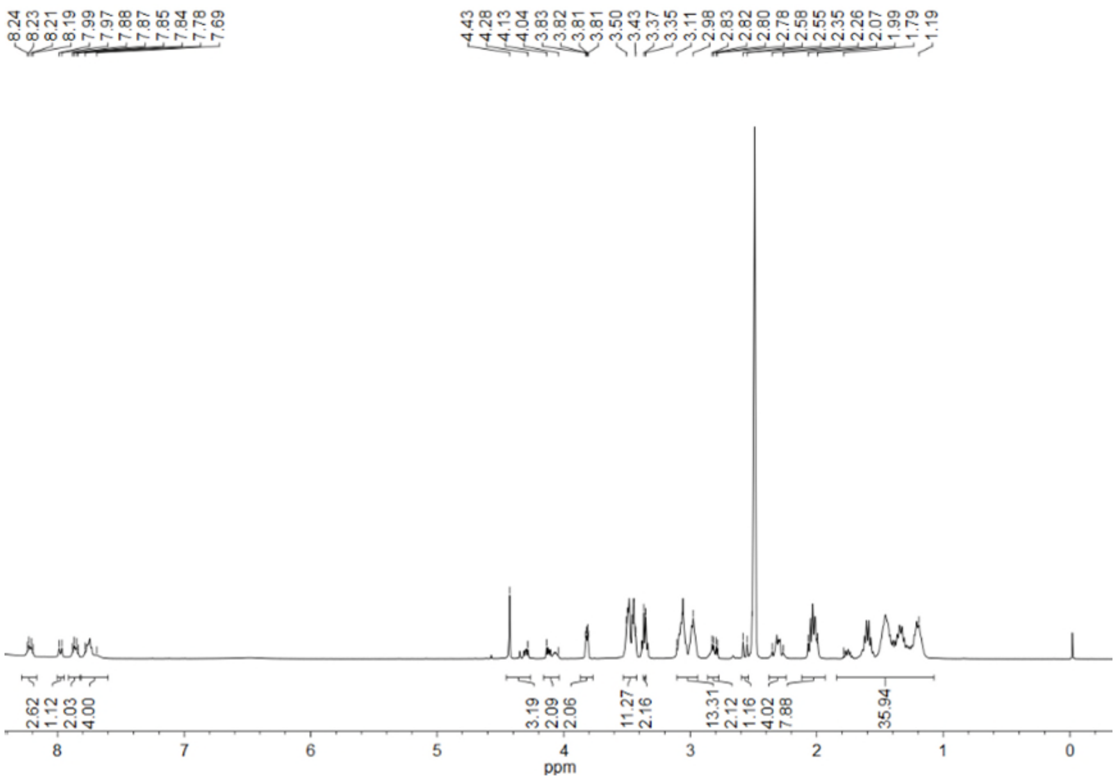

Supplementary Fig. 5 ^{1}H NMR spectrum of probe 3.

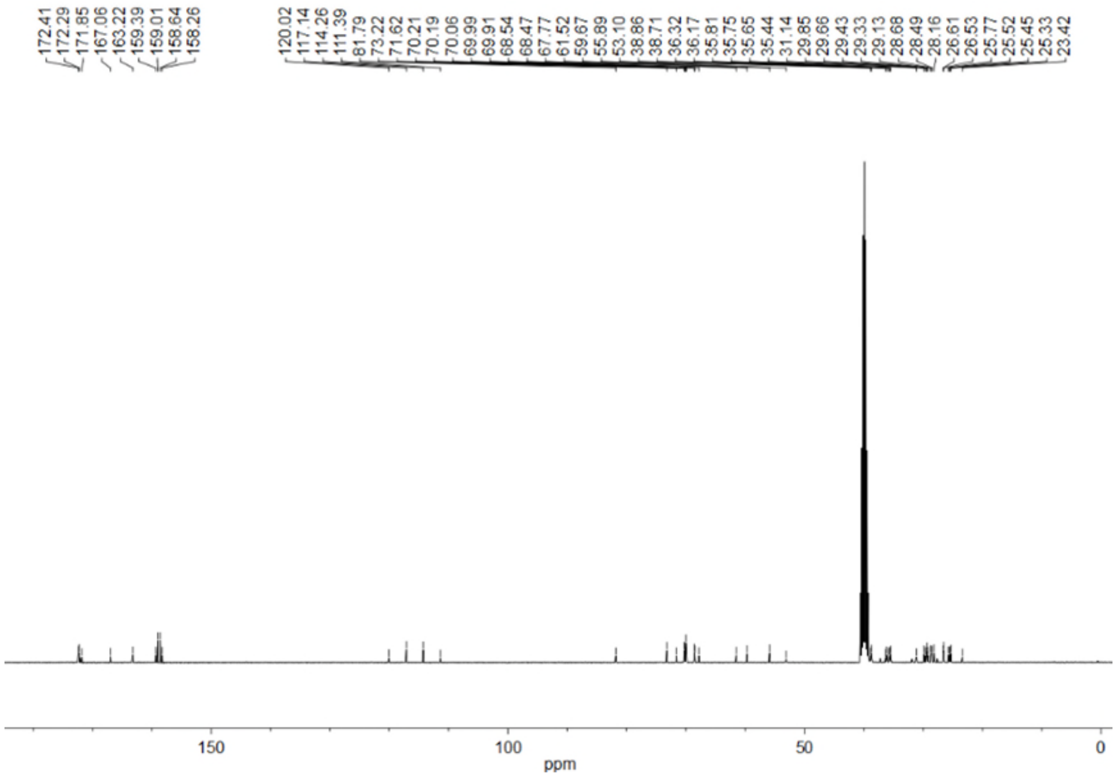

Supplementary Fig. 6 ^{13}C NMR spectrum of probe 3.

Supplementary Note 2: LC-MS/MS analysis

For HeLa surfaceome analysis, samples were analyzed with EASY-nLC 1000 system coupled to an Orbitrap Fusion mass spectrometer. Peptides were separated at a flow rate of 250 nL/min with an effective gradient from 7% to 22% of buffer B over 100 min, 22% to 35% of buffer B over 20 min (Buffer A: 0.1% FA in water; Buffer B: 0.1% FA in ACN). Full MS scan was performed with the range of 350–1,550 m/z, with a mass resolution of 120,000, an AGC target of 2×10^5 and a maximum IT of 100 ms. Precursor ions were selected with Top Speed method using 3 s cycle time, 30 s dynamic exclusion, and fragmentated by HCD with 30% NCE. MS2 spectra were acquired in the linear ion trap with an AGC of 10,000 and a maximum IT of 35 ms.

For IGC experiments on PCC cells, PCC surfaceome analysis and PSC secretome analysis, samples were analyzed using EASY-nLC 1200 system coupled to a Q Exactive HF-X mass spectrometer. Peptides were separated with an effective gradient from 9 to 26% buffer B in 44 min, followed by a 9 min gradient to 40% of B, 2 min to 97% B, 10 min at 97% B at 350 nL/min (Buffer A: 0.1% FA in water; Buffer B: 0.1% FA in 80% ACN). Eluted peptides were analyzed with a Top30 method with full MS scan (m/z 350-1400) at 120,000 resolution with an AGC target of 3×10^6 and a maximum IT of 120 ms. For MS2 analysis, a resolution of 15000, an AGC target of 3×10^5, a maximum IT of 40 ms, an isolation window of 1.3 m/z, HCD with a NCE of 27, dynamically excluded time of 30 s were set. MS data were acquired using Xcalibur 4.1 software.

For IGC experiments on PSC cells, PSC surfaceome analysis and PCC secretome analysis, samples were separated with an in-house packed column (50 μm × 20 cm, ReproSil-Pur C18-AQ, 1.9 μm, 120 Å, Dr. Maisch) and analyzed on a nanoElute UHPLC coupled to a Bruker timsTOF Pro mass spectrometer. LC separation was achieved at a flow of 100 nL/min using a 50 min gradient from 4 to 19% of buffer B, followed by a 10 min gradient to 90% of buffer B (Buffer B: 0.1% FA in ACN). The electrospray ionization (ESI) source settings were as follows: 4500 V capillary voltage, 500 V end plate offset, and 3.0 L/min of dry

gas at temperature of 180 °C. The survey scan was acquired (0.75 to 1.30 V s/cm^2 (1/k0), 350-1500 m/z) in DDA-PASEF (data dependent acquisition Parallel Accumulation Serial Fragmentation) mode[2]. The total cycle time was set to 0.86 s, and the number of PASEF MS/MS scans was set to 4. For PASEF MSMS scanning, the collision energy was ramped linearly as a function of the mobility from 59 eV at 1/k0 = 1.6 Vs/cm^2 to 20 eV at 1/k0 = 0.6 Vs/cm^2. The mass spectrometers control software Bruker otofControl v6.2 was used to acquire the MS data.

Supplementary Note 3: Recombinant PLAU expression and purification

The sequence coding for PLAU protein (GenBank accession number NM_002658.6 [https://www.ncbi.nlm.nih.gov/nuccore/NM_002658.6]) was synthesized and cloned into the mammalian expression vector PCAGGS fused with an N-terminal signal peptide and an S tag. The plasmids PCAGGS/PLAU were transiently transfected into HEK 293T cells using the Lipofectamine 3000 transfection kit (Thermo Fisher Scientific, L3000001). Cells were washed twice with PBS at 6 h post-transfection and then cultured in serum-free FreeStyle 293 Expression Medium (Gibco, #12338-018). The CM was collected at 72 h post-transfection and centrifuged at 4000g for 10 min. After filtration through a 0.22-μm syringe filter (Millipore), the CM was buffer exchanged into PBS in a 3-kDa cut-off Amicon Ultra centrifugal filter (Millipore) and then incubated with S tag agarose beads overnight at 4 °C. The bound proteins were eluted from the beads with 3 M magnesium chloride and buffer exchanged into PBS and stored at -80 °C until further use.

Supplementary Figures

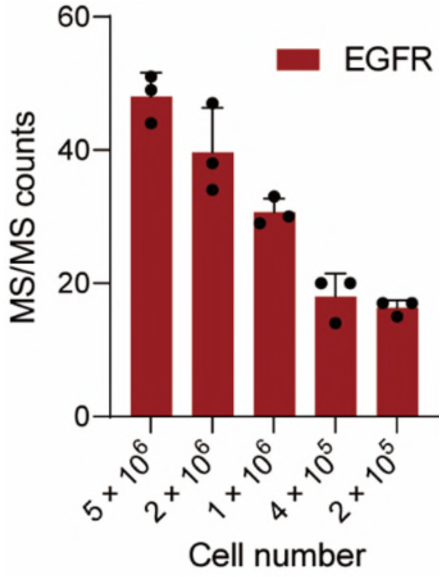

Supplementary Fig. 7 Quantitative comparison of the identified EGFR from different amount of HeLa cells by Photo-IGC using probe 1. Data are presented as mean ± SD (*n* = 3 biological replicates).

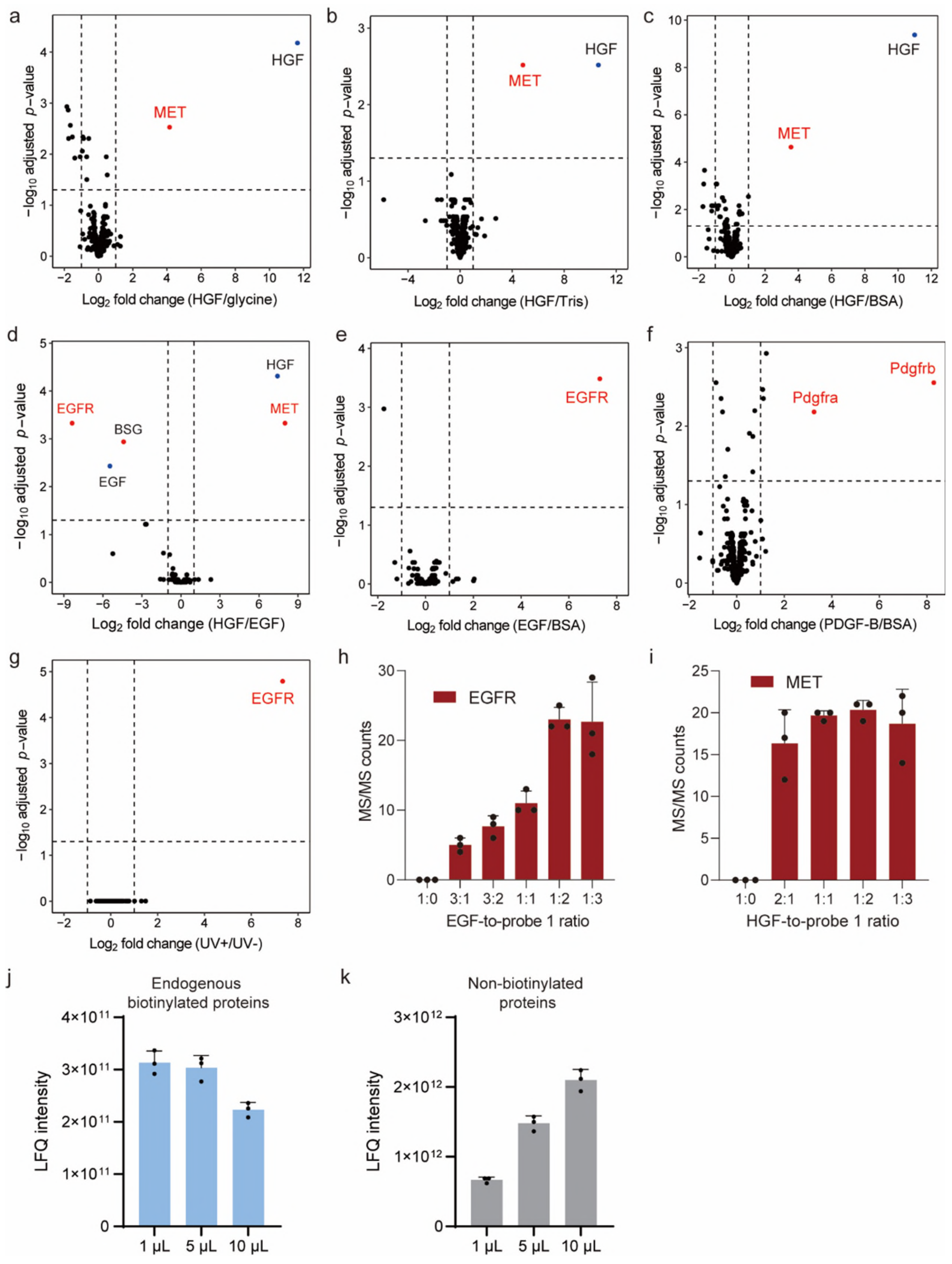

Supplementary Fig. 8 Identification of receptors of HGF (100 ng) from 1 million HeLa cells using the Photo-IGC approach. Equal amounts of (**a**) BSA, (**b**) glycine and (**c**) Tris were used as negative controls, respectively. **d** Photo-IGC experiments on 6 million HeLa cells using EGF (44 ng) and HGF (55 ng) as ligands. **e, f** Photo-

IGC experiments for the identification of receptors of (**e**) EGF (100 ng) from 1 million HeLa cells and (**f**) PDGFB (100 ng) from 1 million NIH 3T3 cells. BSA was used as the negative control. **g** Photo-IGC with 50 ng of EGF on 1 million HeLa cells. The group without UV irradiation was used as a negative control. **h** Identification of EGFR on 1 million HeLa cells by Photo-IGC using 60 ng of EGF conjugated with different mass ratio of probe 1. Data are presented as mean ± SD. **i** Identification of MET on 1 million HeLa cells by Photo-IGC using 30 ng of HGF conjugated with different mass ratio of probe 1. Data are presented as mean ± SD. **j, k** AP-MS analysis of the biotinylated proteins from the lysates of 1 million HeLa cells with different volumes of streptavidin beads (1 μL, 5 μL, and 10 μL). As the endogenous biotinylated proteins were dominated in the IGC experiments, the LFQ intensities of them were used to determine the optimal amount of beads required. The sum of LFQ intensities of the (**j**) endogenous biotinylated proteins (ACACA, PC, PCCA, MCCC1 and ACACB) and (**k**) non-biotinylated proteins were shown. Data are presented as mean ± SD ($n = 3$ biological replicates). All experiments in the figure were performed in triplicate per condition.

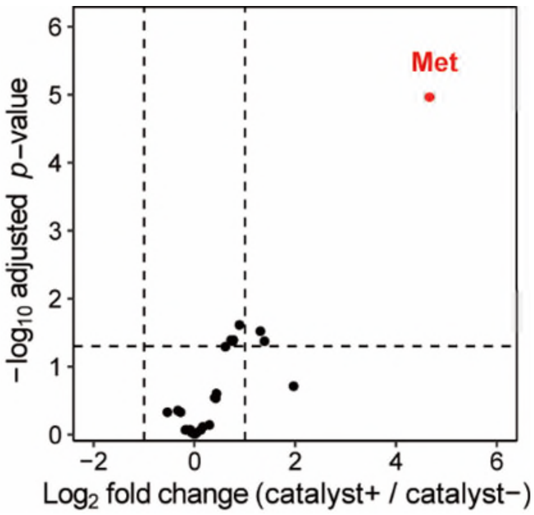

Supplementary Fig. 9 Click-IGC experiments with HGF-BSA mixture (HGF:BSA=1:500, wt/wt) as ligand on 1 million NIH 3T3 cells. All experiments were performed in triplicate per condition.

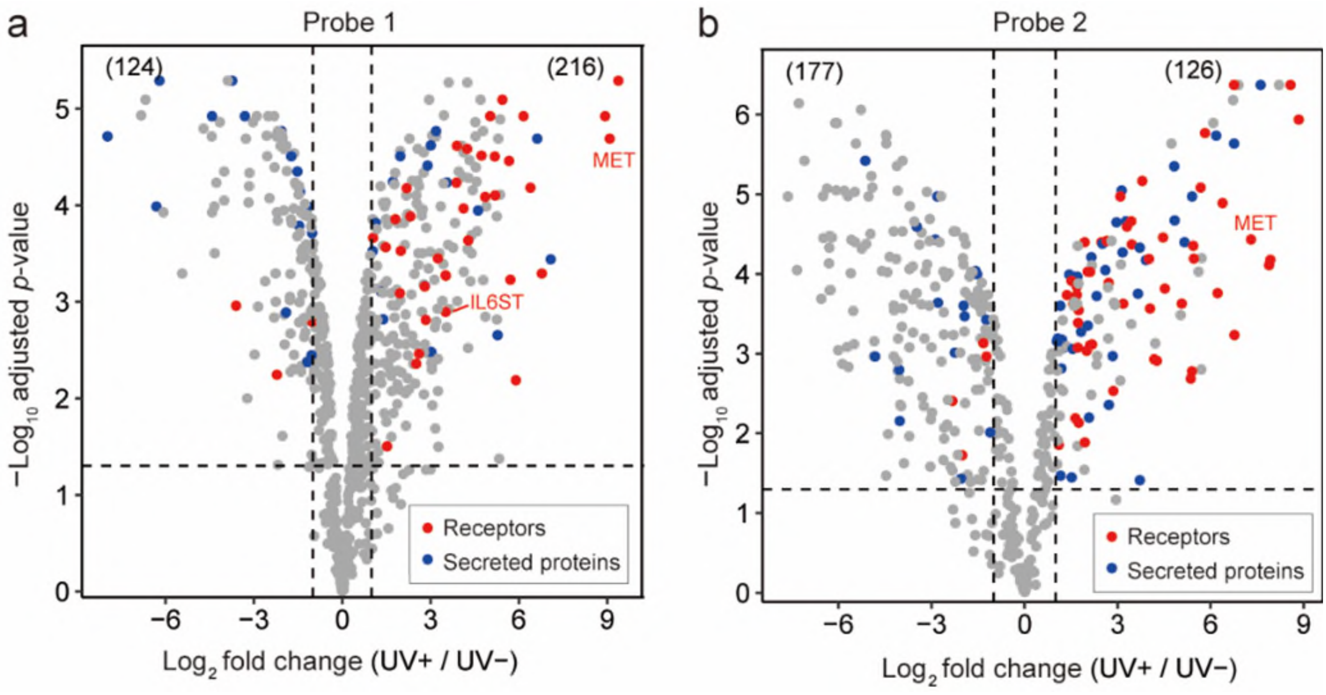

Supplementary Fig. 10 (**a**) Probe 1 and (**b**) probe 2 based Photo-IGC with KP4-cCM as ligands on HeLa cells. The significantly changed UniProt annotated receptors and secreted proteins are highlighted in red and blue, respectively. All experiments were performed in triplicate per condition.

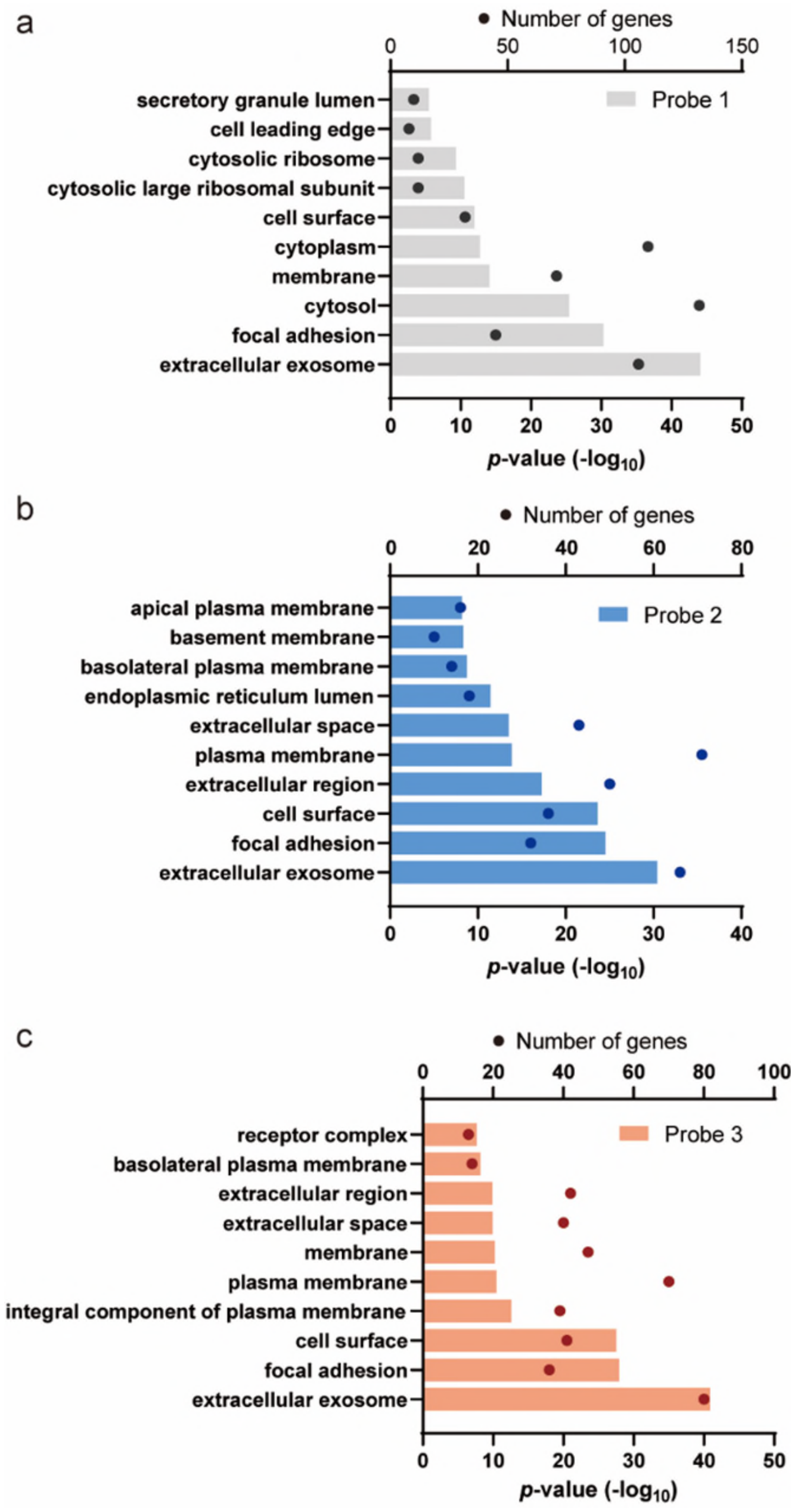

Supplementary Fig. 11 Gene ontology (GO) term enrichment analysis of the significant proteins in volcano plot (Fig. 4d and Supplementary Fig. 10). Top 10 GO cellular component terms for the significant proteins identified by (**a**) probe 1, (**b**) probe 2 and (**c**) probe 3 based IGC.

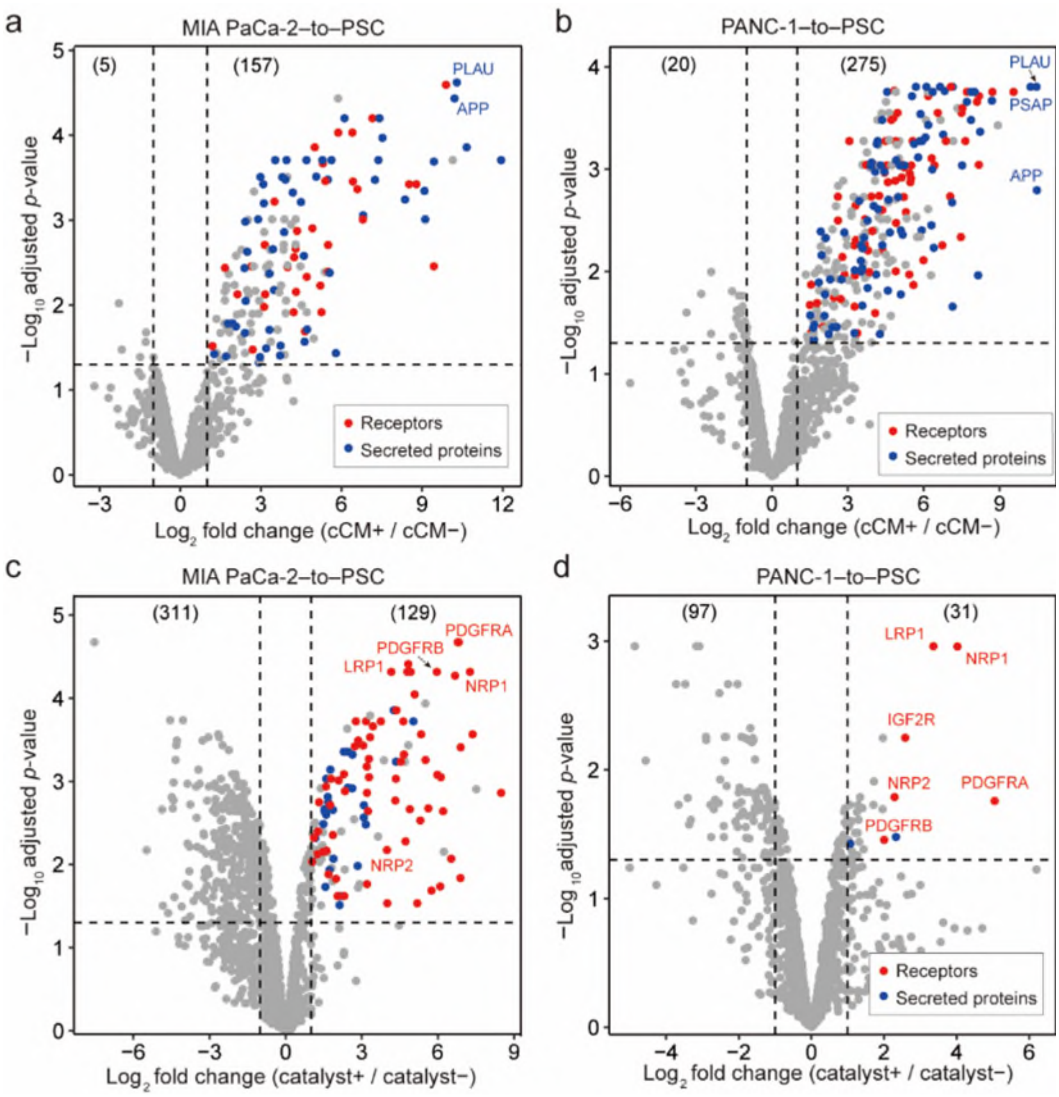

Supplementary Fig. 12 Identification of putative ligands and receptors in PCC-to-PSC paracrine signaling by Click-IGC approach. **a**, Volcano plot showing the MIA PaCa-2-secreted proteins bound to PSCs. **b**, the PANC-1-secreted proteins bound to PSCs. **c**, Click-IGC using MIA PaCa-2-cCM as ligands on PSC cells. **d**, Click-IGC using PANC-1-cCM as ligands on PSC cells. The significant secreted proteins and receptors annotated by UniProt are highlighted in blue and red, respectively. All experiments were performed in triplicate per condition.

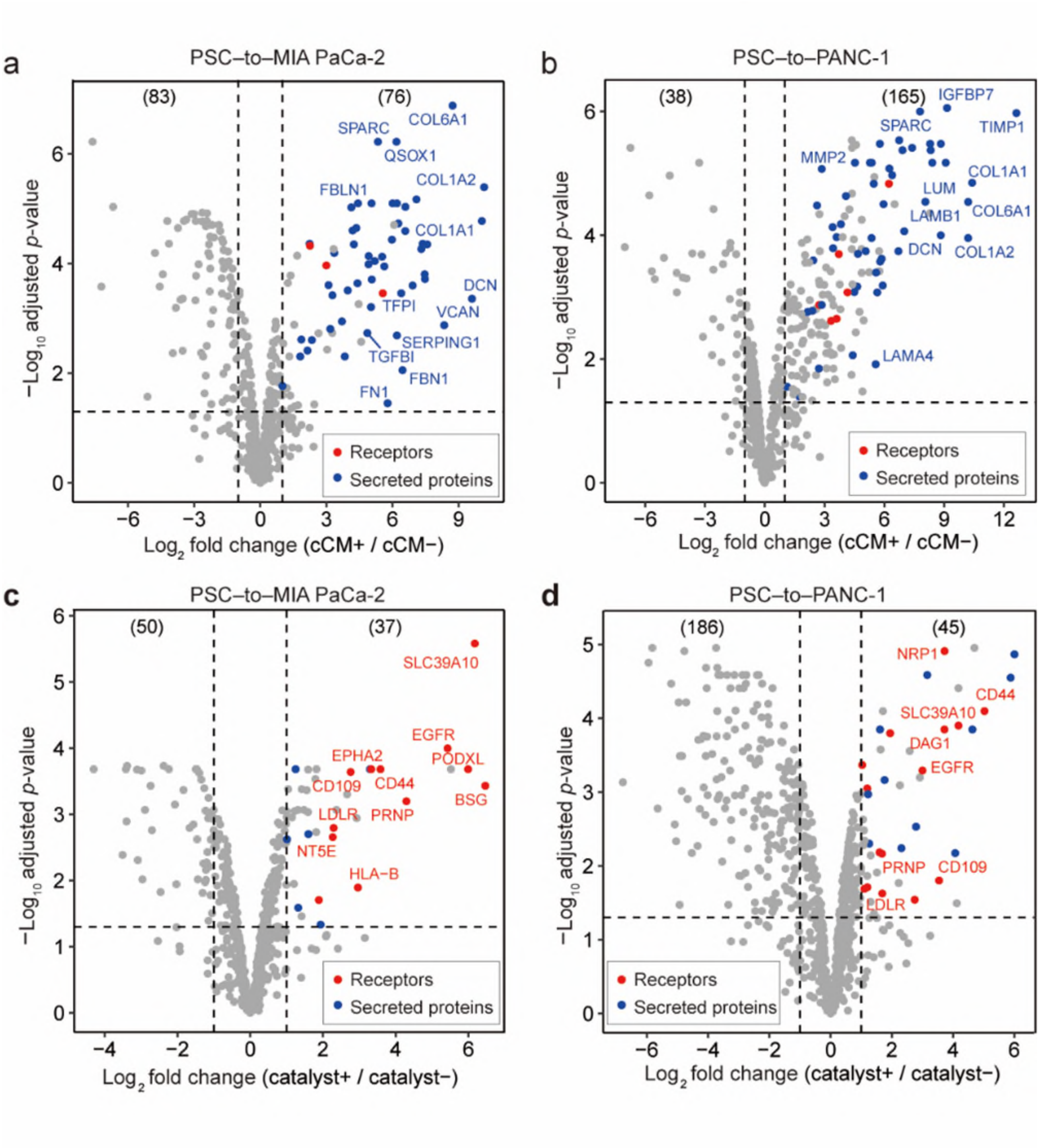

Supplementary Fig. 13 Identification of putative ligands and receptors in PSC-to-PCC paracrine signaling by Click-IGC approach. **a**, Volcano plots showing the PSC-secreted proteins bound to MIA PaCa-2 cells. **b**, PSC-secreted proteins bound to PANC-1 cells. **c**, Click-IGC using PSC-cCM as ligands revealing the interacting receptors on MIA PaCa-2 cells. **d**, Click-IGC using PSC-cCM as ligands on PANC-1 cells. The significant secreted proteins and receptors annotated by UniProt are highlighted in blue and red, respectively. All experiments were performed in triplicate per condition.

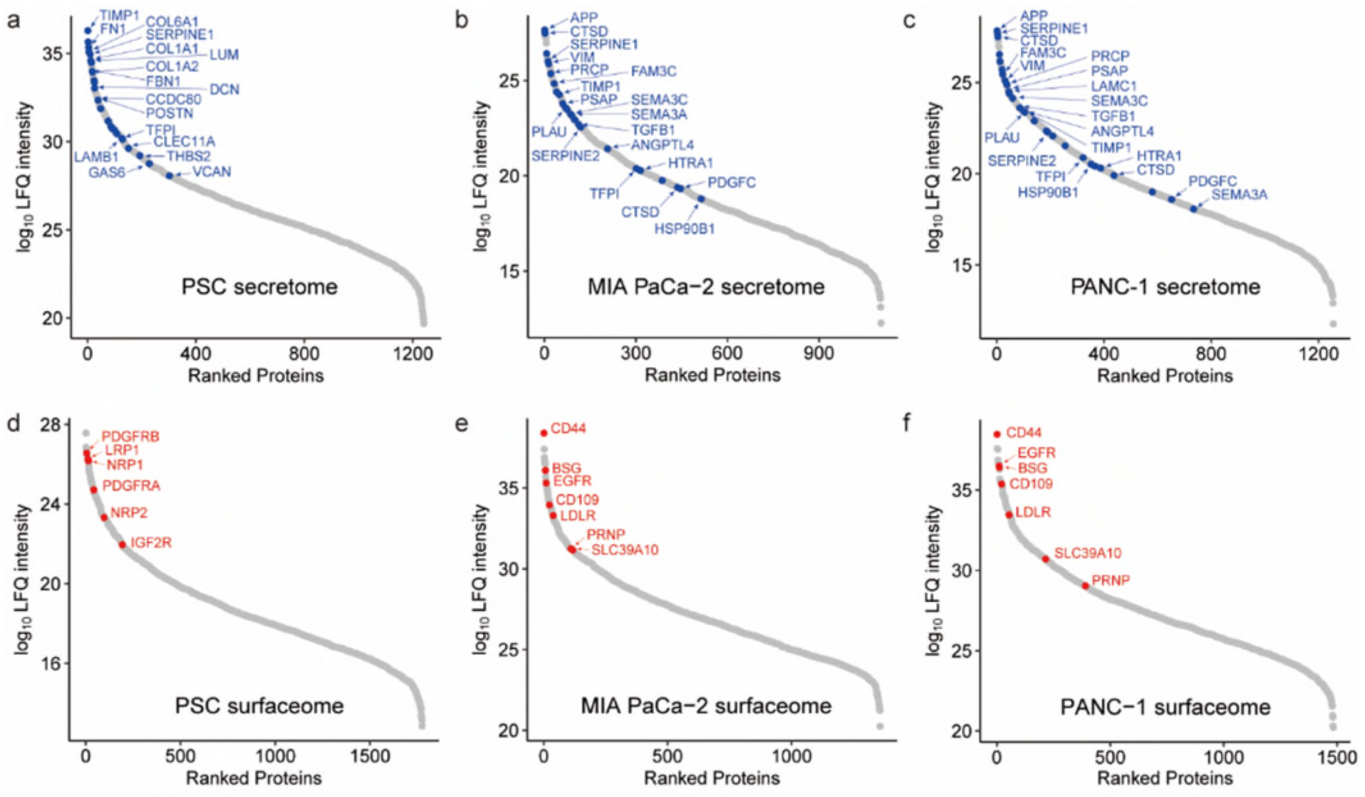

Supplementary Fig. 14 Label-free quantitation (LFQ) intensity-ranked sectretomes of (**a**) PSC, (**b**) MIA PaCa-2 and (**c**) PANC-1 cells. LFQ intensity-ranked sufaceomes of (**d**) PSC, (**e**) MIA PaCa-2 and (**f**) PANC-1 cells.

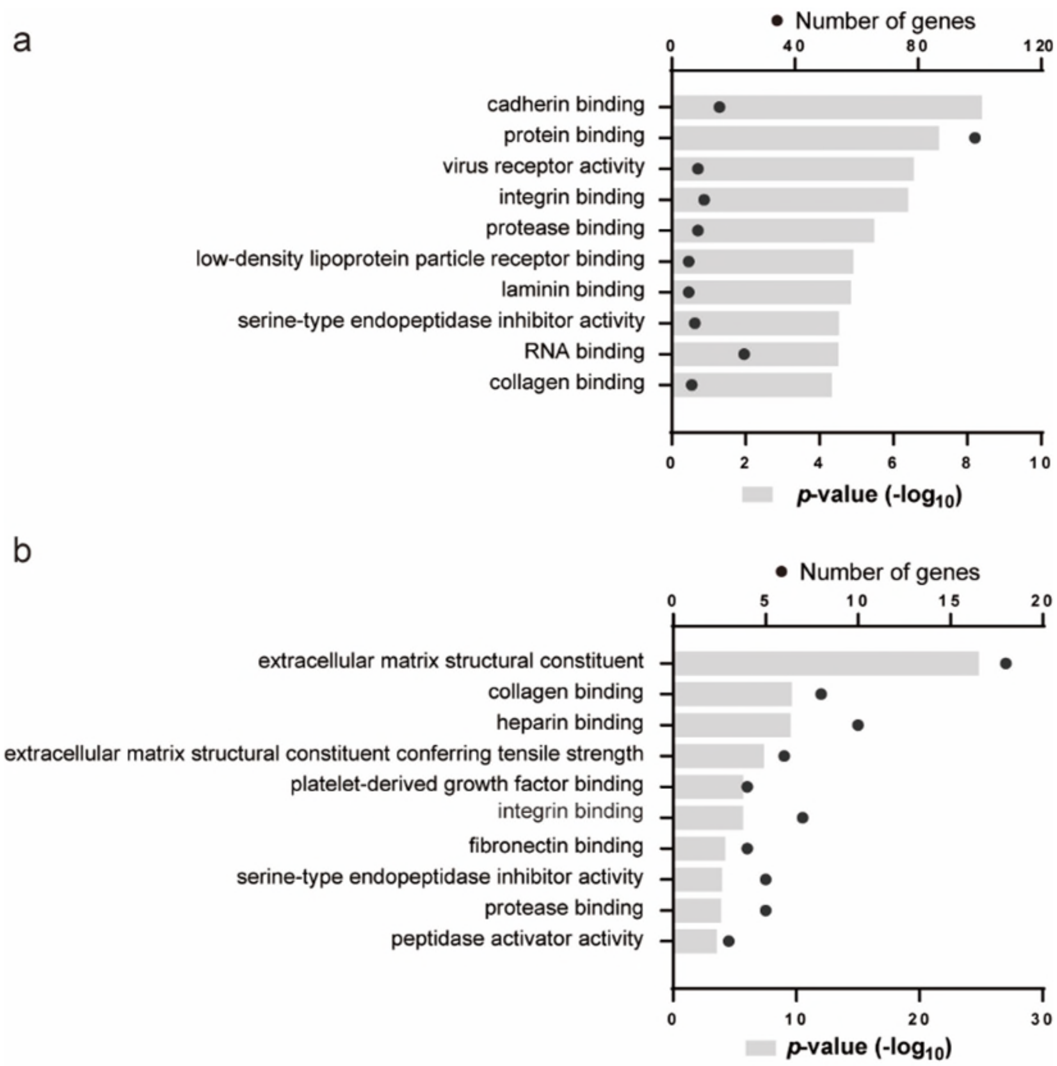

Supplementary Fig. 15 Gene ontology (GO) term enrichment analysis of the interacting proteins in CM of PCCs and PSCs. Top 10 GO Molecular Function terms for the significant proteins in (**a**) Supplementary Fig. 12a, b and (**b**) Supplementary Fig. 13a, b.

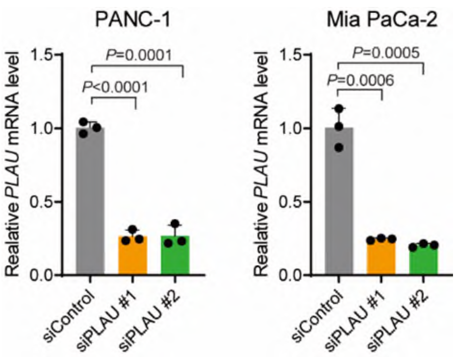

Supplementary Fig. 16 RT-qPCR detection of relative PLAU knockdown using negative control siRNA (siControl) or PLAU siRNA (PLAU). The *P* values were calculated using two-sided Student's *t*-test and data are presented as the mean ± SD (n = 3).

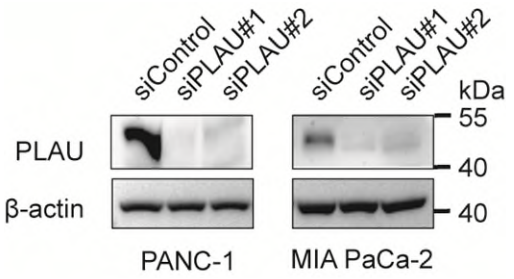

Supplementary Fig. 17 Western blot analysis of PLAU protein expression levels of PANC-1 and MIA PaCa-2 cells after siRNA treatment. Images are representative of 3 biological replicates. Source data are provided as Source Data file.

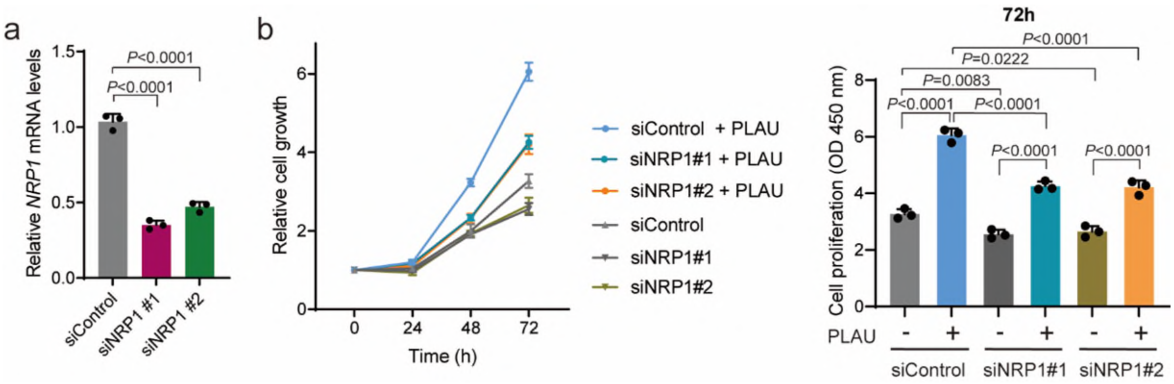

Supplementary Fig. 18 The effects of NRP1 knockdown and PLAU treatment on PSC cell growth. **a** PSCs transfected with negative control siRNA (siControl) or NRP1 siRNA (siNRP1) were subjected to RT-qPCR. The *P* values were calculated using two-sided Student's *t*-test and data are presented as the mean ± SD (n = 3). **b** CCK-8 analysis of PSC proliferation under NRP1 knockdown and PLAU treatment. Significance at the 72 h time point was calculated by the one-way ANOVA with Tukey's post hoc testing. Data are presented as the mean ± SD (n = 3 biological replicates).

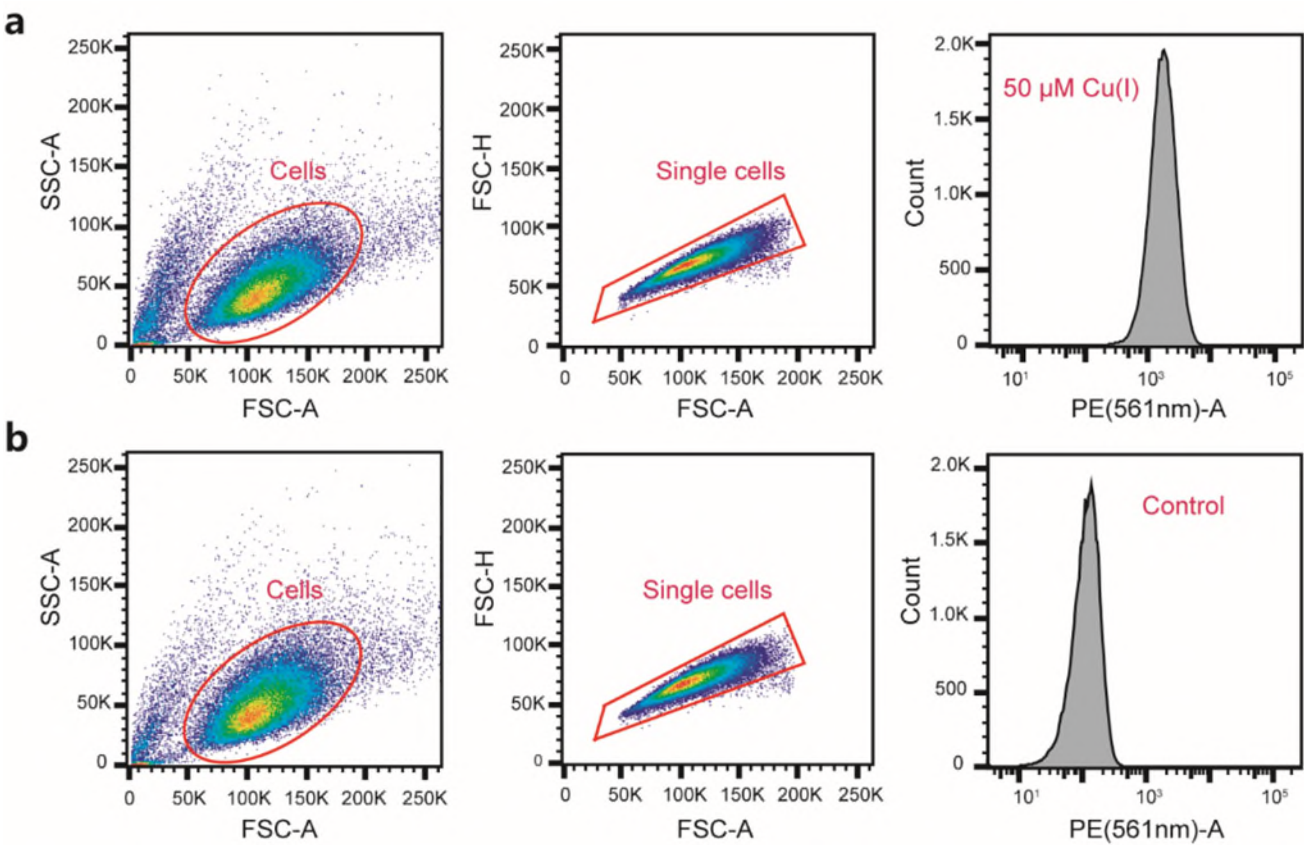

Supplementary Fig. 19 Gating of the probe labeled K562 cells and control cell populations. **a**, Gating of probe labeled K562 cells with 50 μM Cu(I); b, Gating of the K562 cells without Cu(I) catalyst. The strategy for subsequent removal of irregular events based on FlowJo V10, cell debris based on forward (FSC-A) and side (SSC-A) scatter, cell aggregates based on forward scatter height (FSC-H) and area (FSC-A) signal is shown.

MS Quality Control

Quality control of the MS data in Fig. 2b

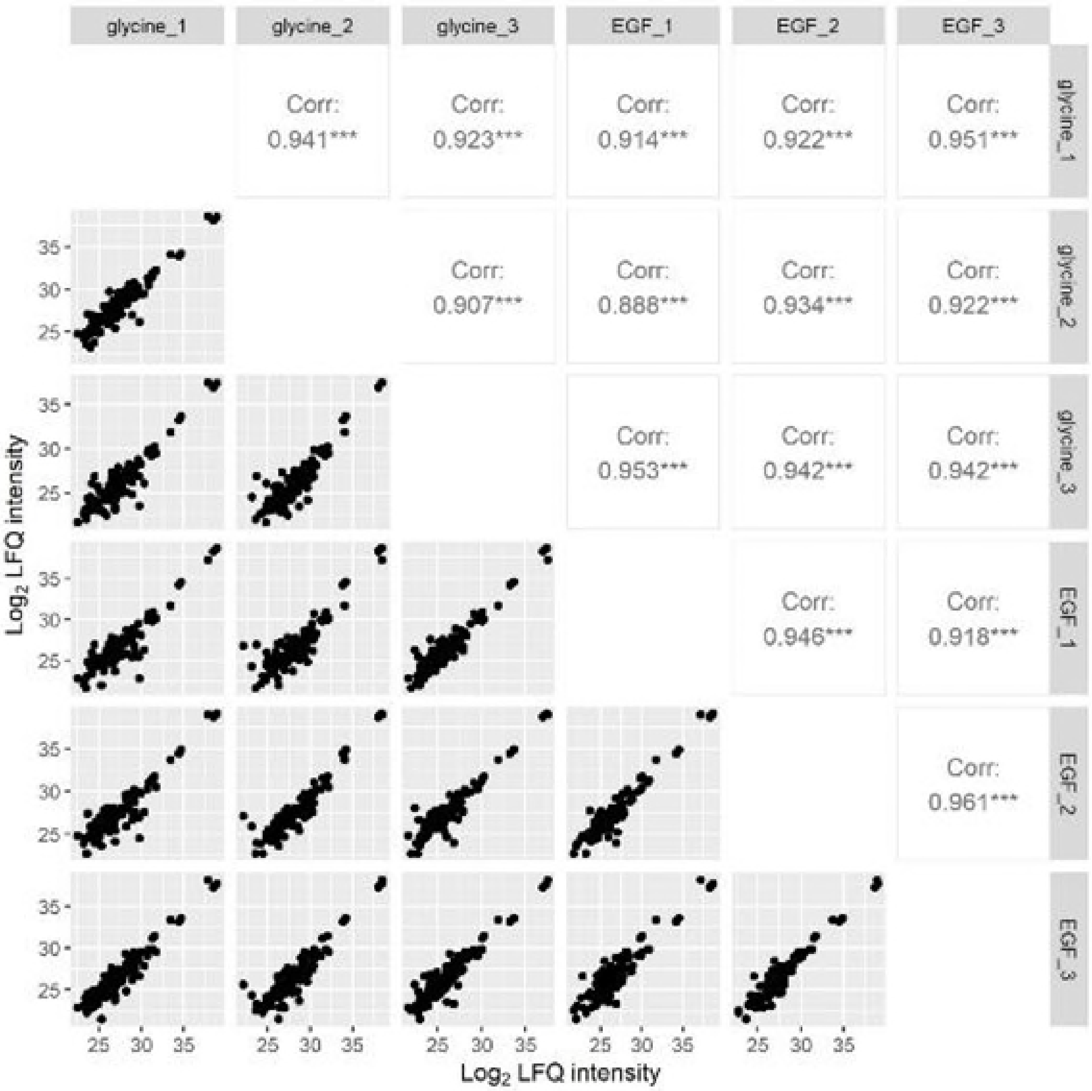

Supplementary Fig. 20 Pairwise Pearson correlation of the Log_2 transformed LFQ intensity before normalization and imputation.

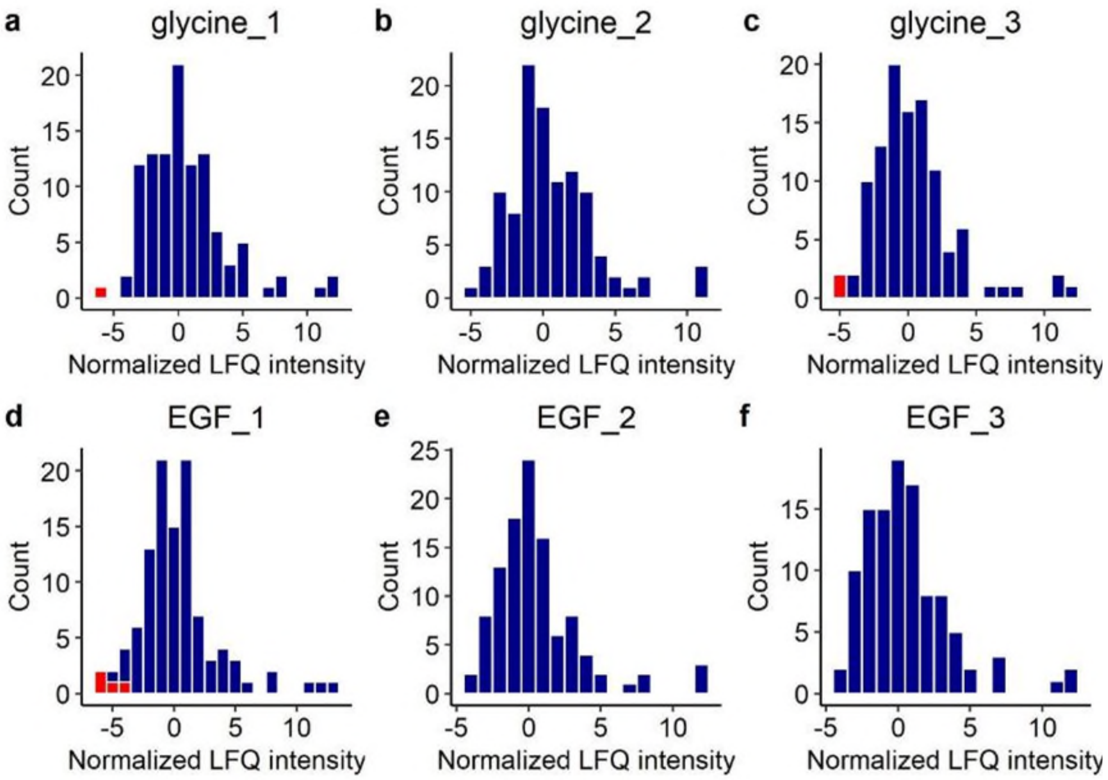

Supplementary Fig. 21 Histograms show the distribution of the normalized LFQ intensities in each sample (marked in blue), and the missing values that were imputed from the normal distribution are marked in red.

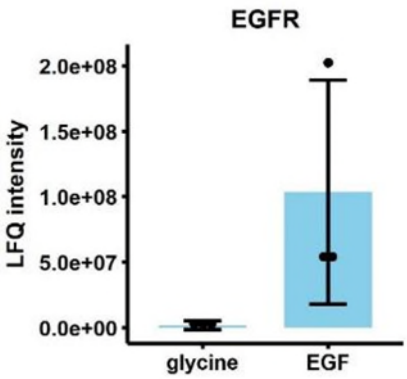

Supplementary Fig. 22 LFQ intensities of the significant receptors identified in the volcano plot. Data are presented as mean ± SD ($n = 3$ biological replicates).

Quality control of the MS data in Fig. 2c

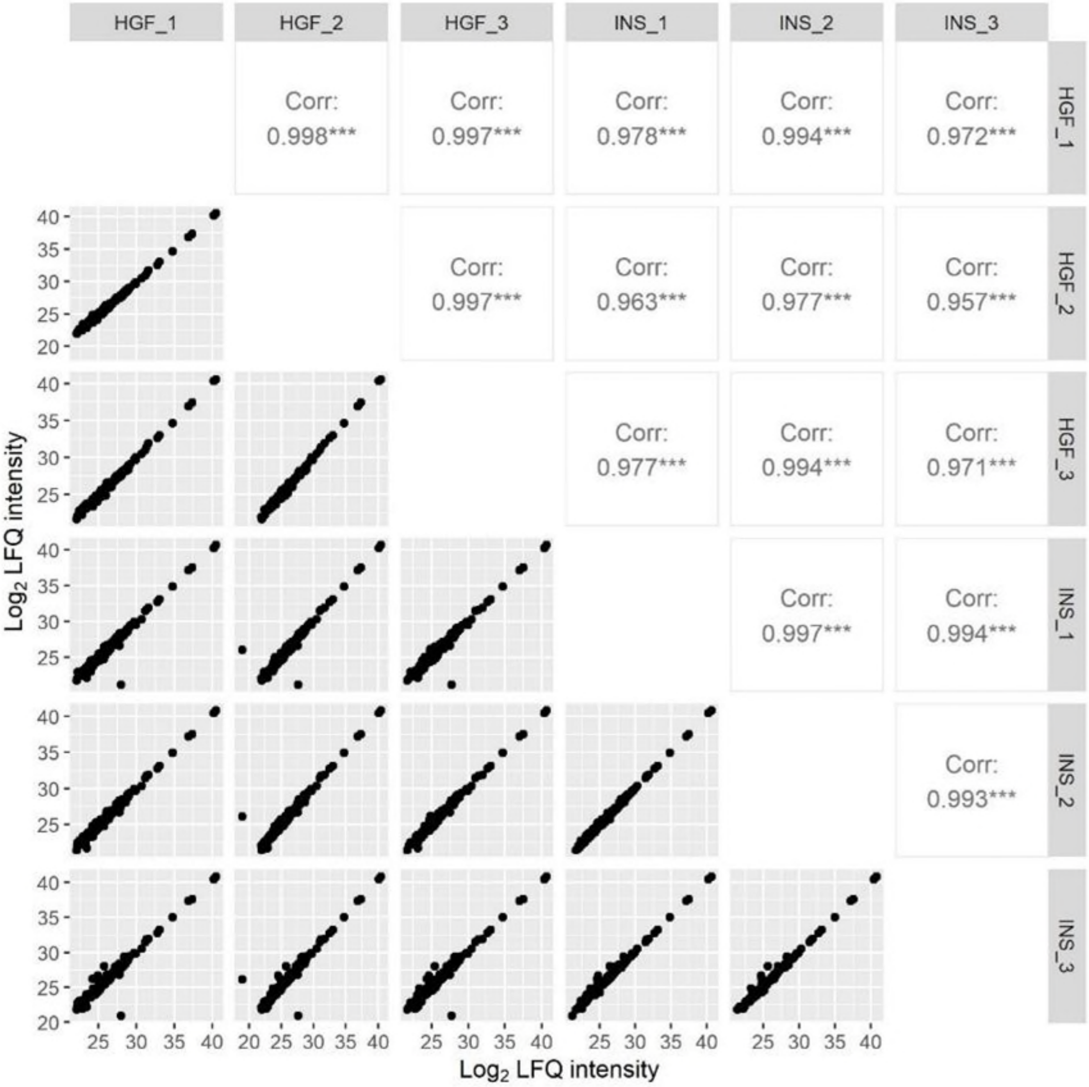

Supplementary Fig. 23 Pairwise Pearson correlation of the Log_2 transformed LFQ intensity before normalization and imputation.

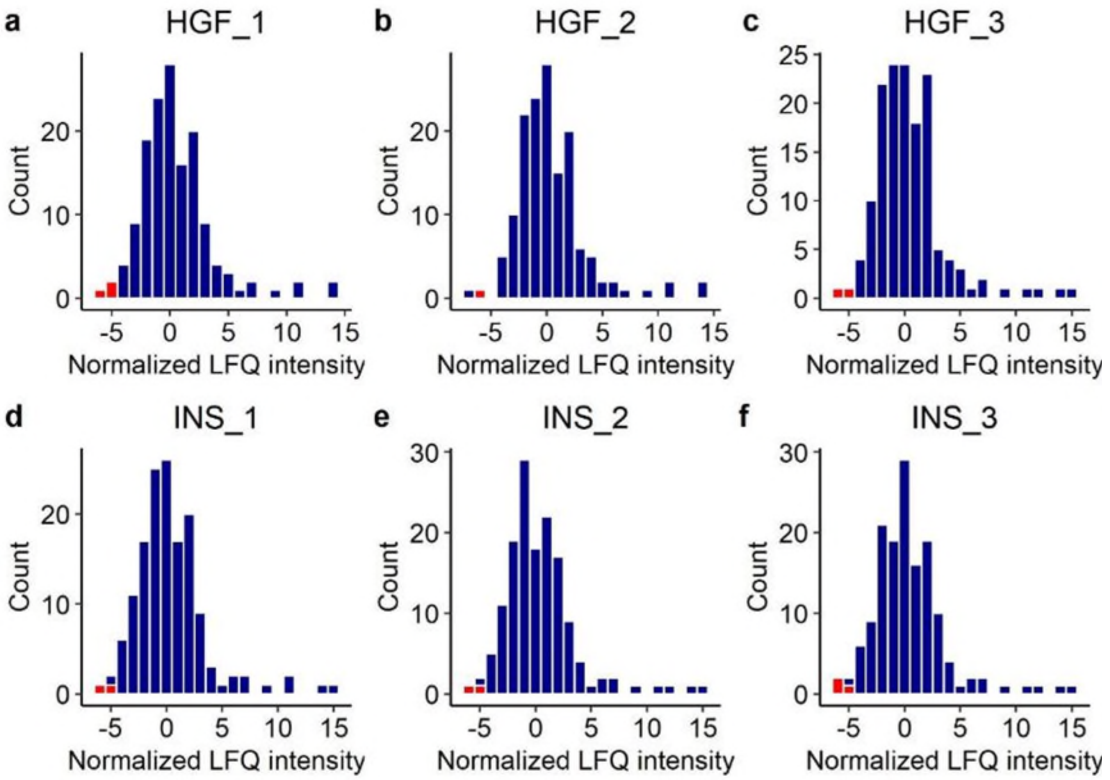

Supplementary Fig. 24 Histograms show the distribution of the normalized LFQ intensities in each sample (marked in blue), and the missing values that were imputed from the normal distribution are marked in red.

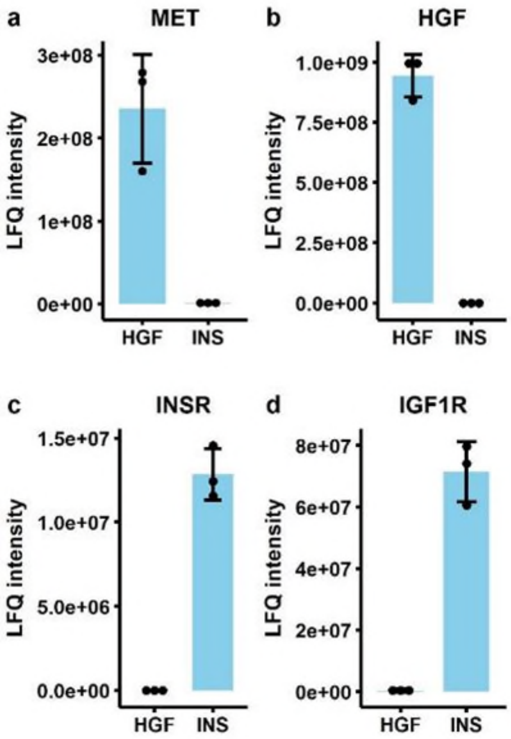

Supplementary Fig. 25 LFQ intensities of the significant receptors identified in the volcano plot. Data are

presented as mean ± SD ($n = 3$ biological replicates).

Quality control of the MS data in Fig. 2d

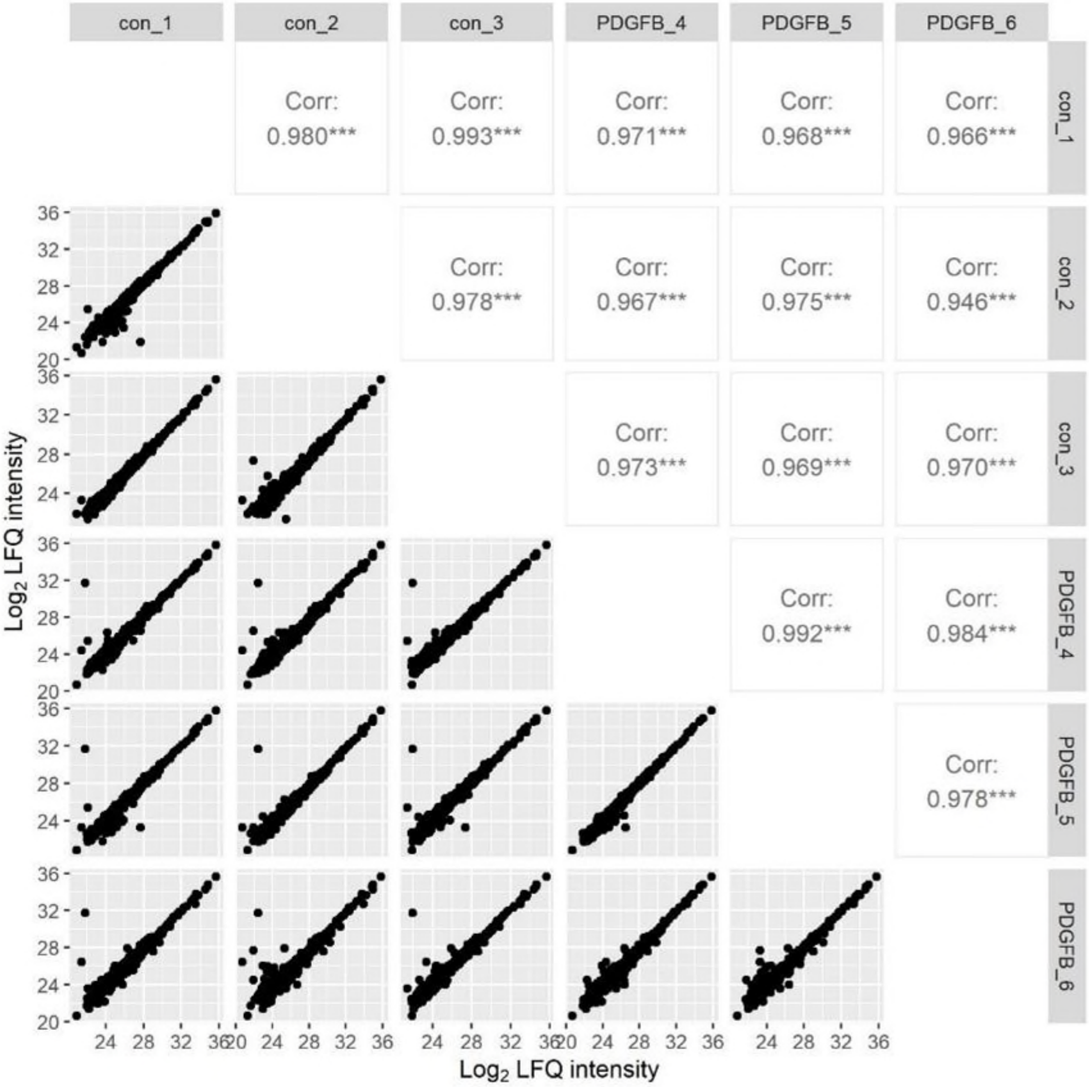

Supplementary Fig. 26 Pairwise Pearson correlation of the Log_2 transformed LFQ intensity before normalization and imputation.

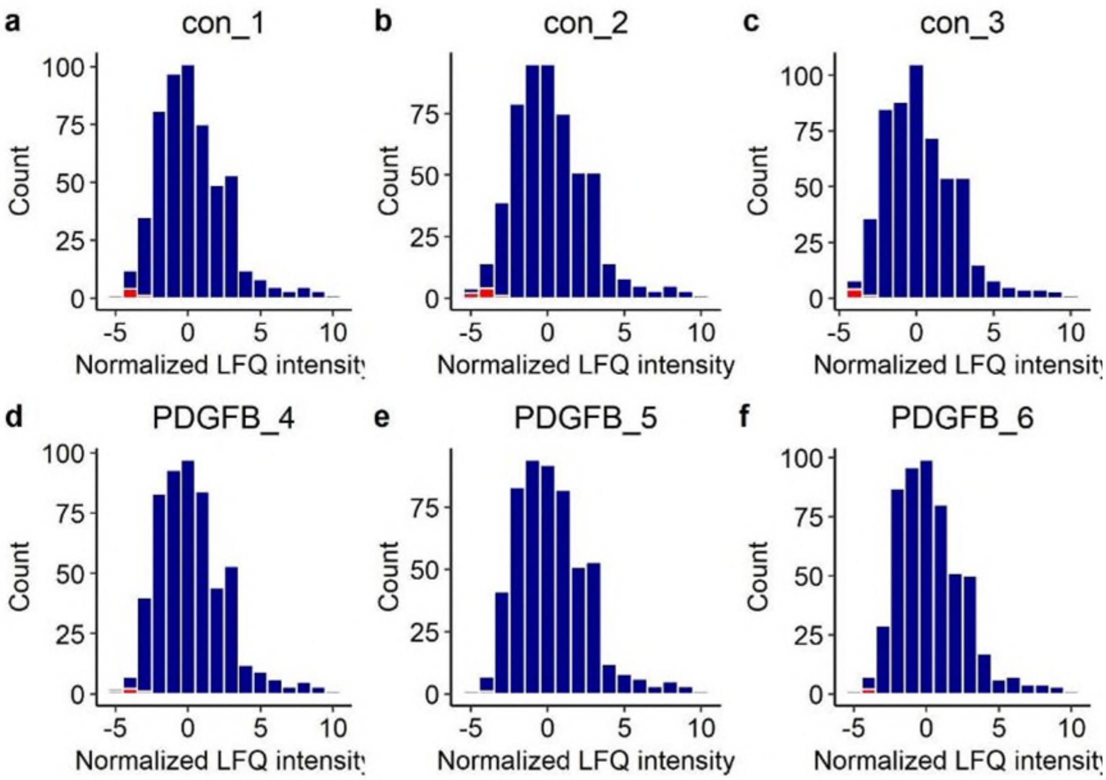

Supplementary Fig. 27 Histograms show the distribution of the normalized LFQ intensities in each sample (marked in blue), and the missing values that were imputed from the normal distribution are marked in red.

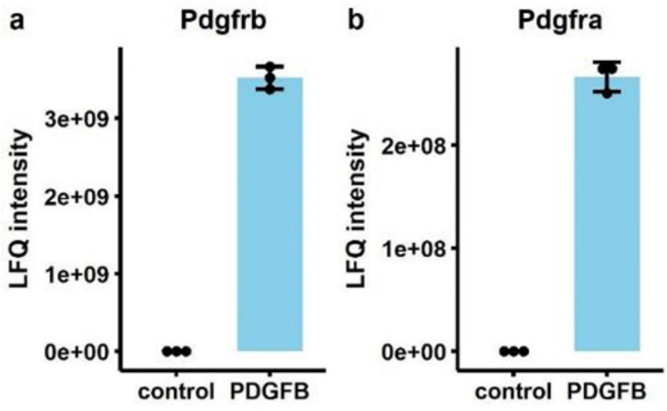

Supplementary Fig. 28 LFQ intensities of the significant receptors identified in the volcano plot. Data are presented as mean ± SD ($n = 3$ biological replicates).

Quality control of the MS data in Fig. 2e

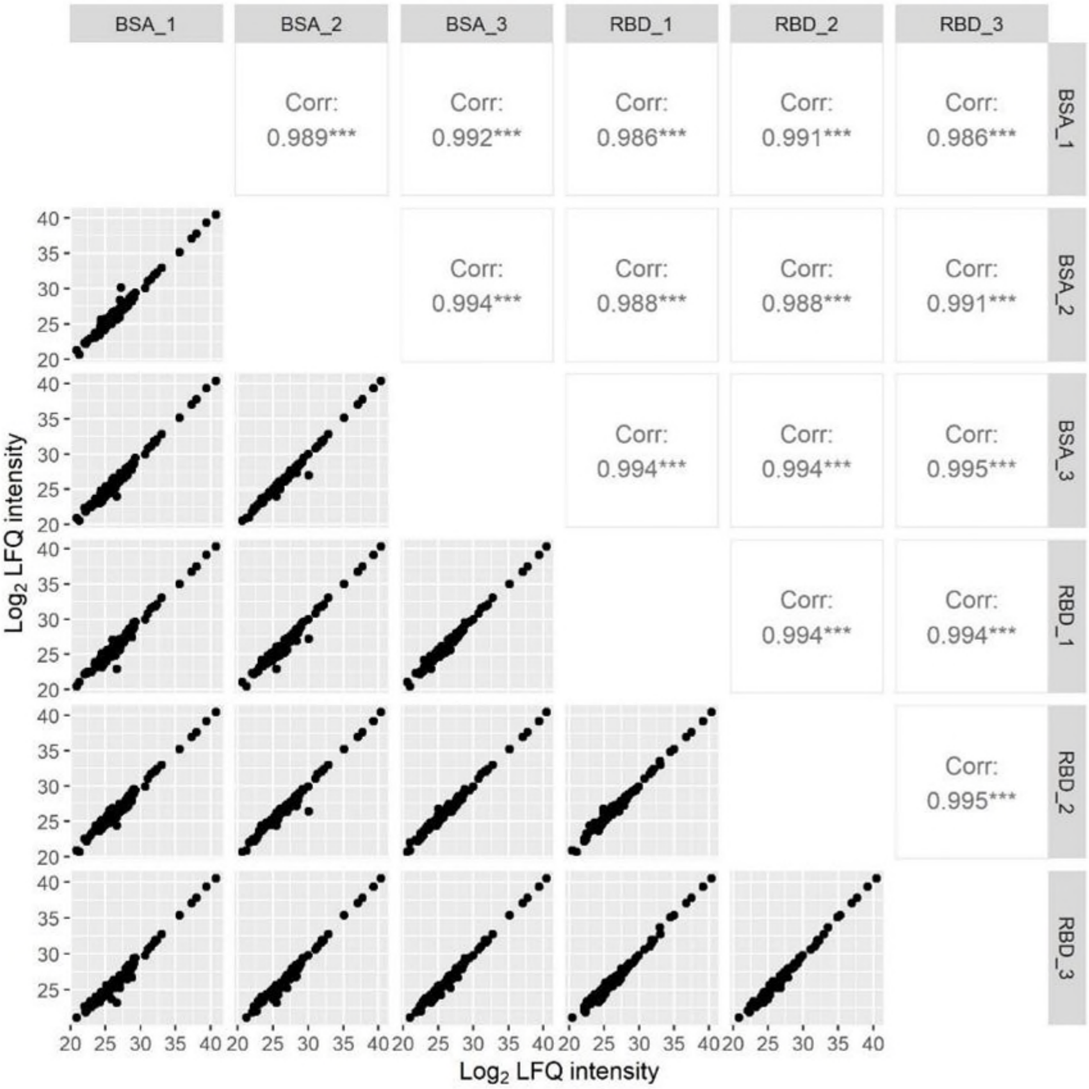

Supplementary Fig. 29 Pairwise Pearson correlation of the Log_2 transformed LFQ intensity before normalization and imputation.

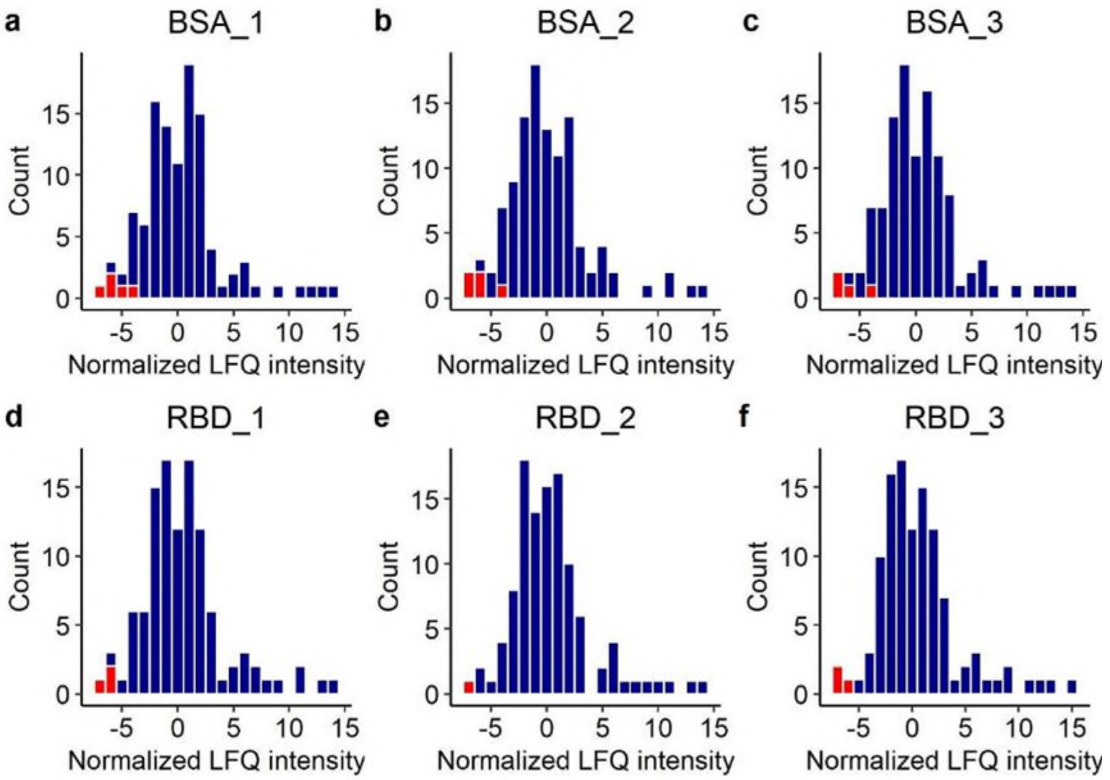

Supplementary Fig. 30 Histograms show the distribution of the normalized LFQ intensities in each sample (marked in blue), and the missing values that were imputed from the normal distribution are marked in red.

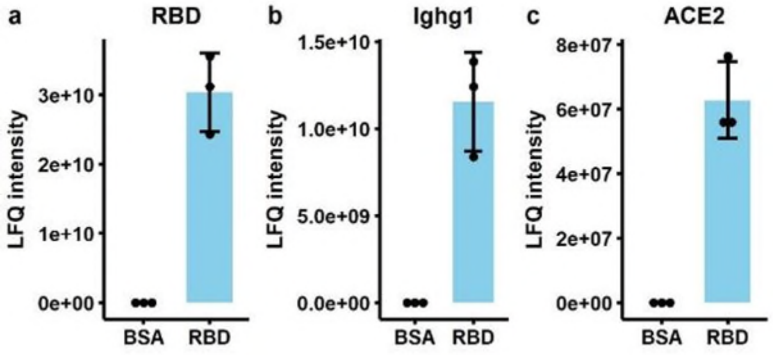

Supplementary Fig. 31 LFQ intensities of the significant ligands and receptors in the volcano plot. Data are presented as mean ± SD ($n = 3$ biological replicates).

Quality control of the MS data in Fig. 3b

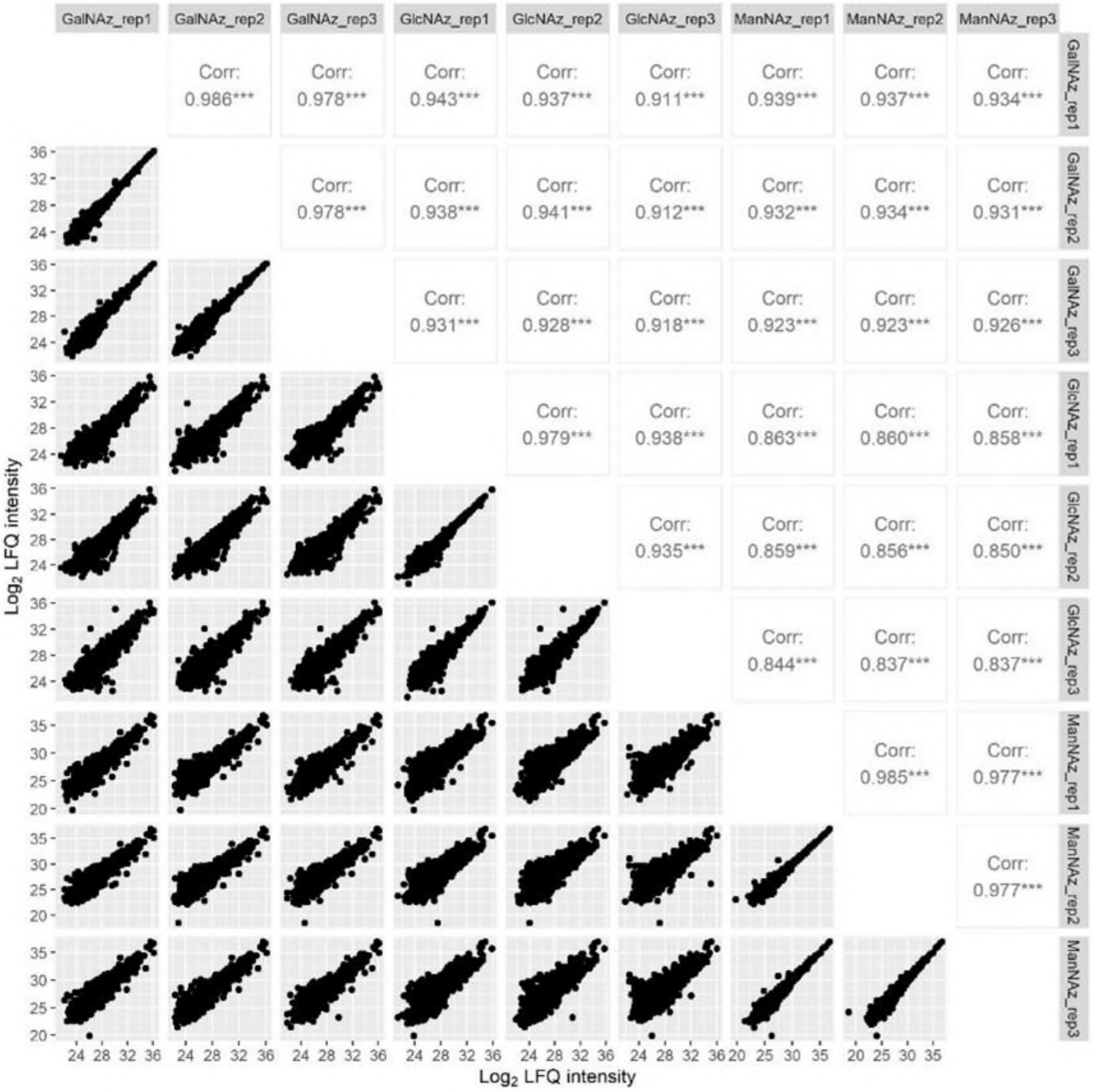

Supplementary Fig. 32 Pairwise Pearson correlation of the Log_2 transformed LFQ intensity before normalization and imputation.

Quality control of the MS data in Fig. 3d

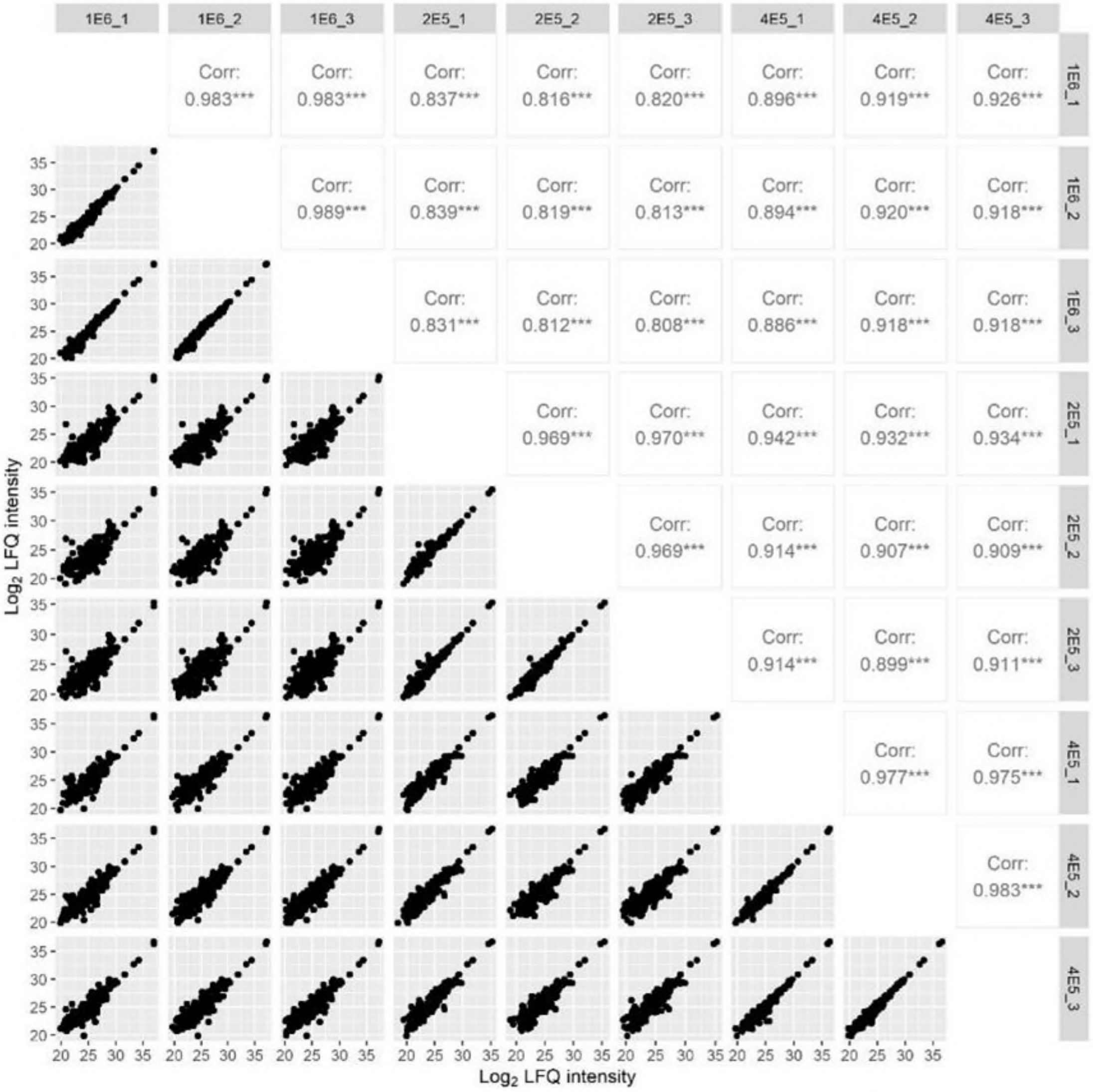

Supplementary Fig. 33 Pairwise Pearson correlation of the Log_2 transformed LFQ intensity before normalization and imputation.

Quality control of the MS data in Fig. 3f

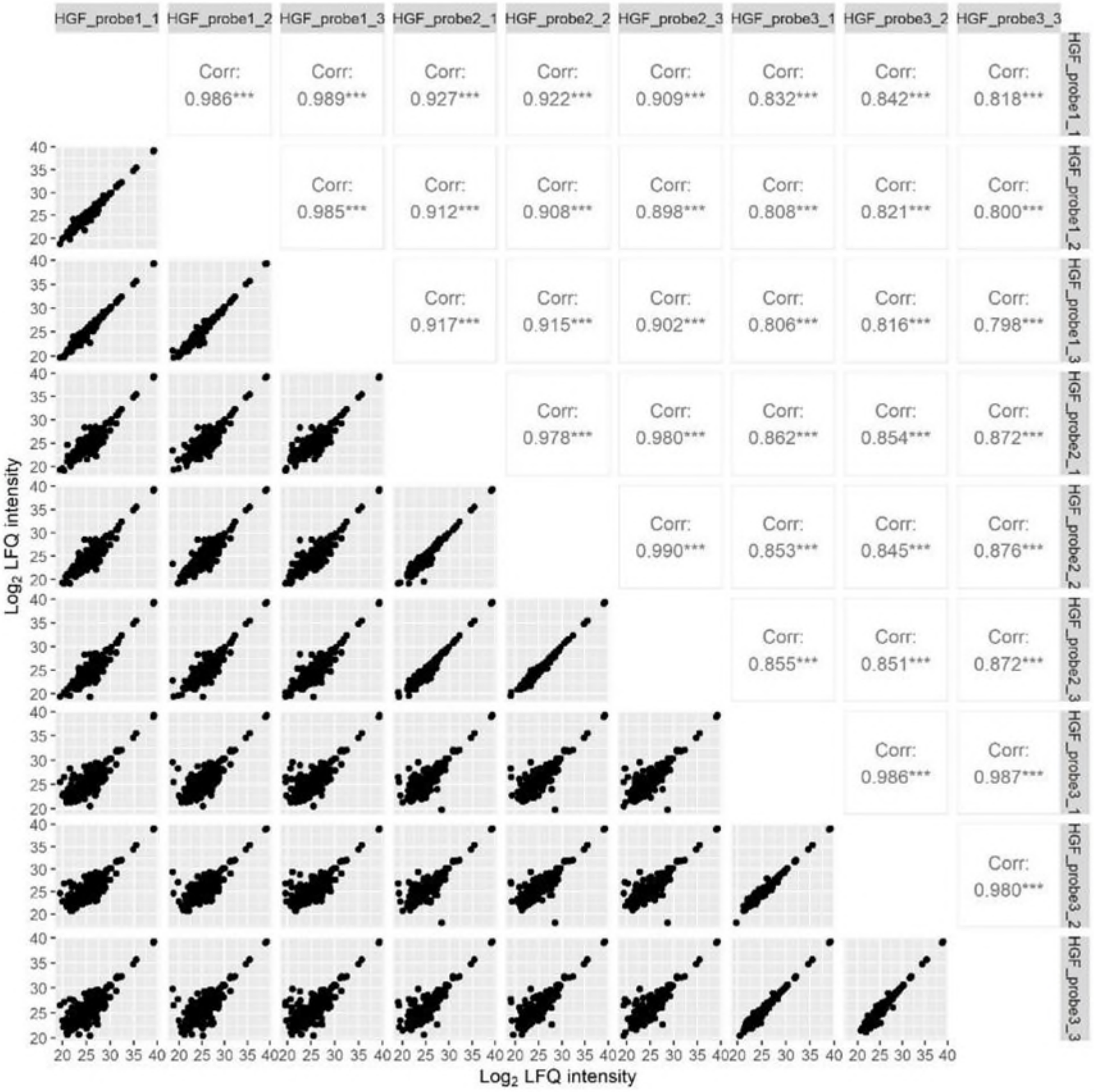

Supplementary Fig. 34 Pairwise Pearson correlation of the Log_2 transformed LFQ intensity before normalization and imputation. Samples for HGF receptor identification were analyzed.

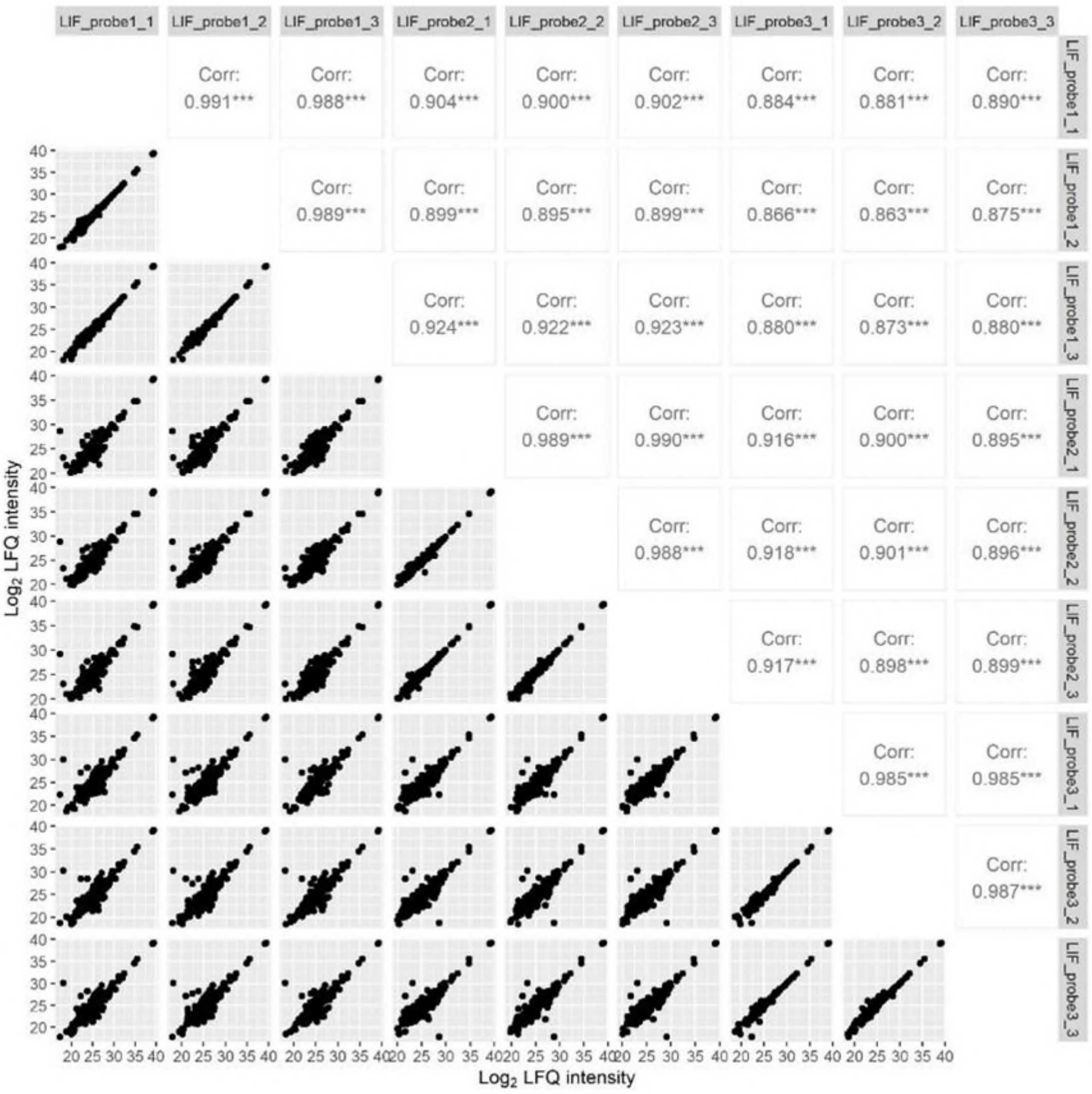

Supplementary Fig. 35 Pairwise Pearson correlation of the Log_2 transformed LFQ intensity before normalization and imputation. Samples for LIF receptor identification were analyzed.

Quality control of the MS data in Fig. 3e

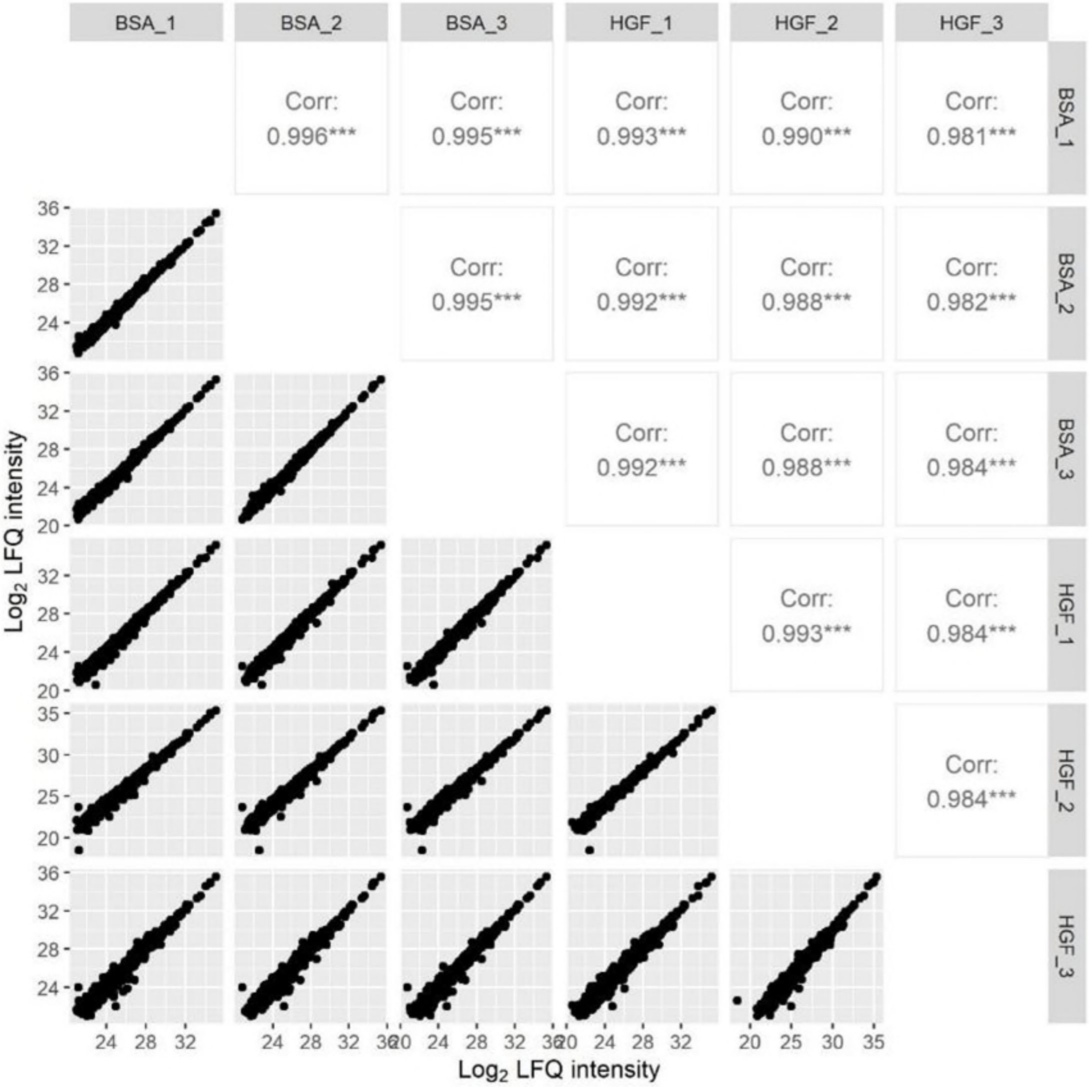

Supplementary Fig. 36 Pairwise Pearson correlation of the Log_2 transformed LFQ intensity before normalization and imputation.

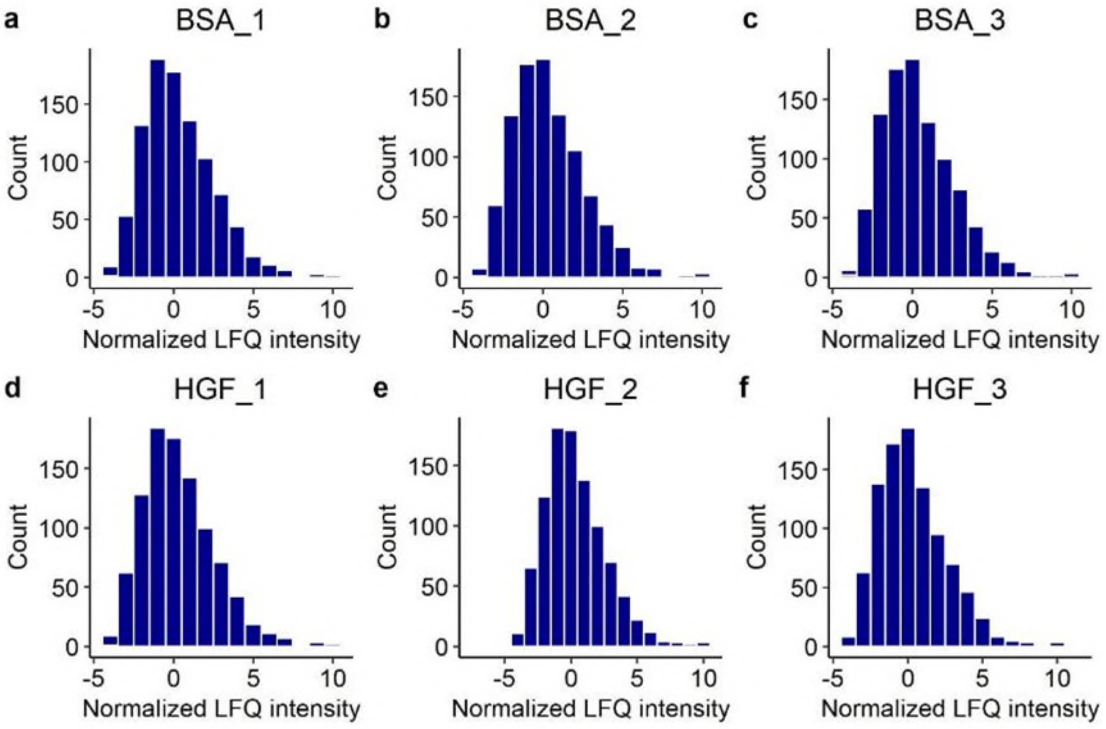

Supplementary Fig. 37 Histograms show the distribution of the normalized LFQ intensities in each sample (marked in blue), and the missing values that were imputed from the normal distribution are marked in red.

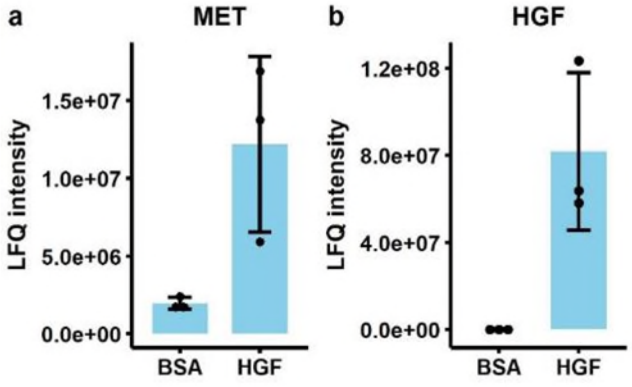

Supplementary Fig. 38 LFQ intensities of the significant ligand and receptor in the volcano plot. Data are presented as mean ± SD ($n = 3$ biological replicates).

Quality control of the MS data in Fig. 4b

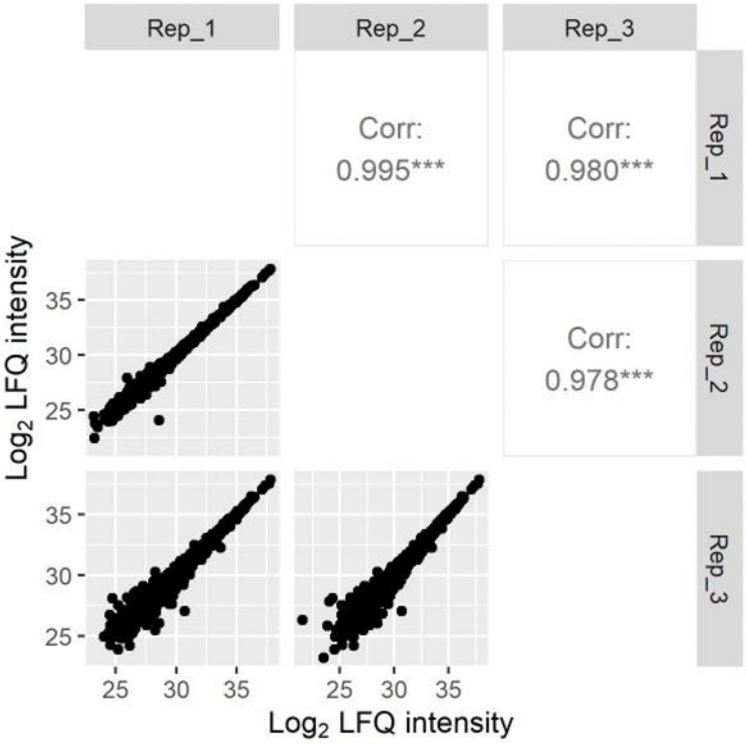

Supplementary Fig. 39 Pairwise Pearson correlation of the Log_2 transformed LFQ intensity before normalization and imputation.

Quality control of the MS data in Fig. 4c

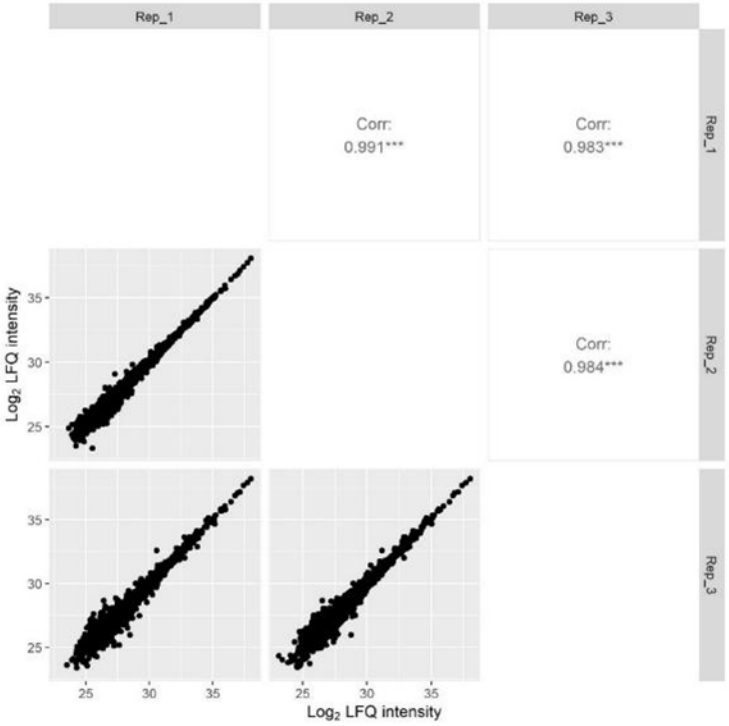

Supplementary Fig. 40 Pairwise Pearson correlation of the Log_2 transformed LFQ intensity before normalization and imputation.

Quality control of the MS data in Fig. 4d

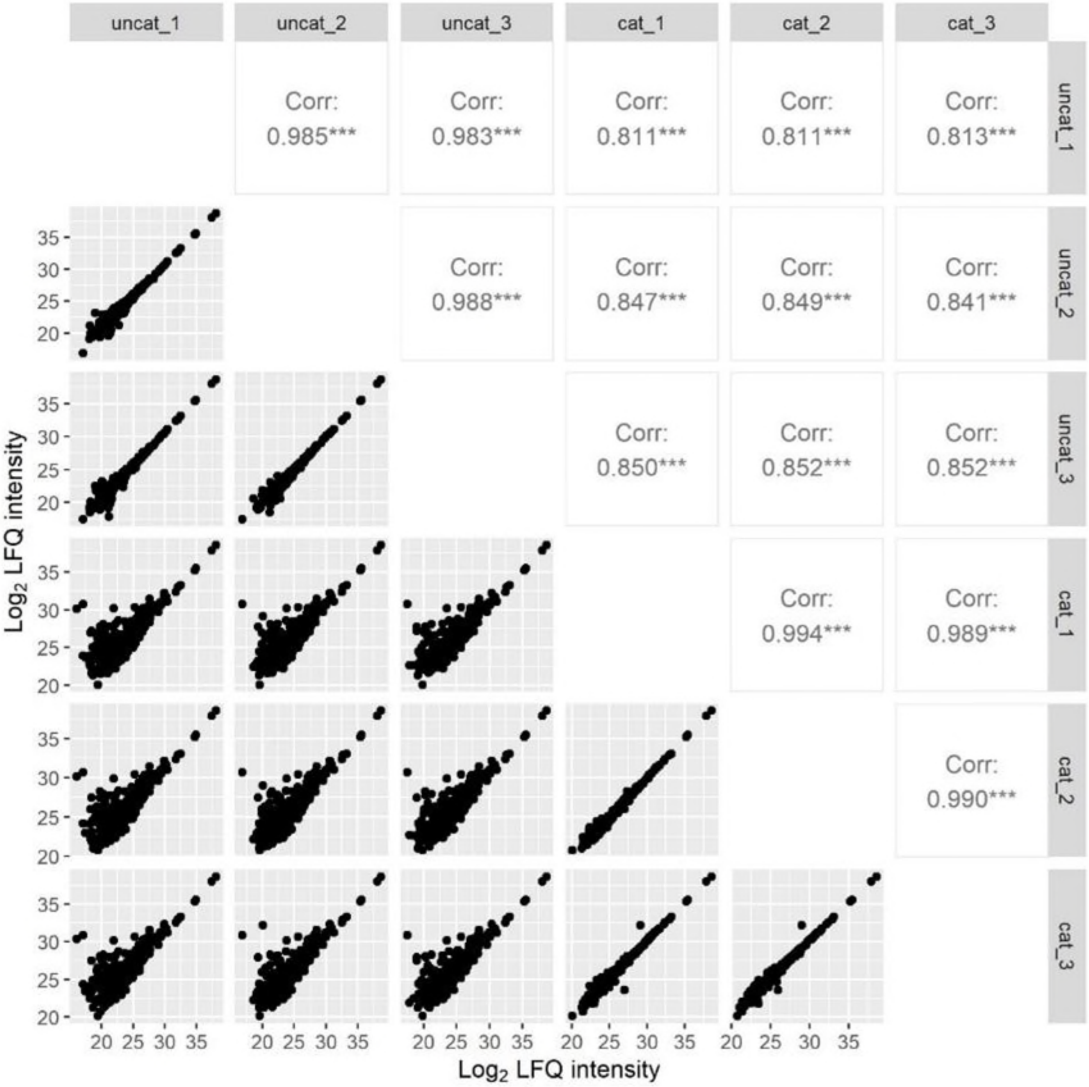

Supplementary Fig. 41 Pairwise Pearson correlation of the Log_2 transformed LFQ intensity before normalization and imputation.

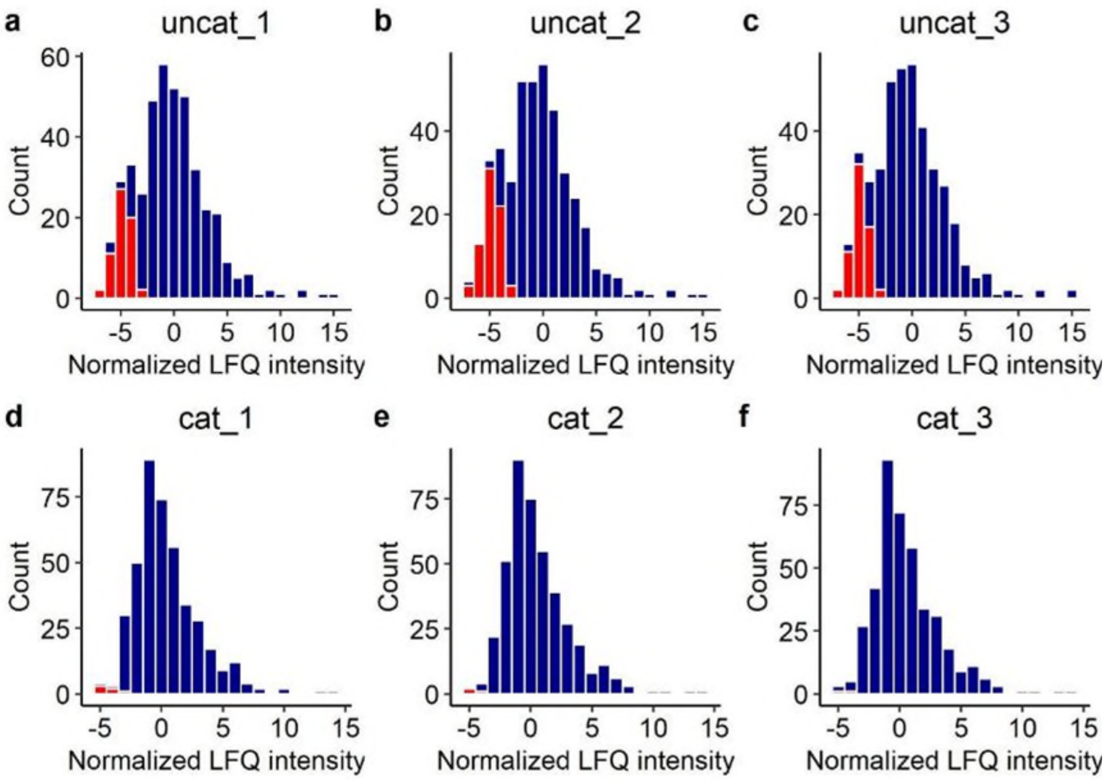

Supplementary Fig. 42 Histograms show the distribution of the normalized LFQ intensities in each sample (marked in blue), and the missing values that were imputed from the normal distribution are marked in red.

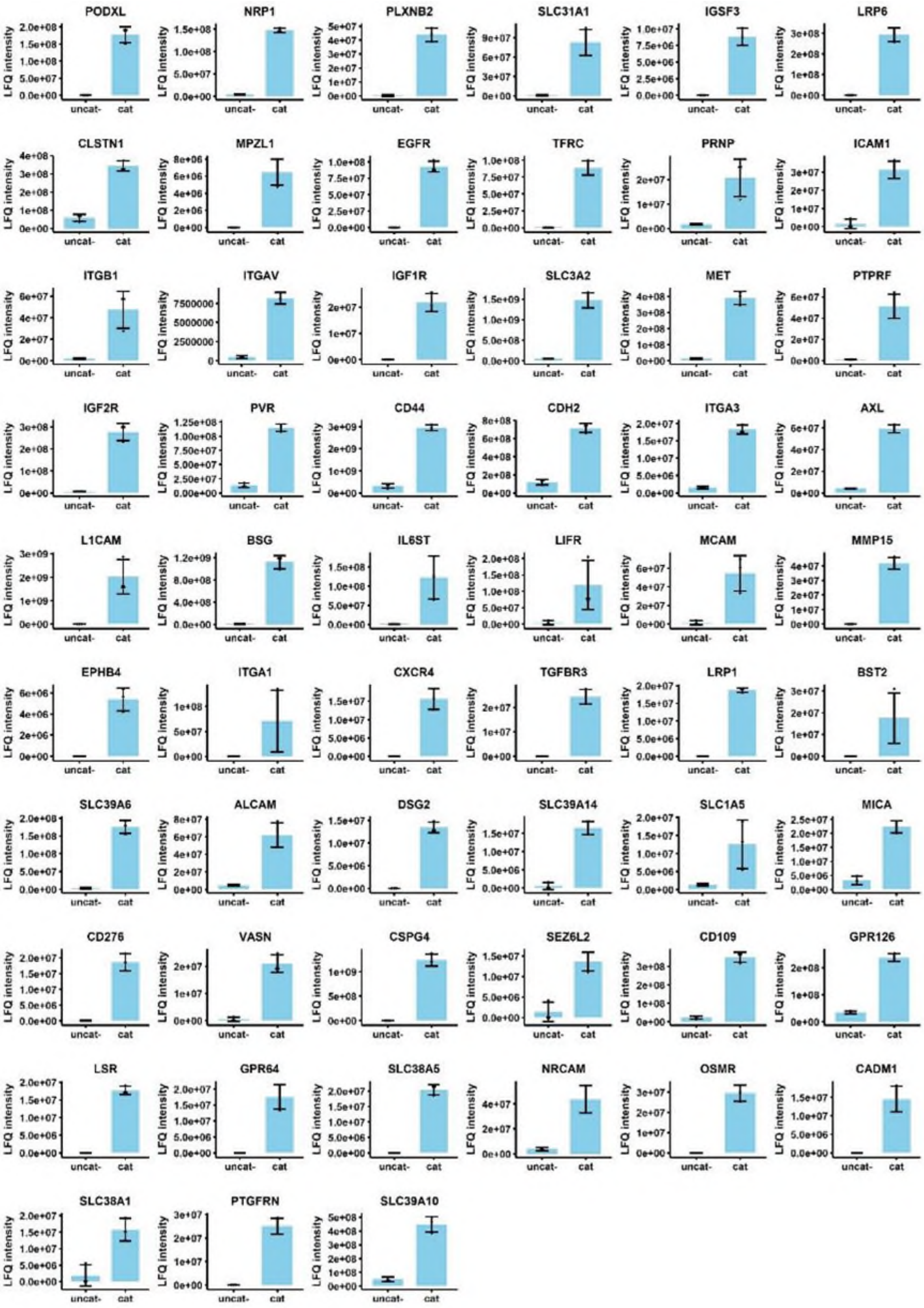

Supplementary Fig. 43 LFQ intensities of the significant receptors identified in the volcano plot. Data are presented as mean ± SD ($n = 3$ biological replicates).

Quality control of the MS data in Fig. 6d

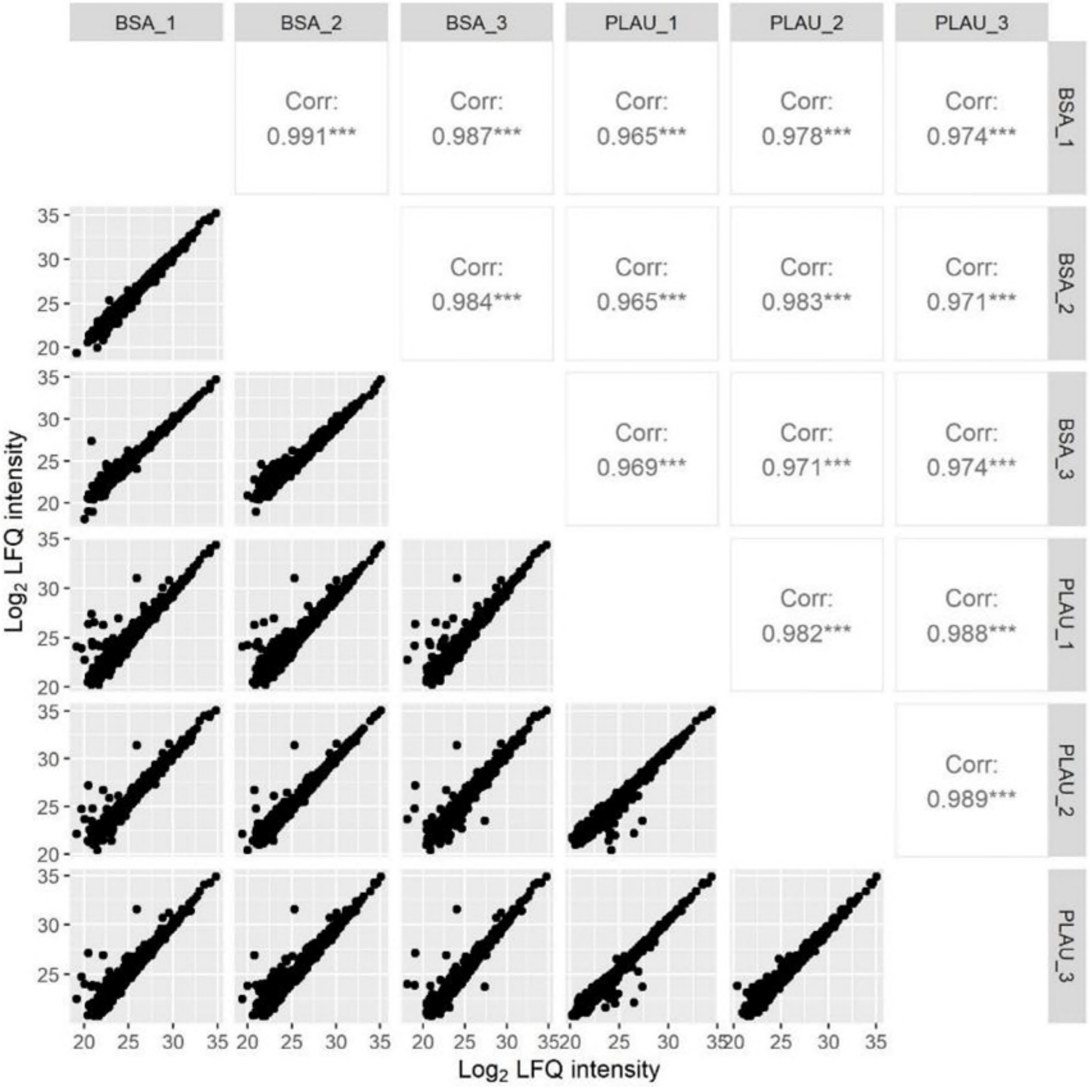

Supplementary Fig. 44 Pairwise Pearson correlation of the Log_2 transformed LFQ intensity before normalization and imputation.

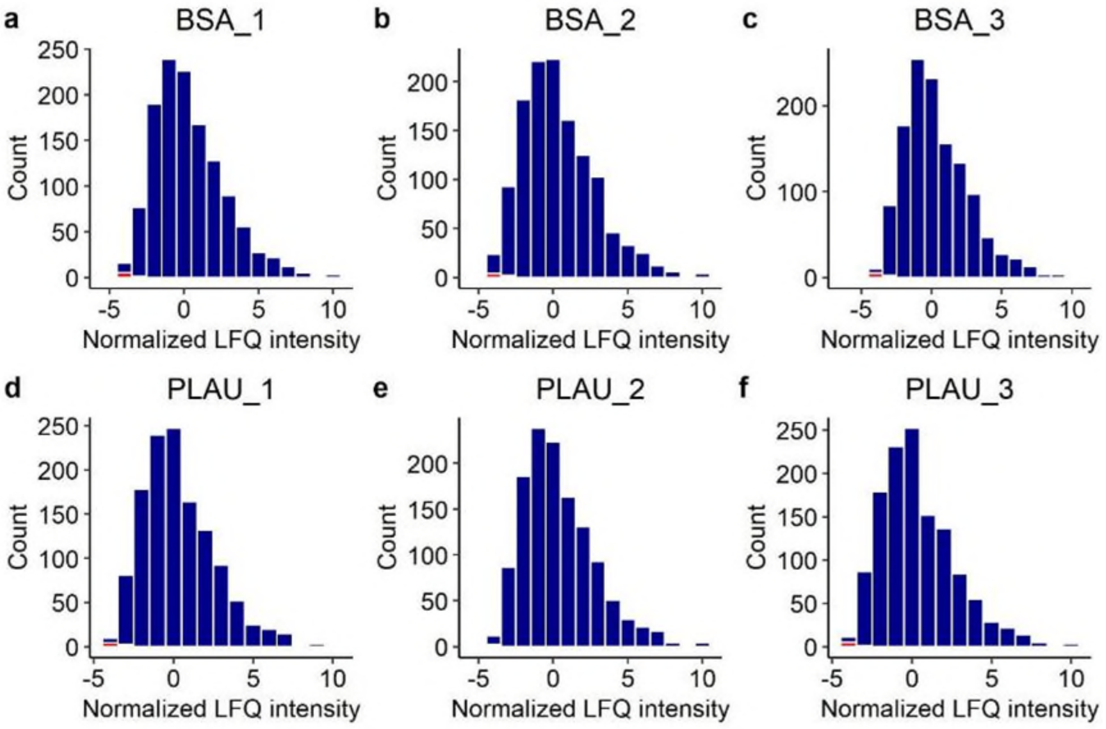

Supplementary Fig. 45 Histograms show the distribution of the normalized LFQ intensities in each sample (marked in blue), and the missing values that were imputed from the normal distribution are marked in red.

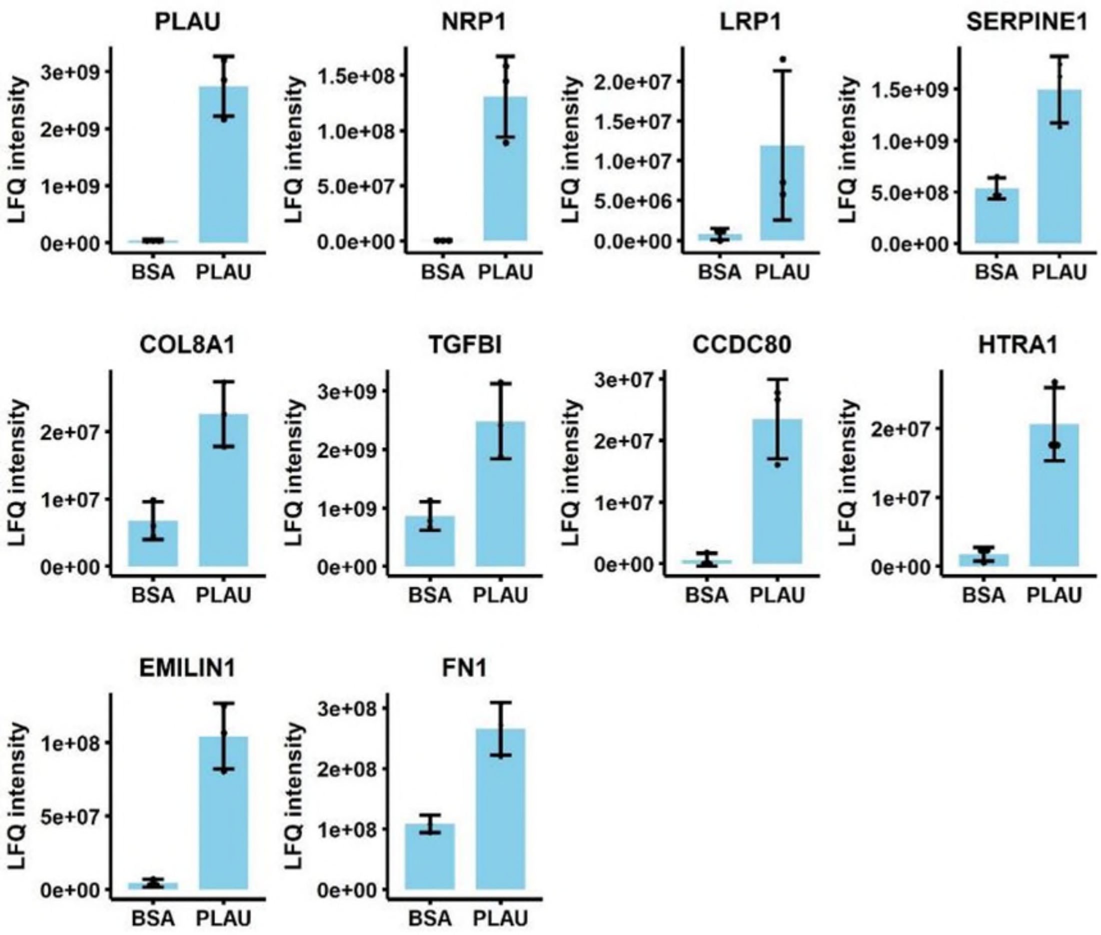

Supplementary Fig. 46 LFQ intensities of the significant ligands and receptors in the volcano plot. Data are presented as mean ± SD ($n = 3$ biological replicates).

Quality control of the MS data in Fig. 6e

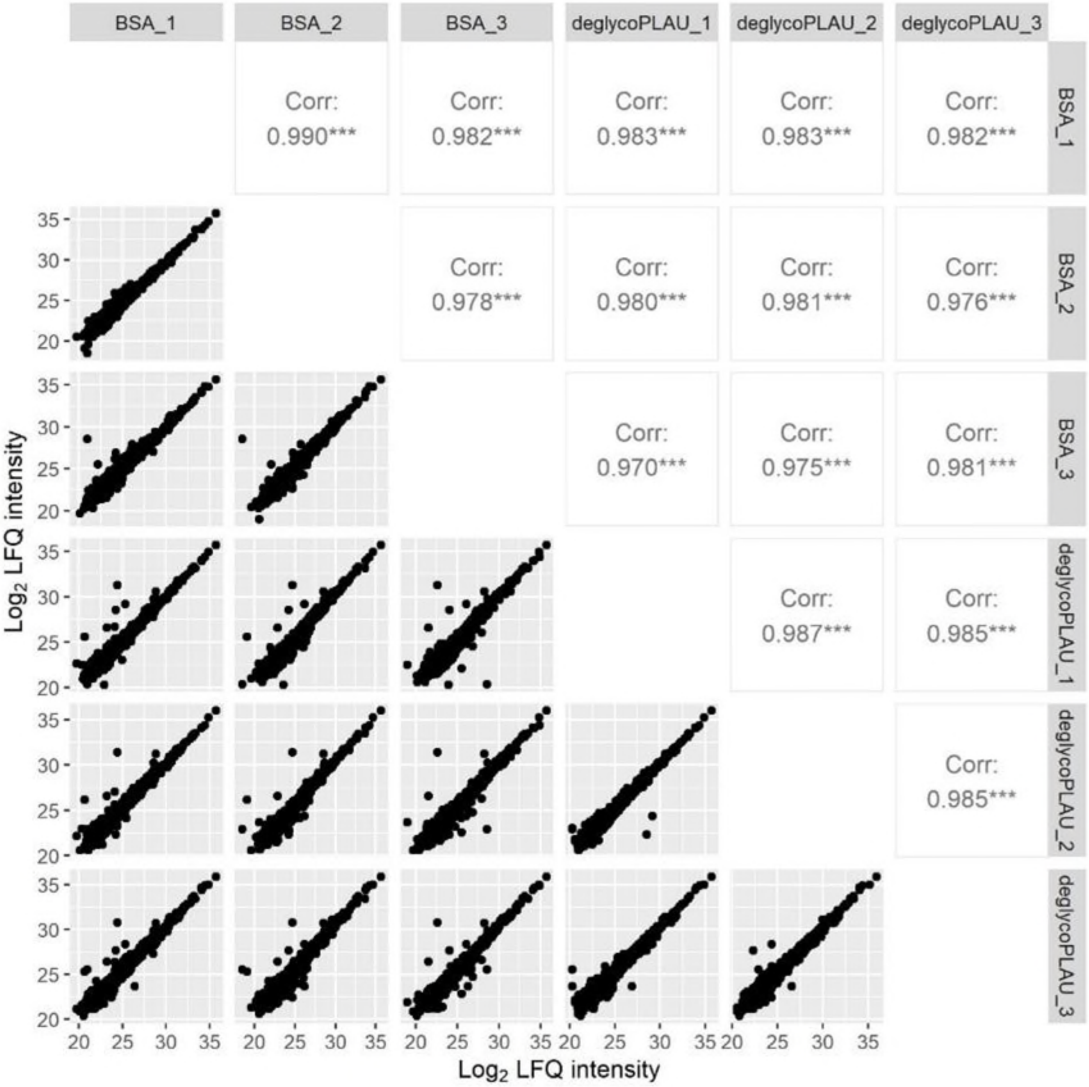

Supplementary Fig. 47 Pairwise Pearson correlation of the Log_2 transformed LFQ intensity before normalization and imputation.

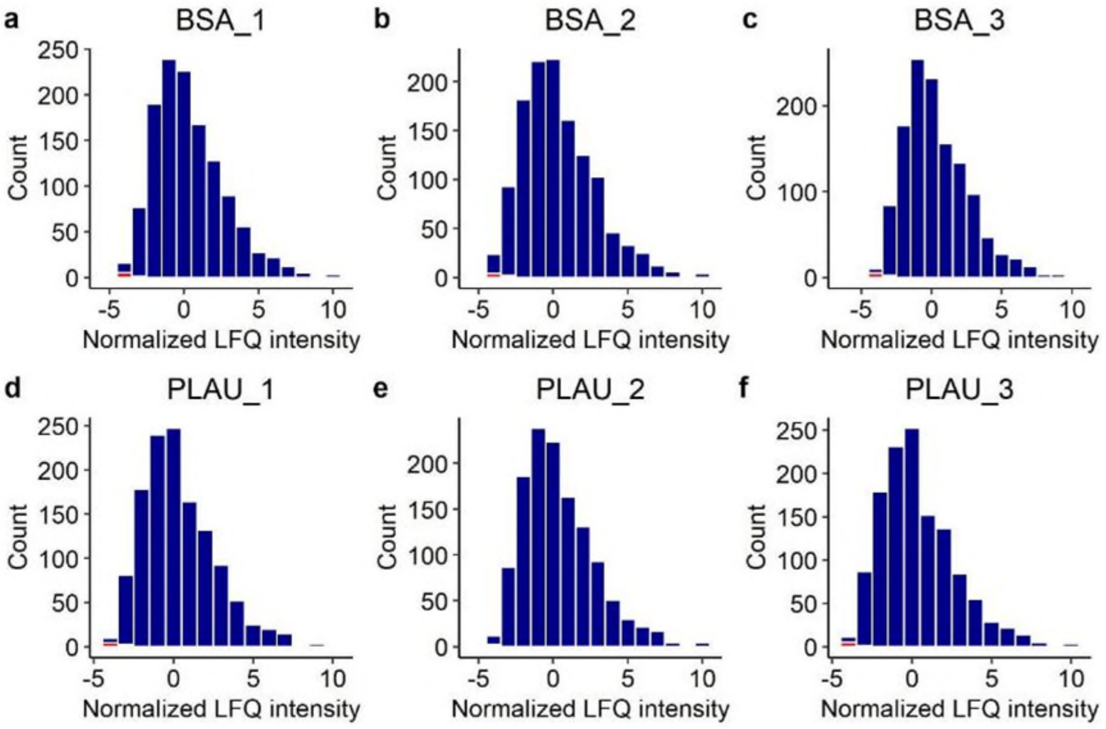

Supplementary Fig. 48 Histograms show the distribution of the normalized LFQ intensities in each sample (marked in blue), and the missing values that were imputed from the normal distribution are marked in red.

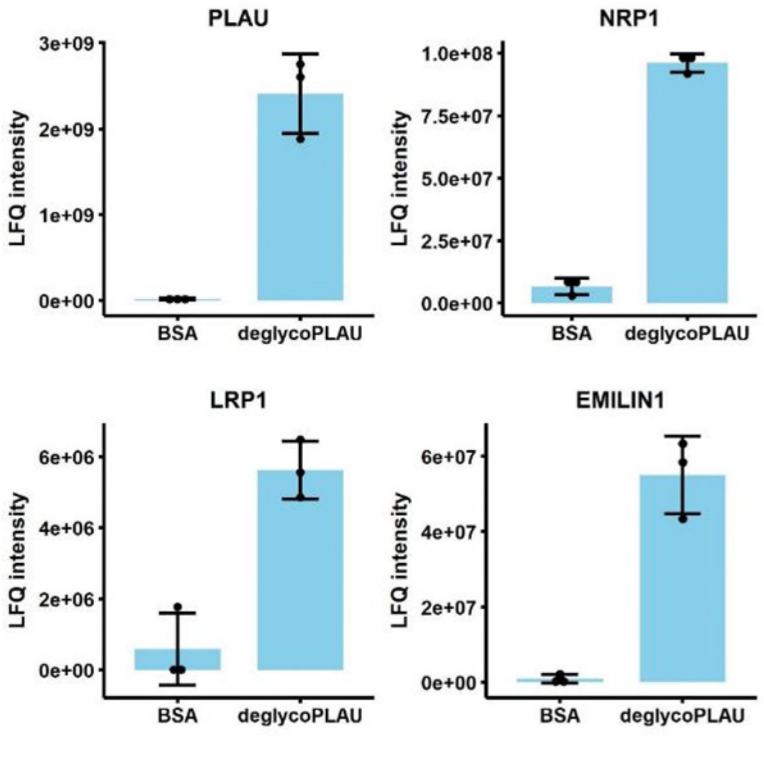

Supplementary Fig. 49 LFQ intensities of the significant ligands and receptors in the volcano plot. Data are presented as mean ± SD ($n = 3$ biological replicates).

Quality control of the MS data in Supplementary Fig. 7

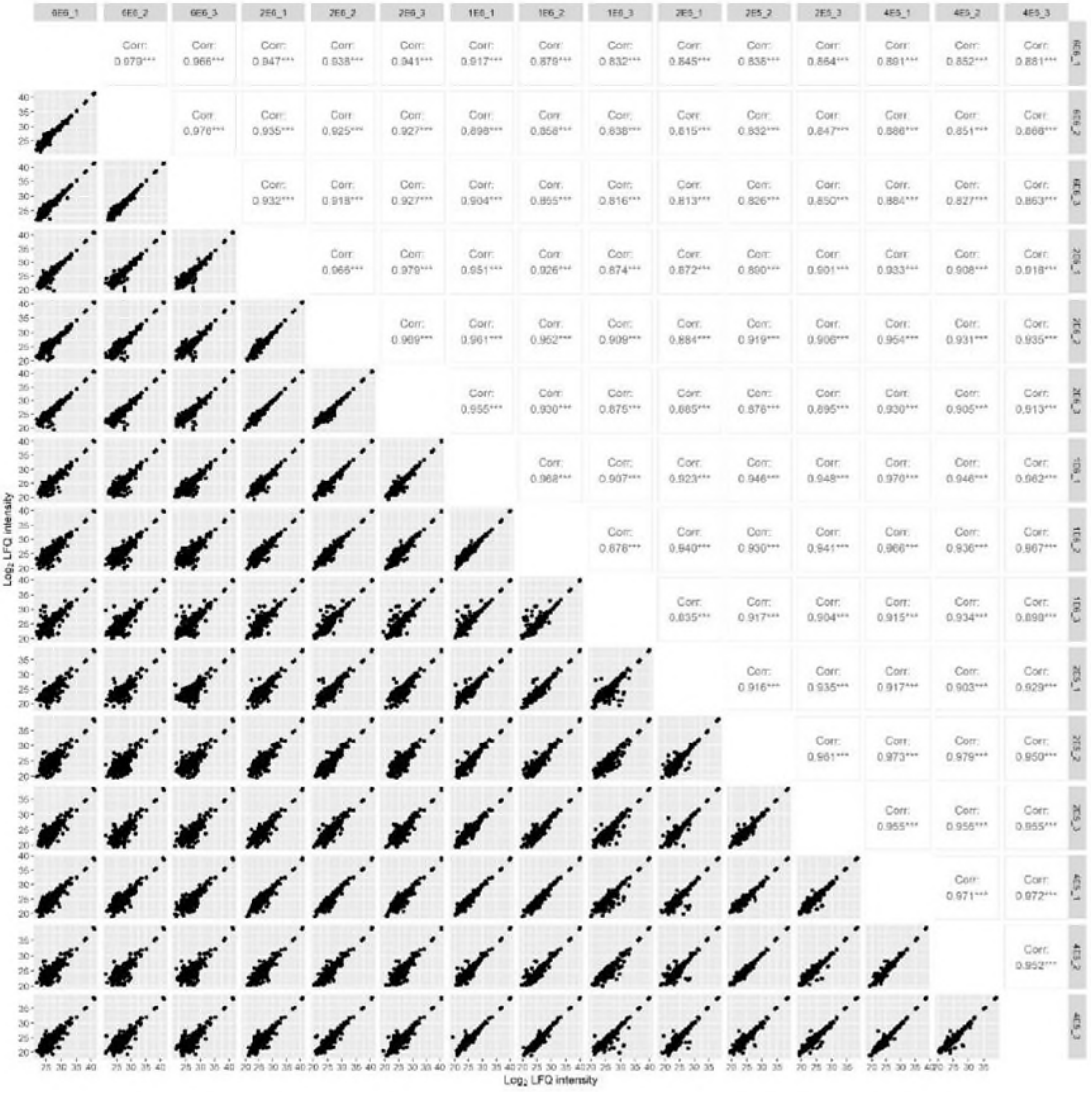

Supplementary Fig. 50 Pairwise Pearson correlation of the Log_2 transformed LFQ intensity before normalization and imputation.

Quality control of the MS data in Supplementary Fig. 8a

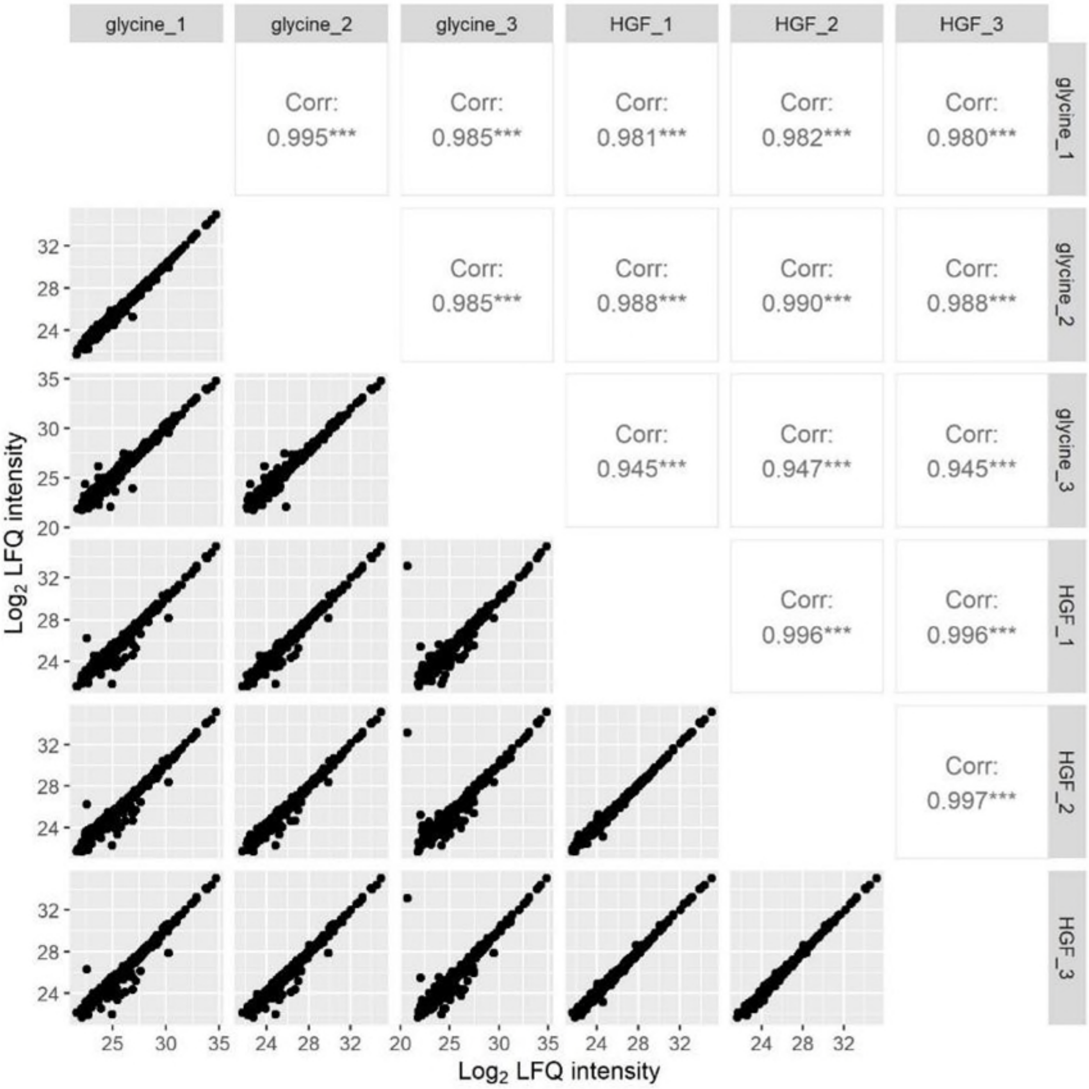

Supplementary Fig. 51 Pairwise Pearson correlation of the Log_2 transformed LFQ intensity before normalization and imputation.

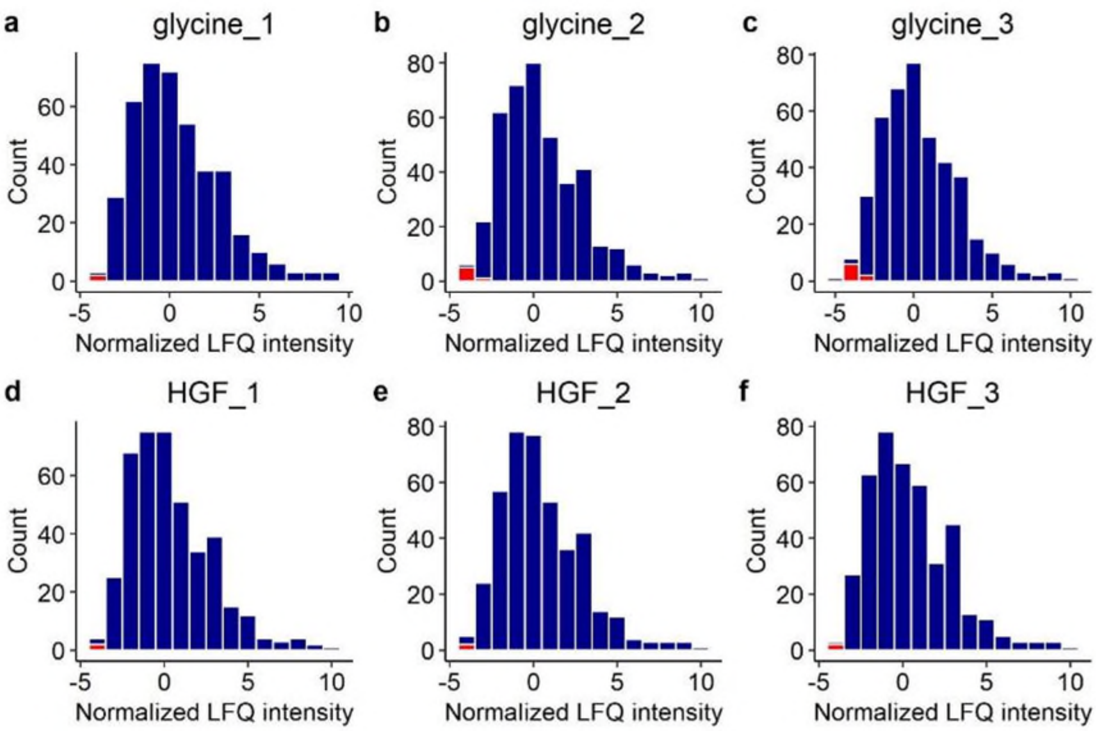

Supplementary Fig. 52 Histograms show the distribution of the normalized LFQ intensities in each sample (marked in blue), and the missing values that were imputed from the normal distribution are marked in red.

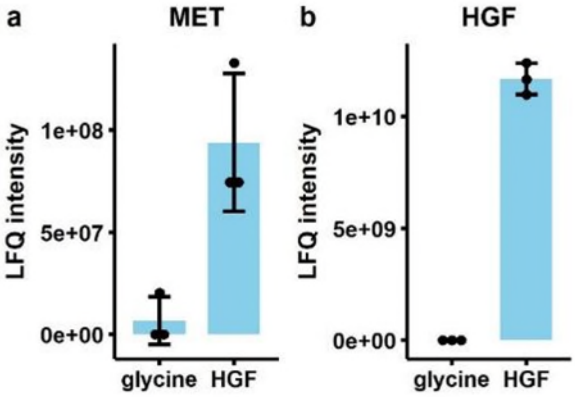

Supplementary Fig. 53 LFQ intensities of the significant receptors identified in the volcano plot. Data are presented as mean ± SD ($n = 3$ biological replicates).

Quality control of the MS data in Supplementary Fig. 8b

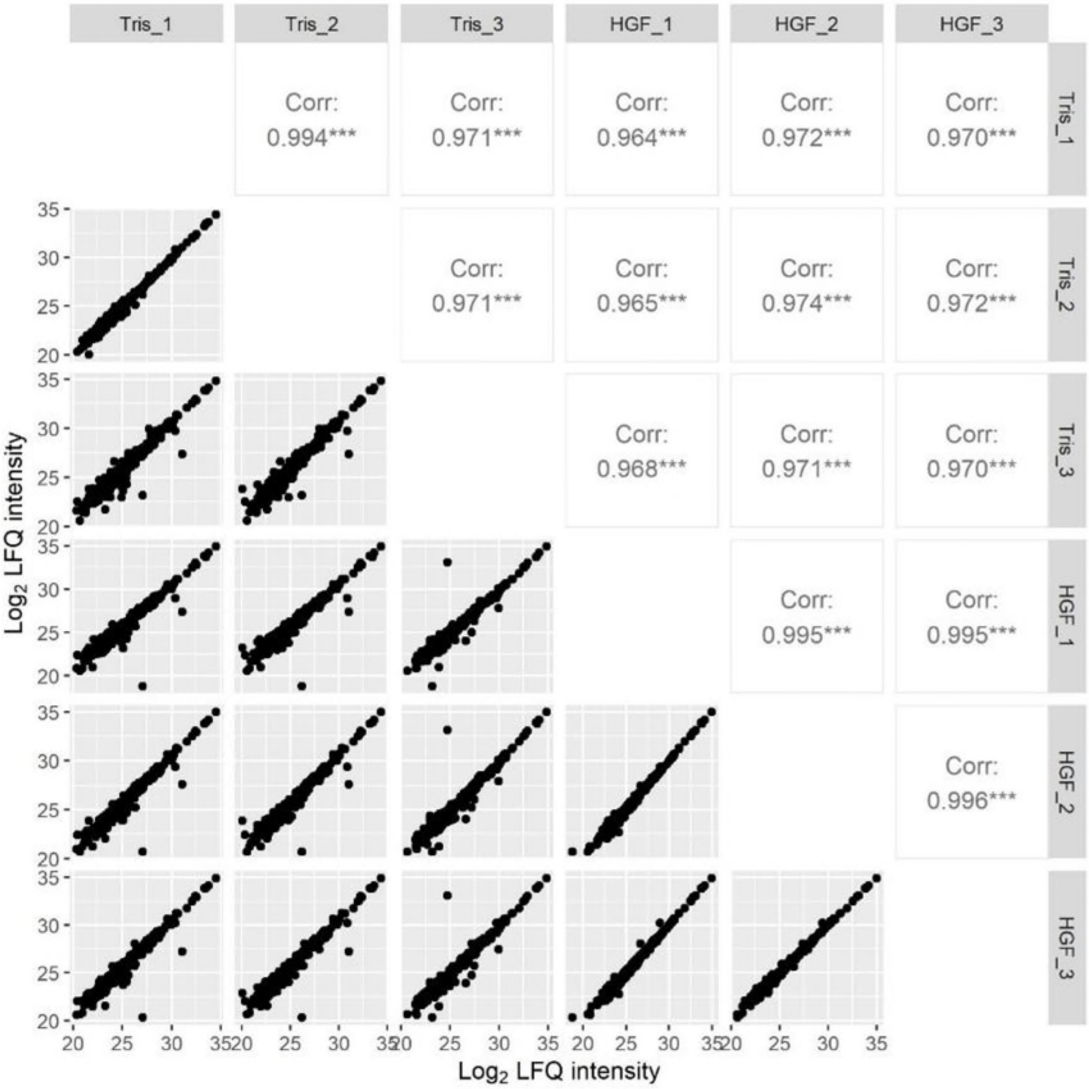

Supplementary Fig. 54 Pairwise Pearson correlation of the Log_2 transformed LFQ intensity before normalization and imputation.

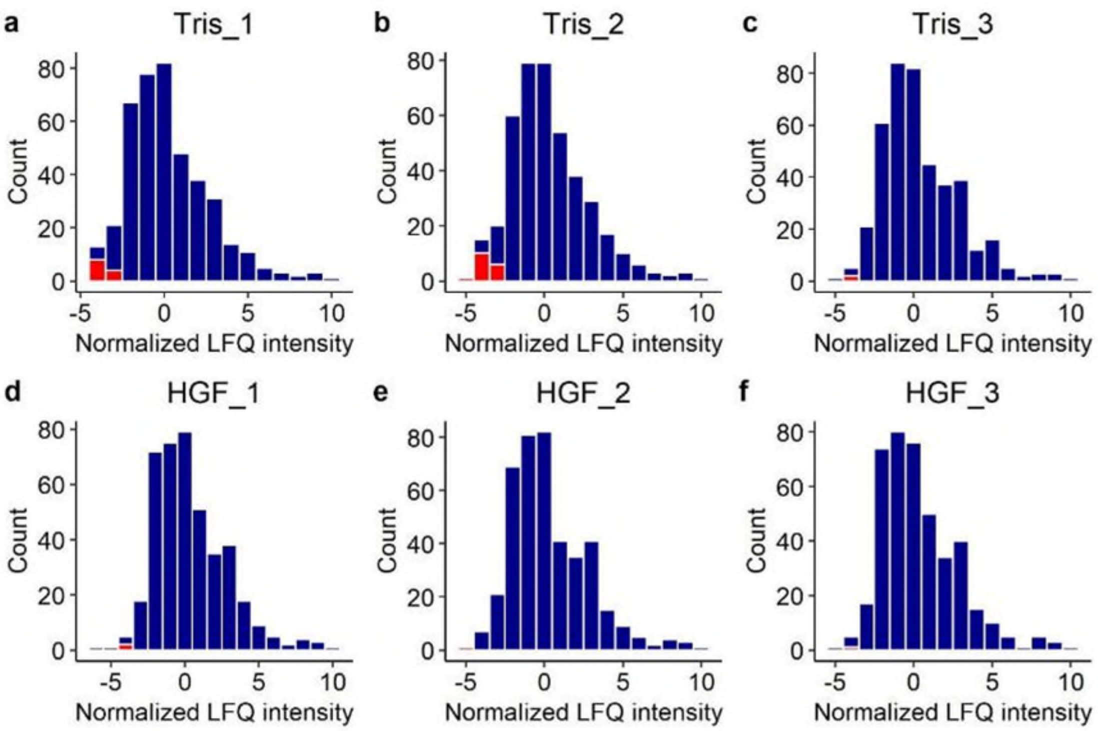

Supplementary Fig. 55 Histograms show the distribution of the normalized LFQ intensities in each sample (marked in blue), and the missing values that were imputed from the normal distribution are marked in red.

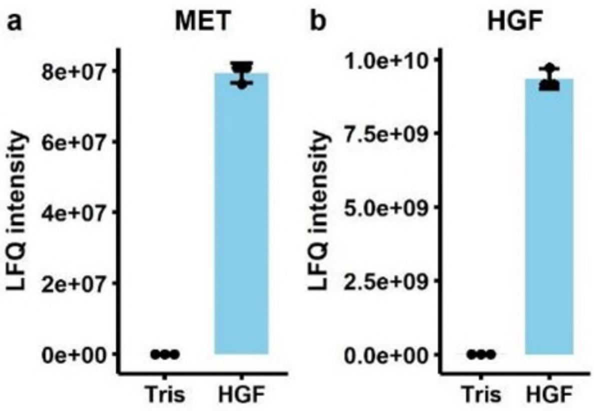

Supplementary Fig. 56 LFQ intensities of the significant ligand and receptor in the volcano plot. Data are presented as mean ± SD ($n = 3$ biological replicates).

Quality control of the MS data in Supplementary Fig. 8c

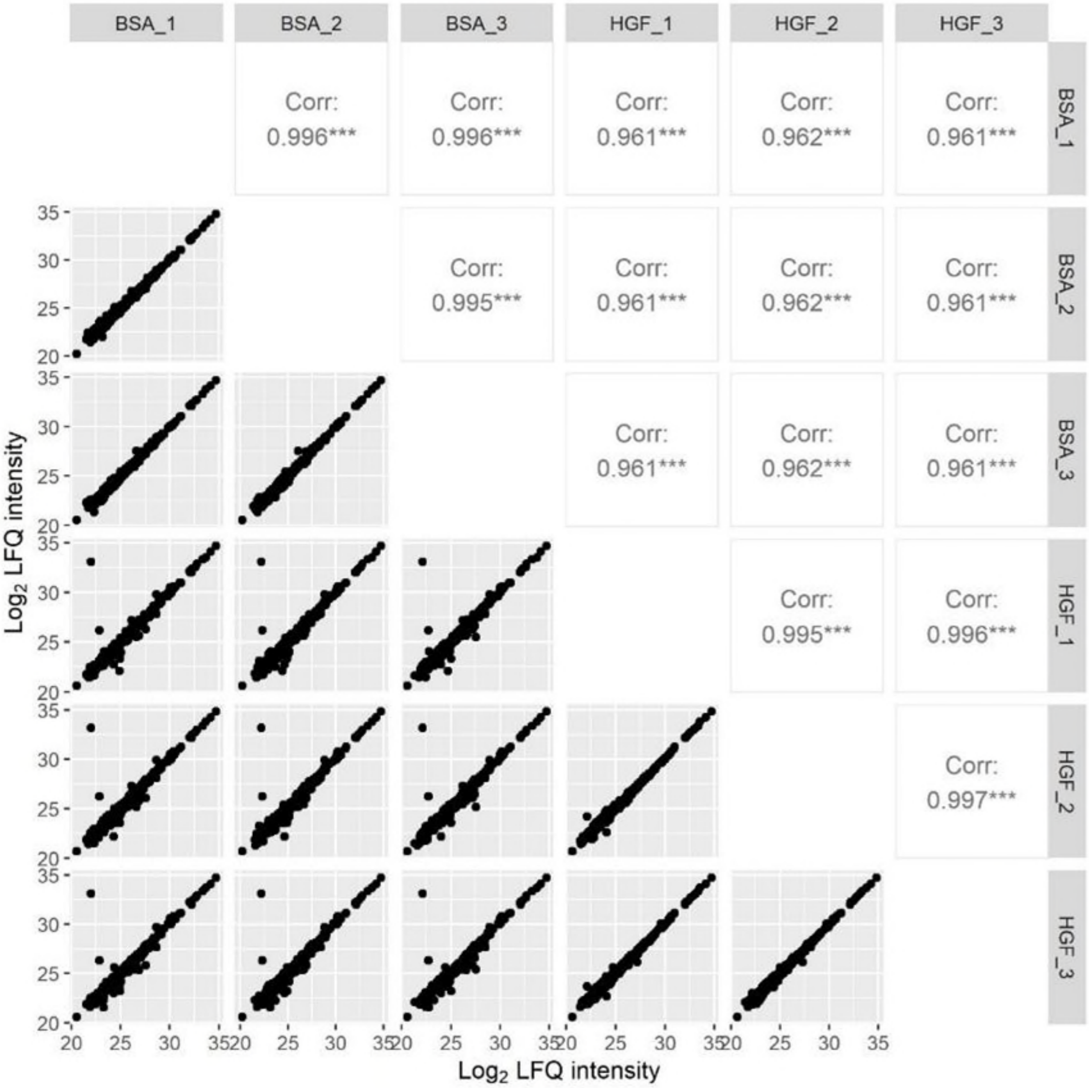

Supplementary Fig. 57 Pairwise Pearson correlation of the Log_2 transformed LFQ intensity before normalization and imputation.

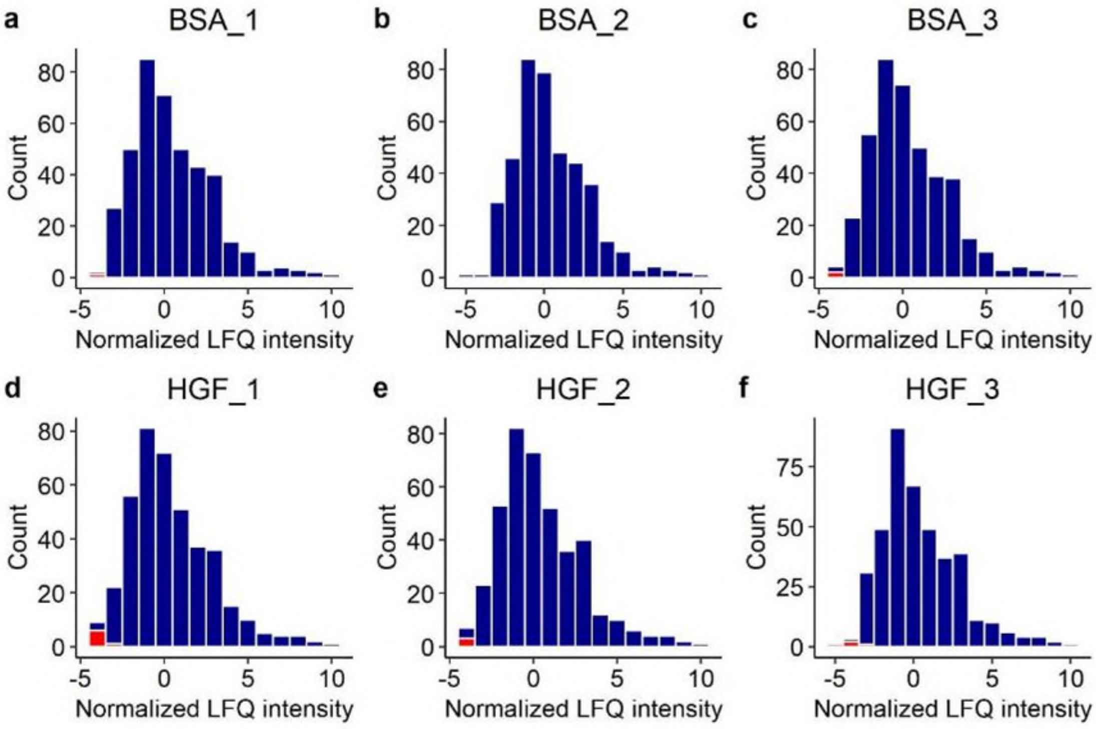

Supplementary Fig. 58 Histograms show the distribution of the normalized LFQ intensities in each sample (marked in blue), and the missing values that were imputed from the normal distribution are marked in red.

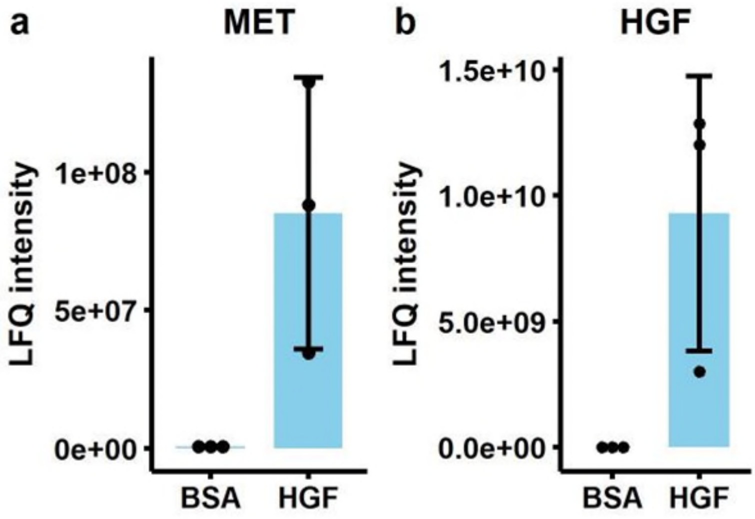

Supplementary Fig. 59 LFQ intensities of the significant ligand and receptor in the volcano plot. Data are presented as mean ± SD ($n = 3$ biological replicates).

Quality control of the MS data in Supplementary Fig. 8d

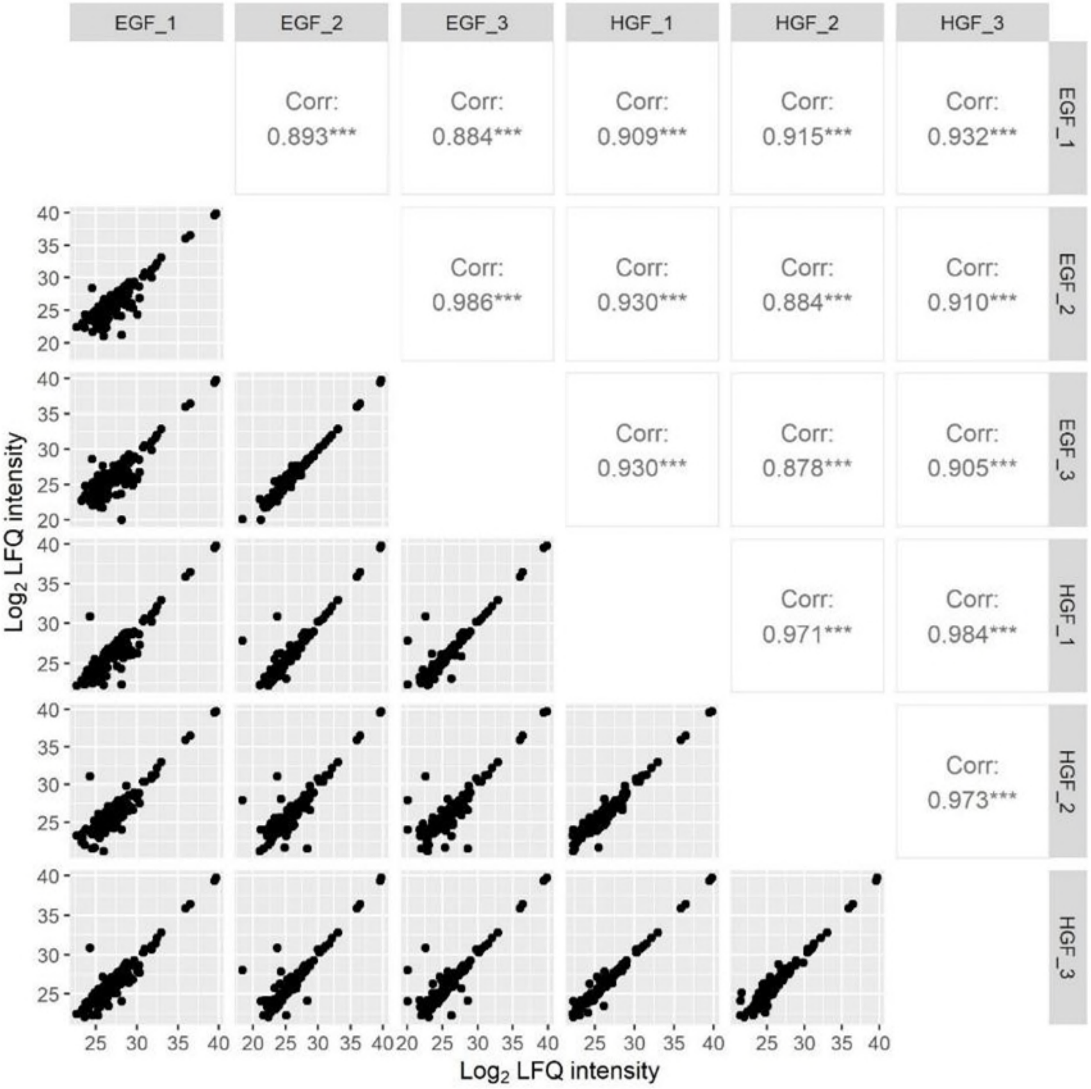

Supplementary Fig. 60 Pairwise Pearson correlation of the Log_2 transformed LFQ intensity before normalization and imputation.

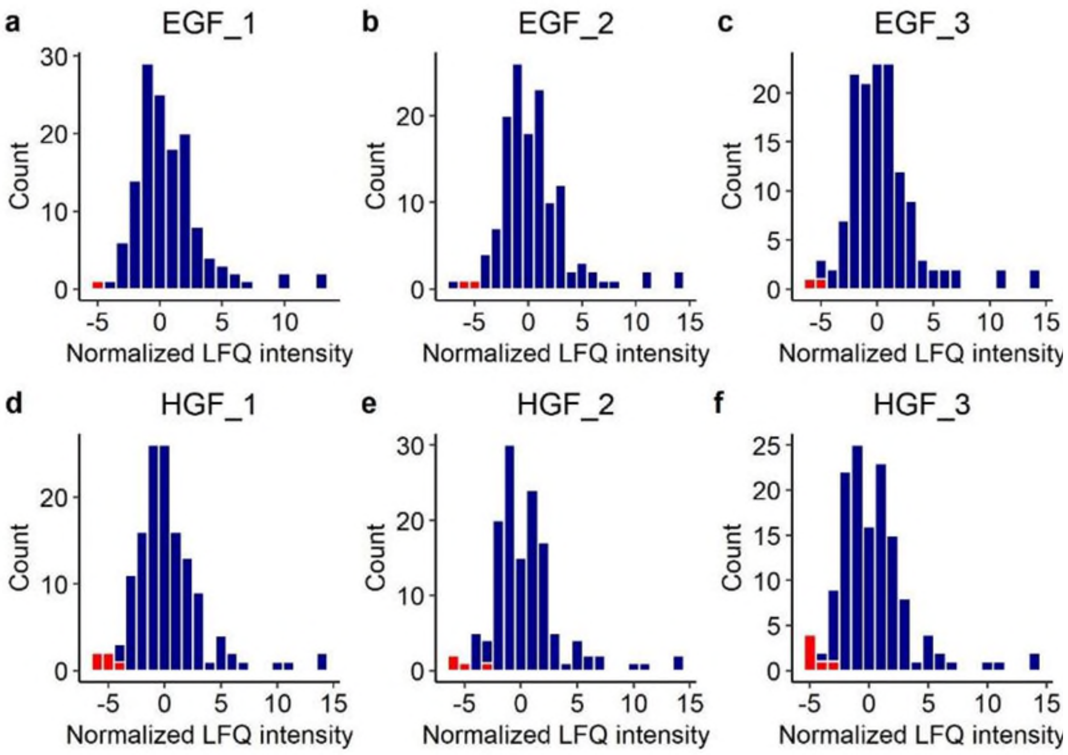

Supplementary Fig. 61 Histograms show the distribution of the normalized LFQ intensities in each sample (marked in blue), and the missing values that were imputed from the normal distribution are marked in red.

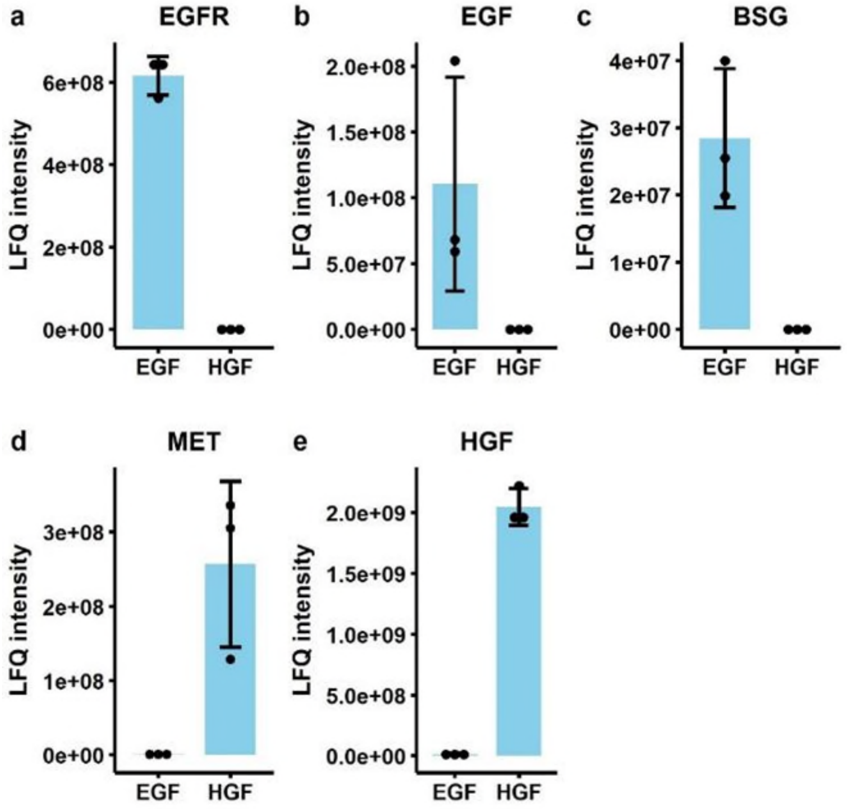

Supplementary Fig. 62 LFQ intensities of the significant ligands and receptors in the volcano plot. Data are

presented as mean ± SD ($n = 3$ biological replicates).

Quality control of the MS data in Supplementary Fig. 8e

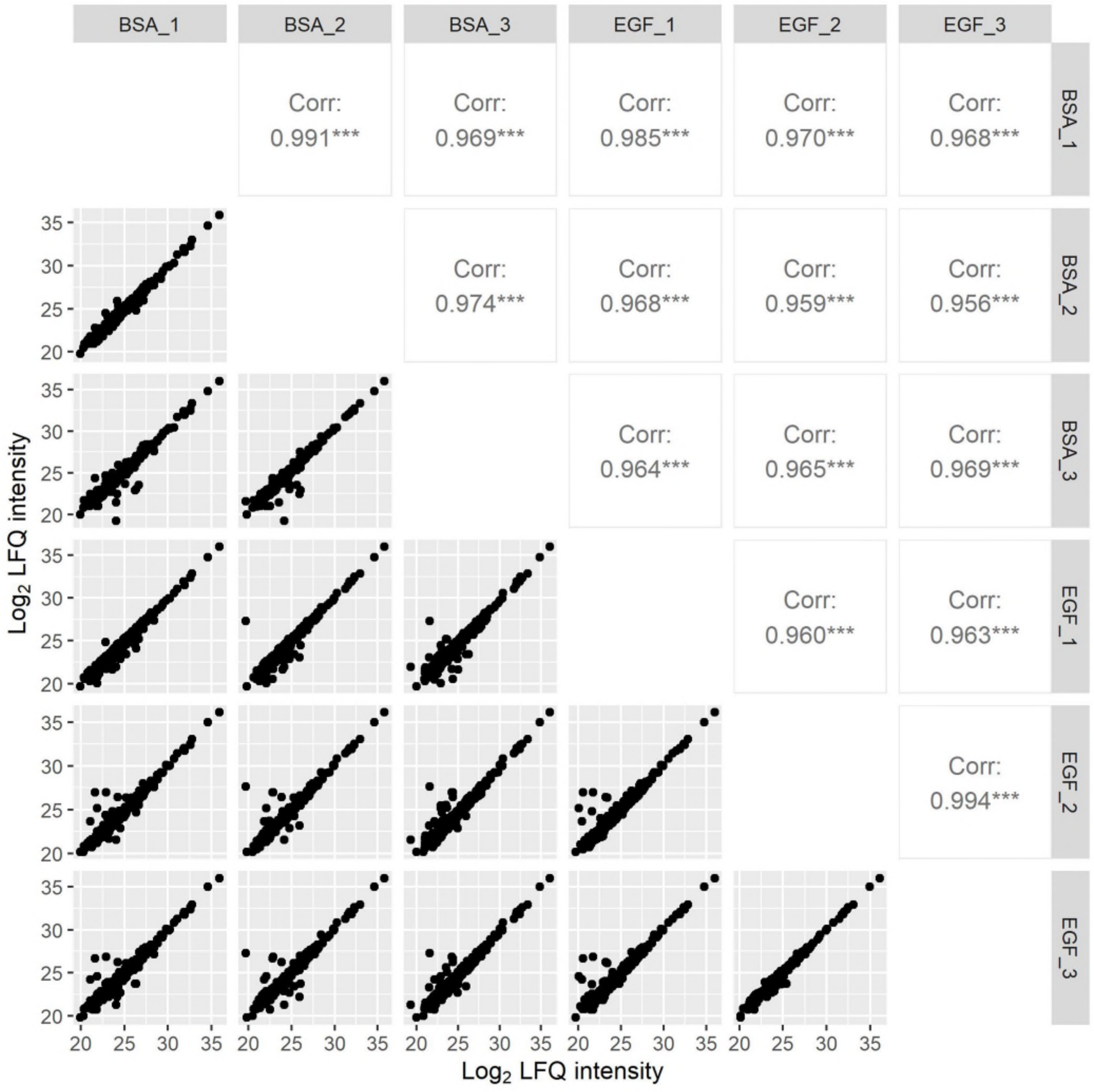

Supplementary Fig. 63 Pairwise Pearson correlation of the Log_2 transformed LFQ intensity before normalization and imputation.

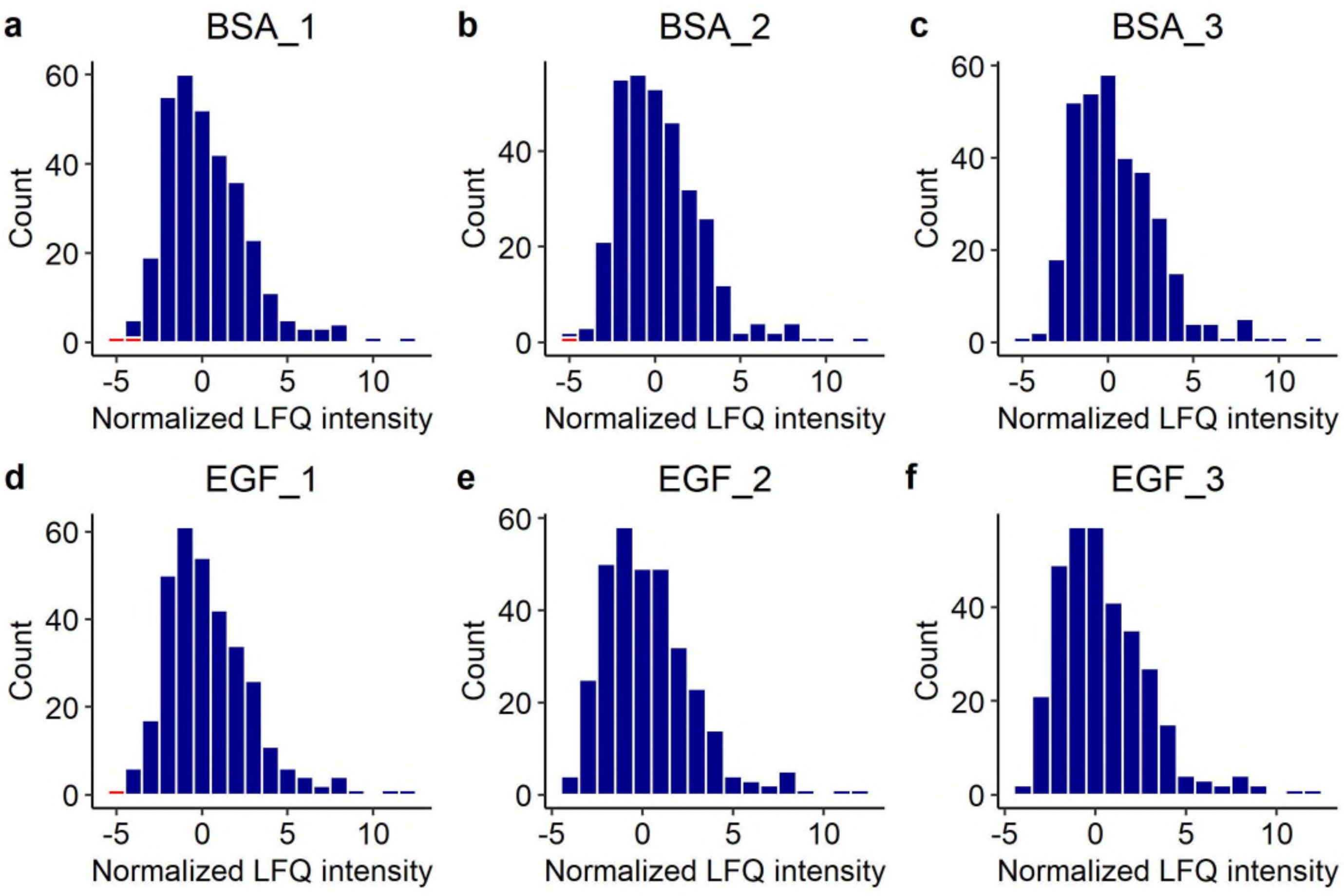

Supplementary Fig. 64 Histograms show the distribution of the normalized LFQ intensities in each sample (marked in blue), and the missing values that were imputed from the normal distribution are marked in red.

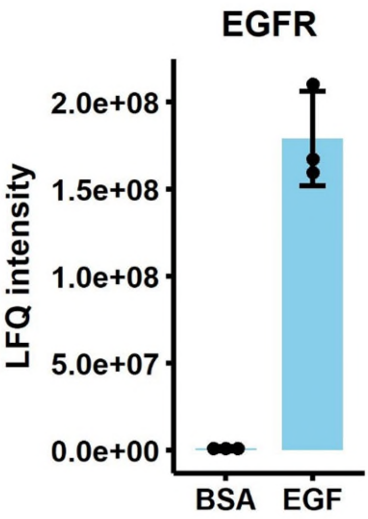

Supplementary Fig. 65 LFQ intensities of the significant receptors in the volcano plot. Data are presented as

mean ± SD ($n = 3$ biological replicates).

Quality control of the MS data in Supplementary Fig. 8f

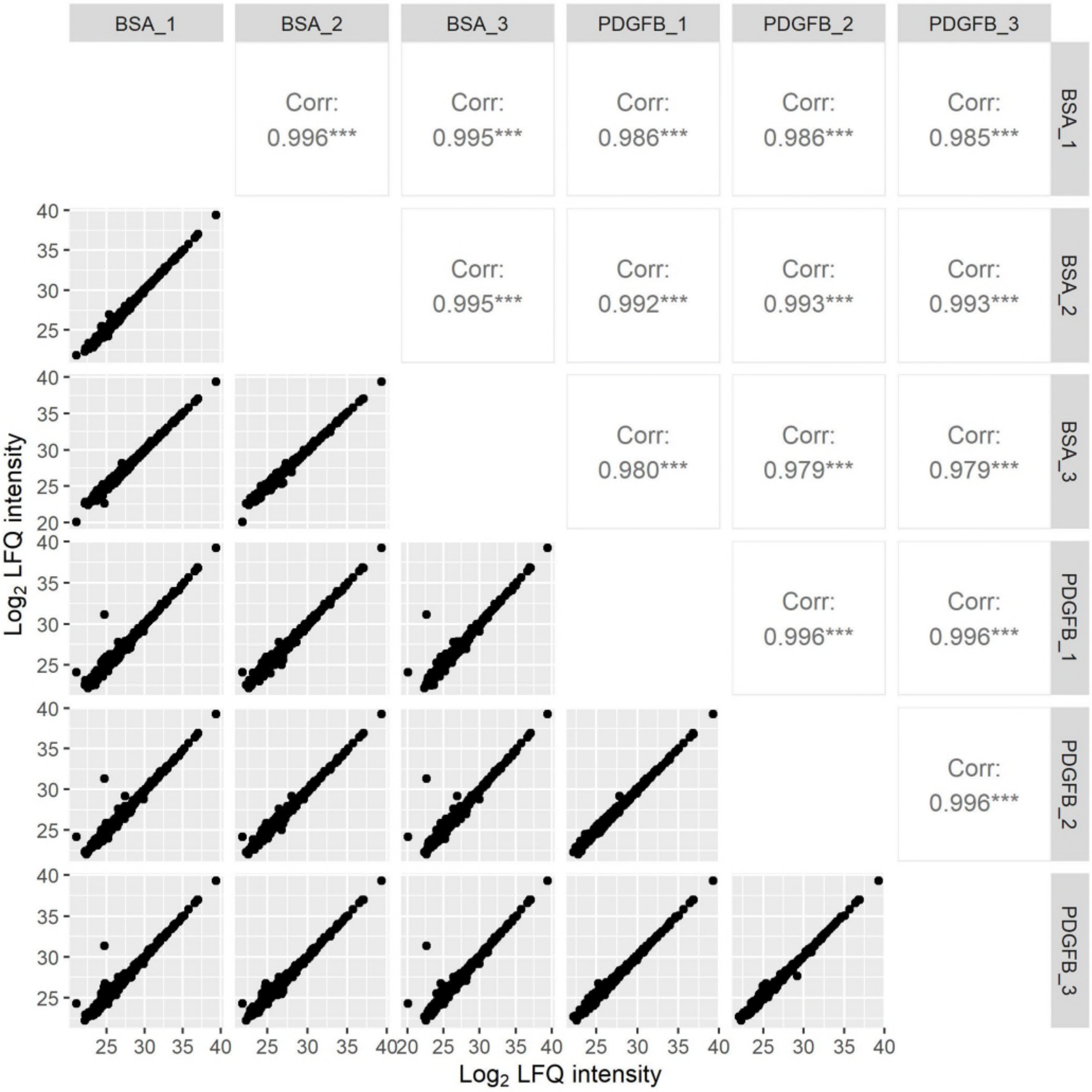

Supplementary Fig. 66 Pairwise Pearson correlation of the Log_2 transformed LFQ intensity before normalization and imputation.

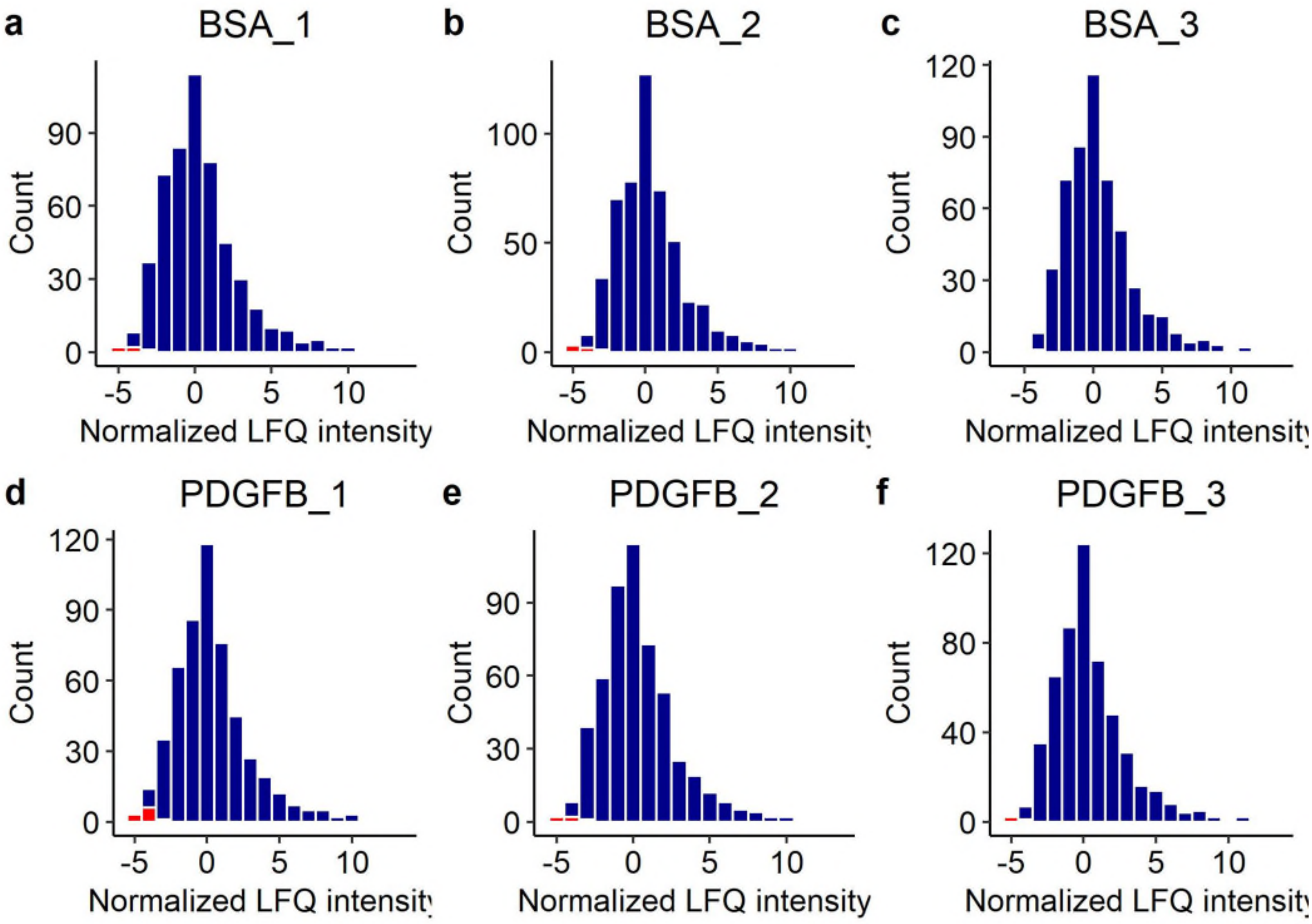

Supplementary Fig. 67 Histograms show the distribution of the normalized LFQ intensities in each sample (marked in blue), and the missing values that were imputed from the normal distribution are marked in red.

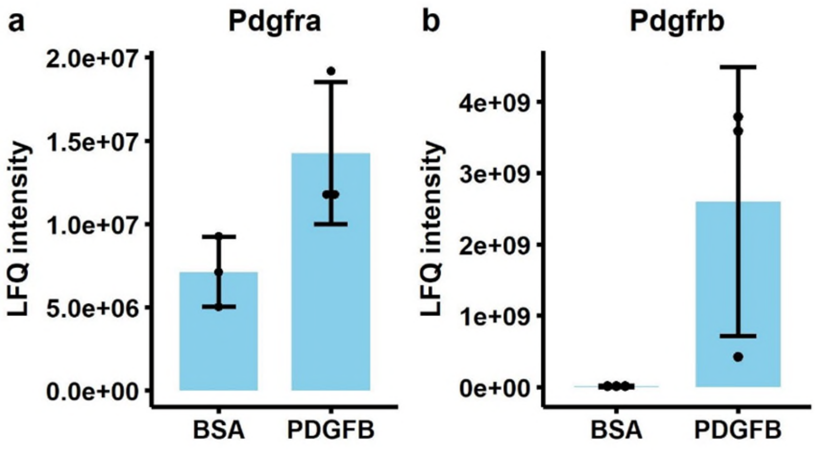

Supplementary Fig. 68 LFQ intensities of the significant receptors in the volcano plot. Data are presented as mean ± SD (n = 3 biological replicates).

Quality control of the MS data in Supplementary Fig. 8g

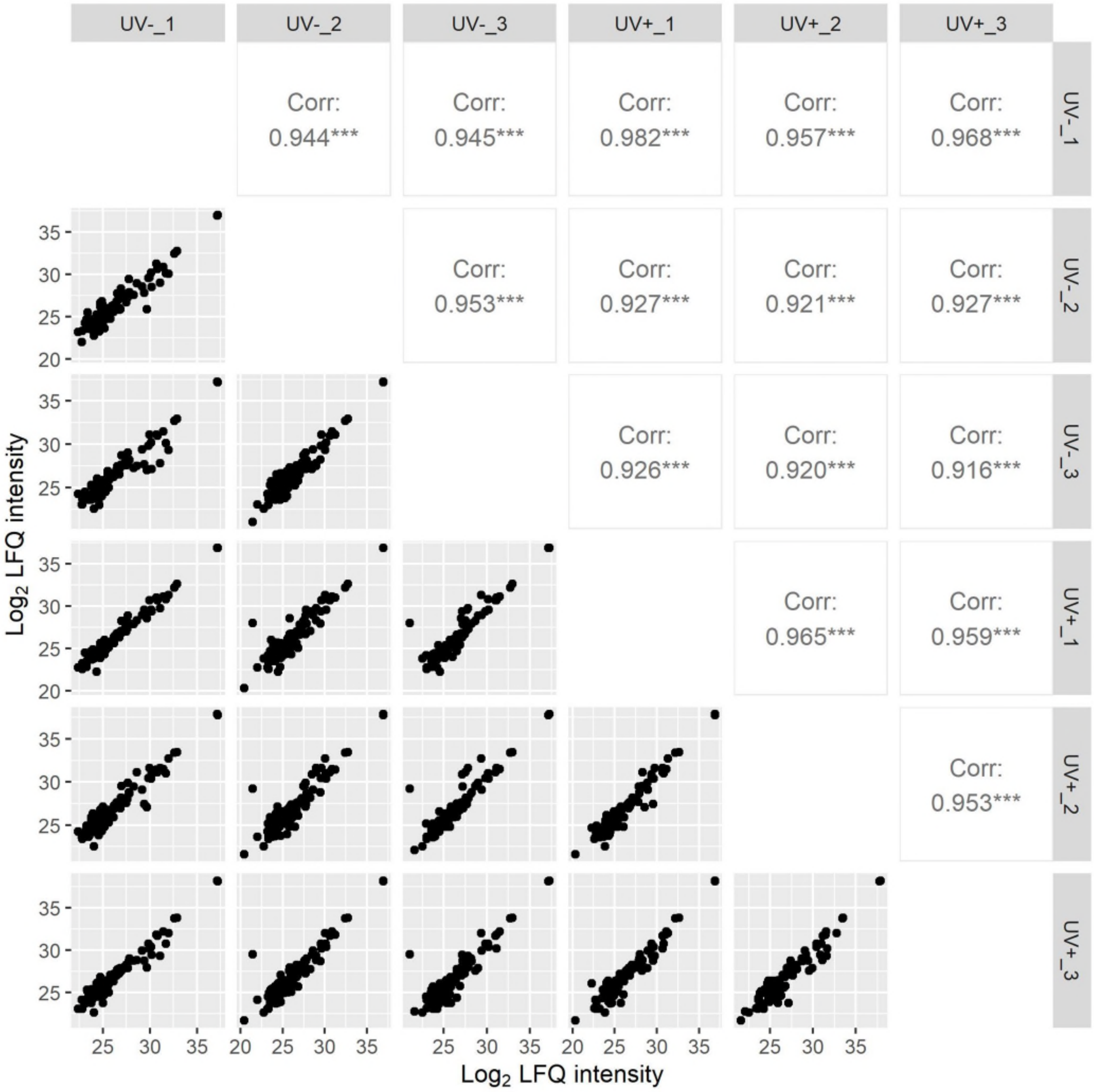

Supplementary Fig. 69 Pairwise Pearson correlation of the Log_2 transformed LFQ intensity before normalization and imputation.

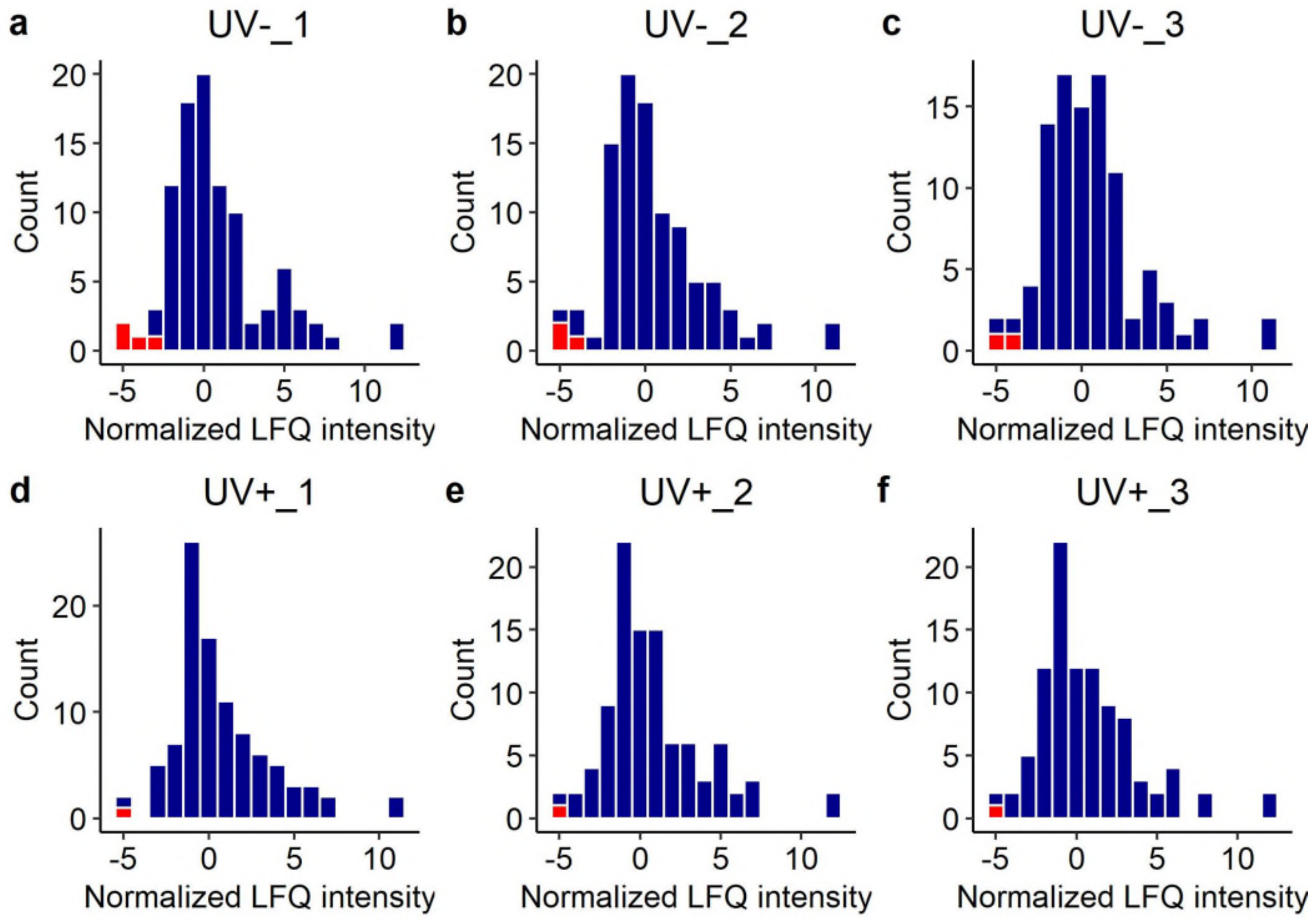

Supplementary Fig. 70 Histograms show the distribution of the normalized LFQ intensities in each sample (marked in blue), and the missing values that were imputed from the normal distribution are marked in red.

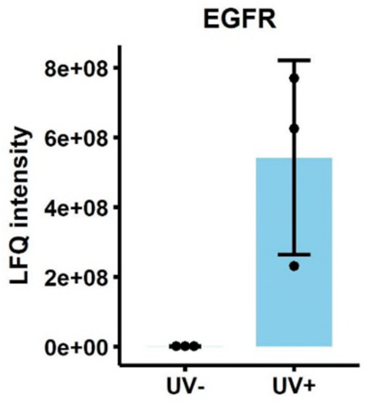

Supplementary Fig. 71 LFQ intensities of the significant receptors in the volcano plot. Data are presented as mean ± SD ($n = 3$ biological replicates).

Quality control of the MS data in Supplementary Fig. 8h

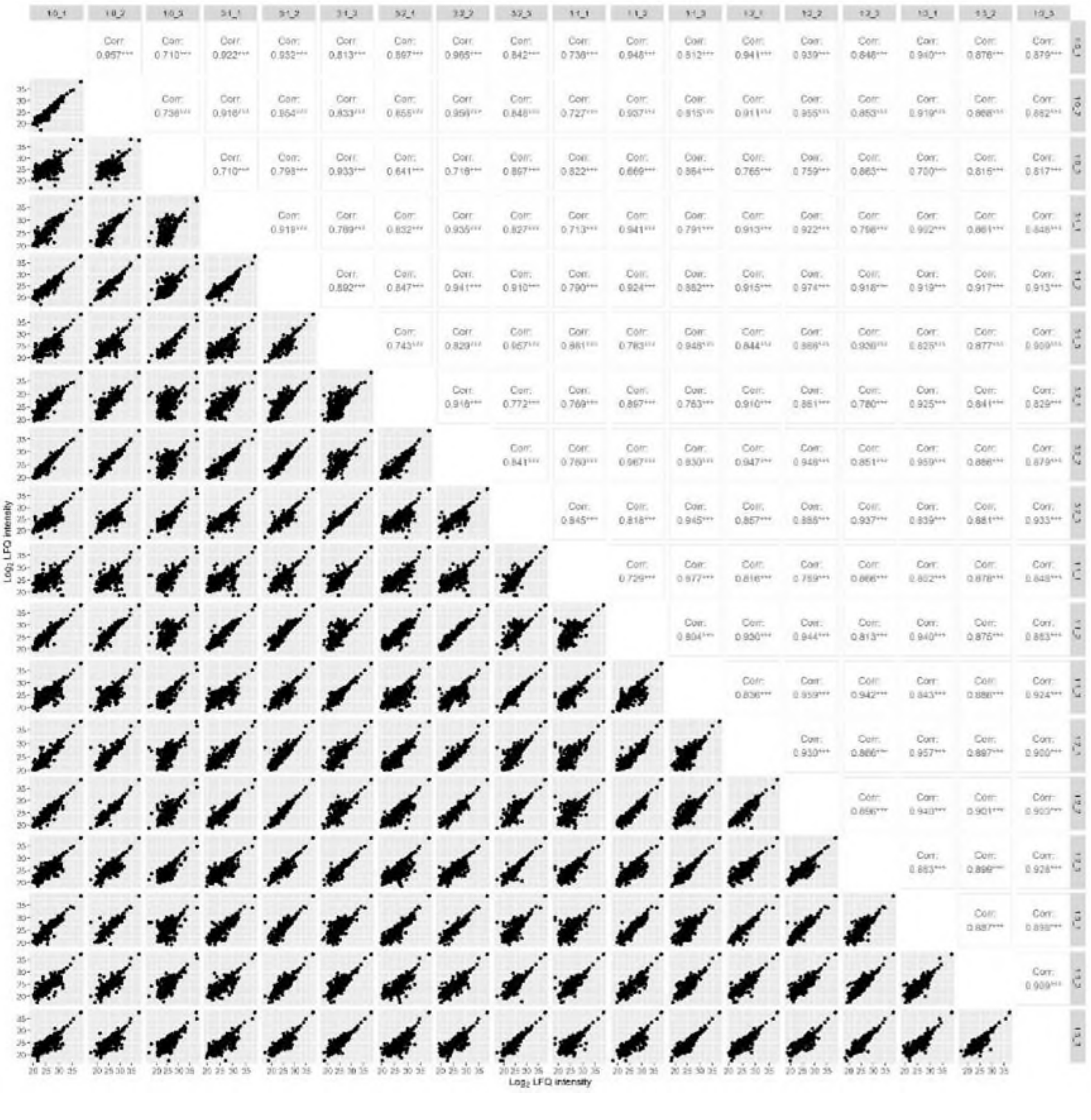

Supplementary Fig. 72 Pairwise Pearson correlation of the Log_2 transformed LFQ intensity before normalization and imputation.

Quality control of the MS data in Supplementary Fig. 8i

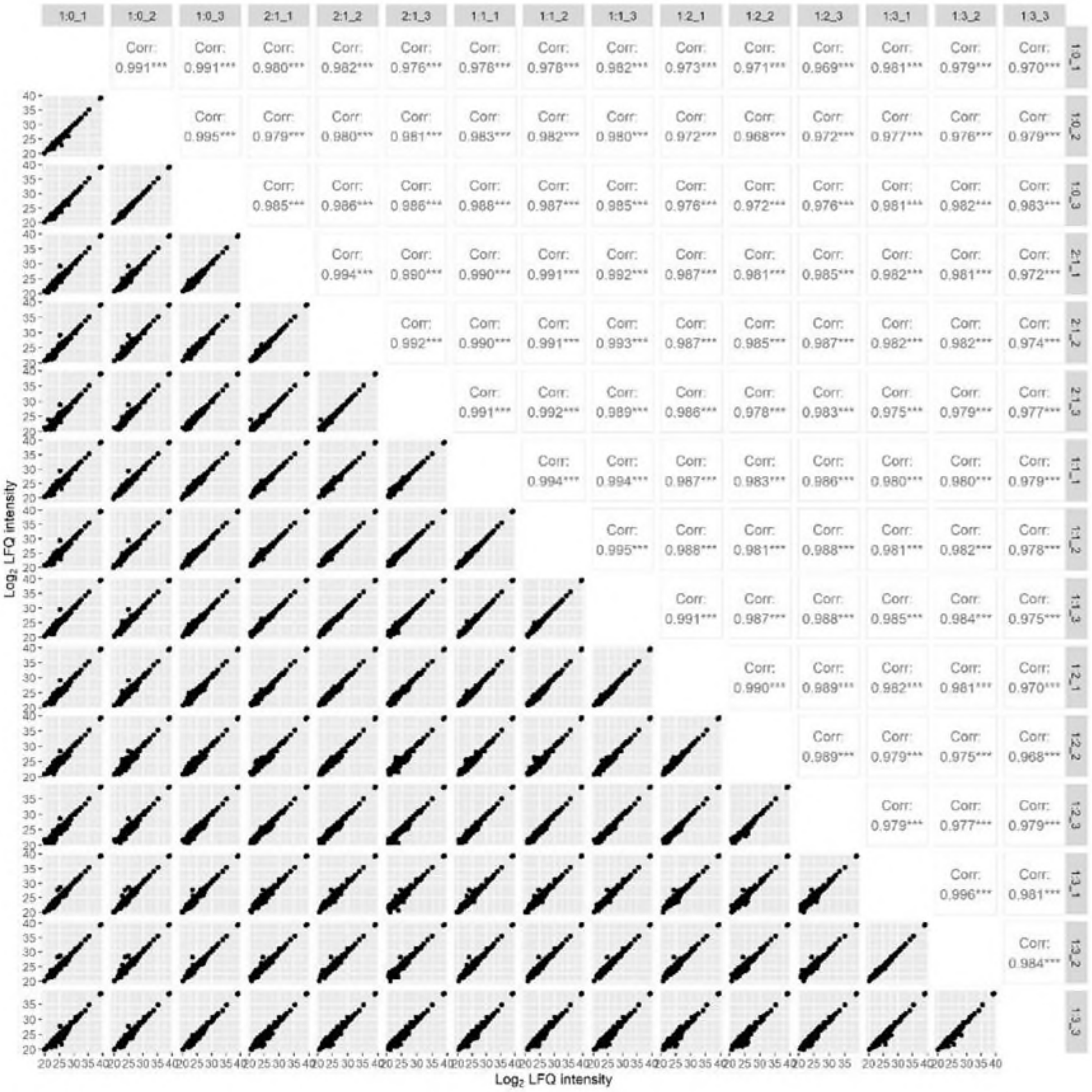

Supplementary Fig. 73 Pairwise Pearson correlation of the Log_2 transformed LFQ intensity before normalization and imputation.

Quality control of the MS data in Supplementary Fig. 8j, k

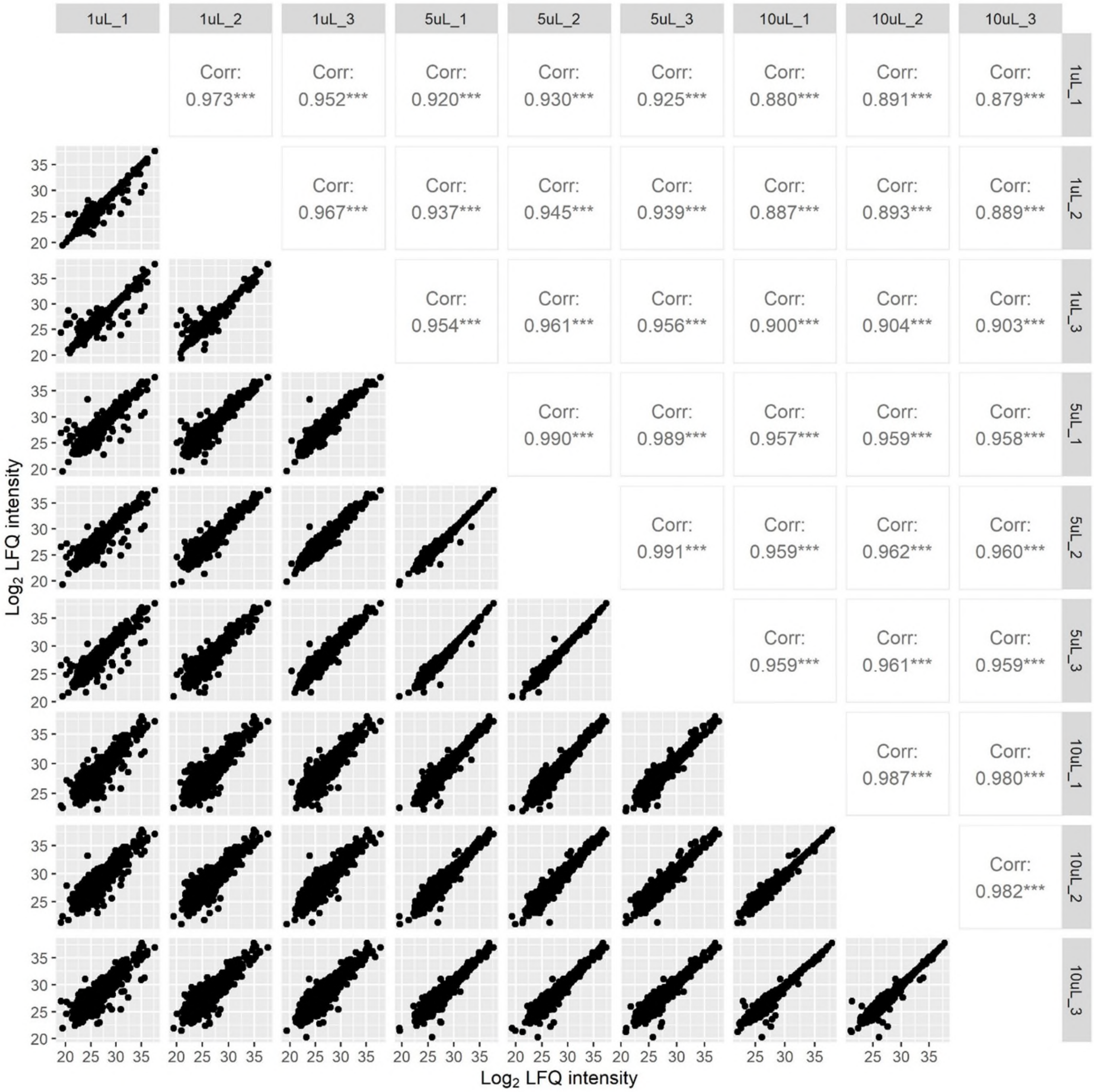

Supplementary Fig. 74 Pairwise Pearson correlation of the Log_2 transformed LFQ intensity before normalization and imputation.

Quality control of the MS data in Supplementary Fig. 9

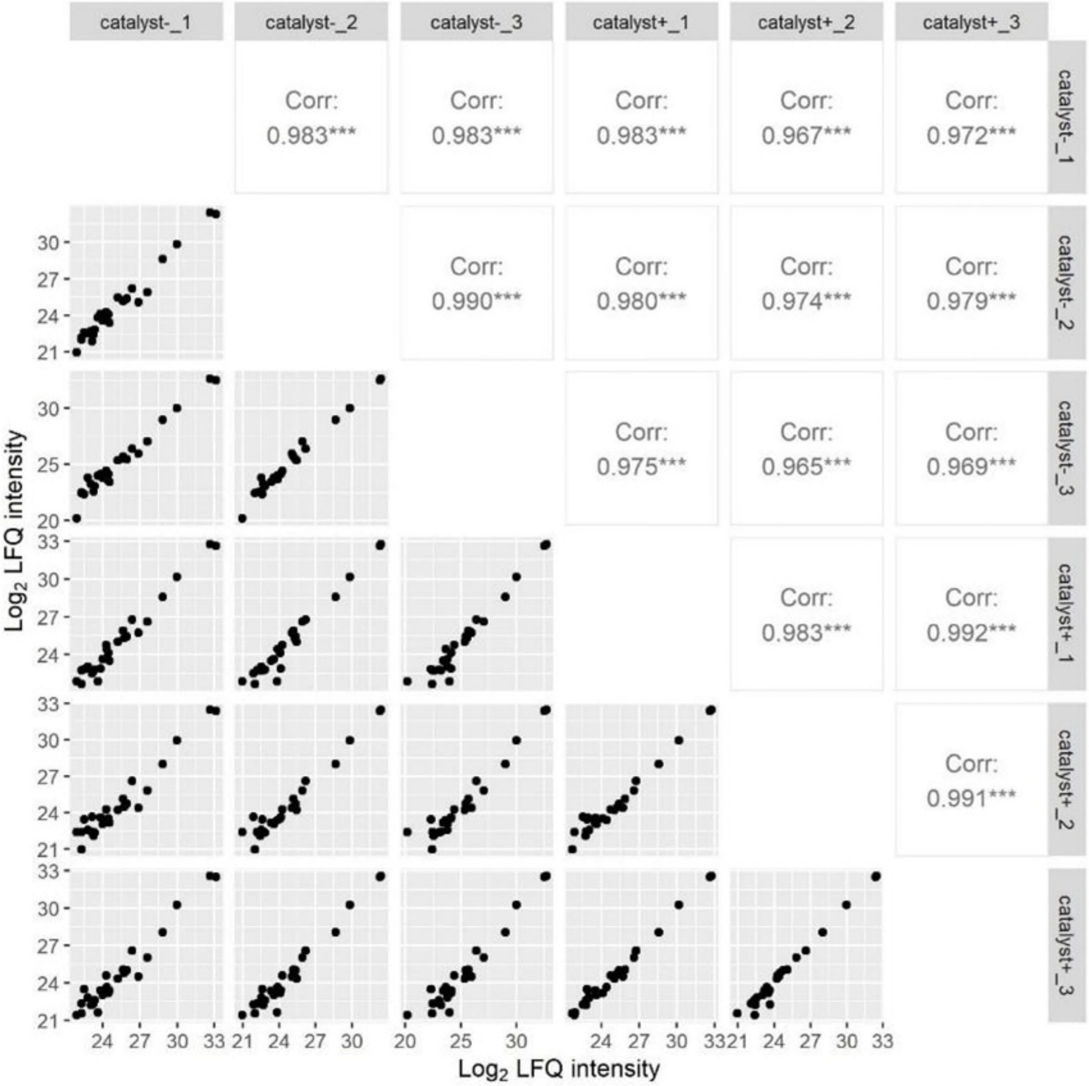

Supplementary Fig. 75 Pairwise Pearson correlation of the Log_2 transformed LFQ intensity before normalization and imputation.

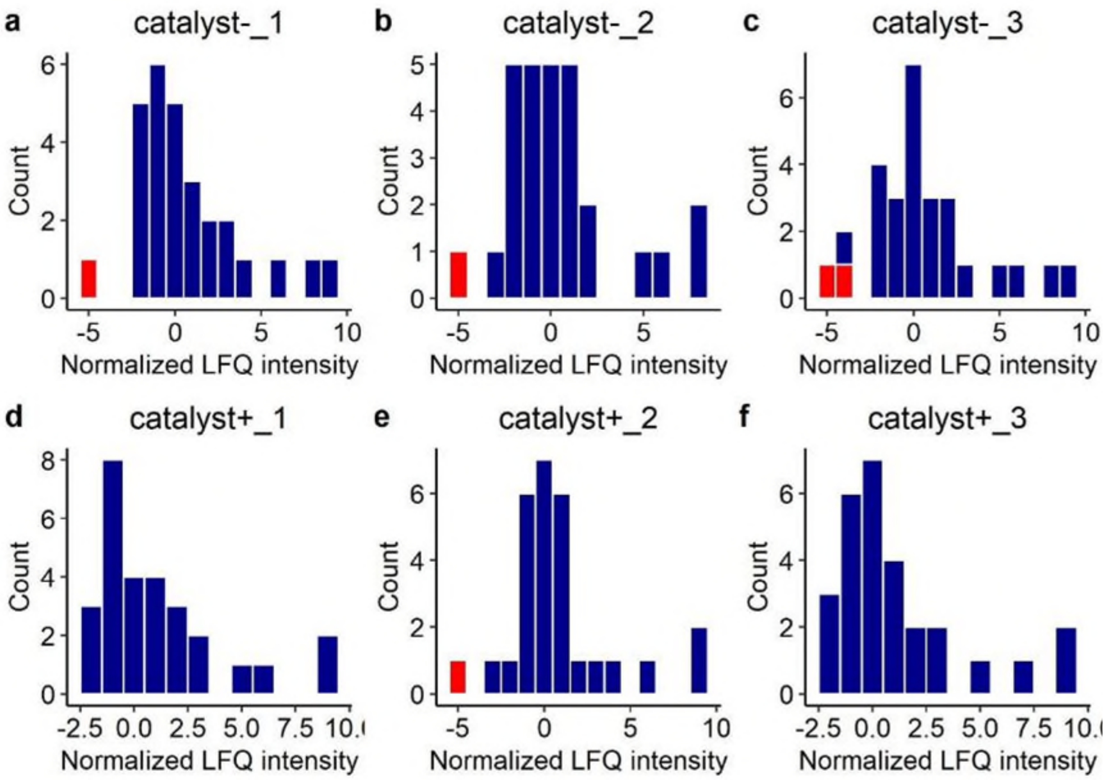

Supplementary Fig. 76 Histograms show the distribution of the normalized LFQ intensities in each sample (marked in blue), and the missing values that were imputed from the normal distribution are marked in red.

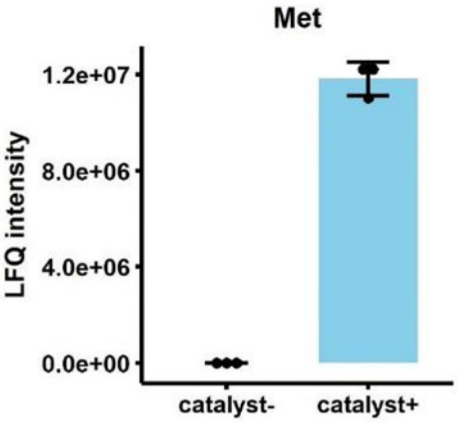

Supplementary Fig. 77 LFQ intensities of the significant receptors identified in the volcano plot. Data are presented as mean ± SD ($n = 3$ biological replicates).

Quality control of the MS data in Supplementary Fig. 10a

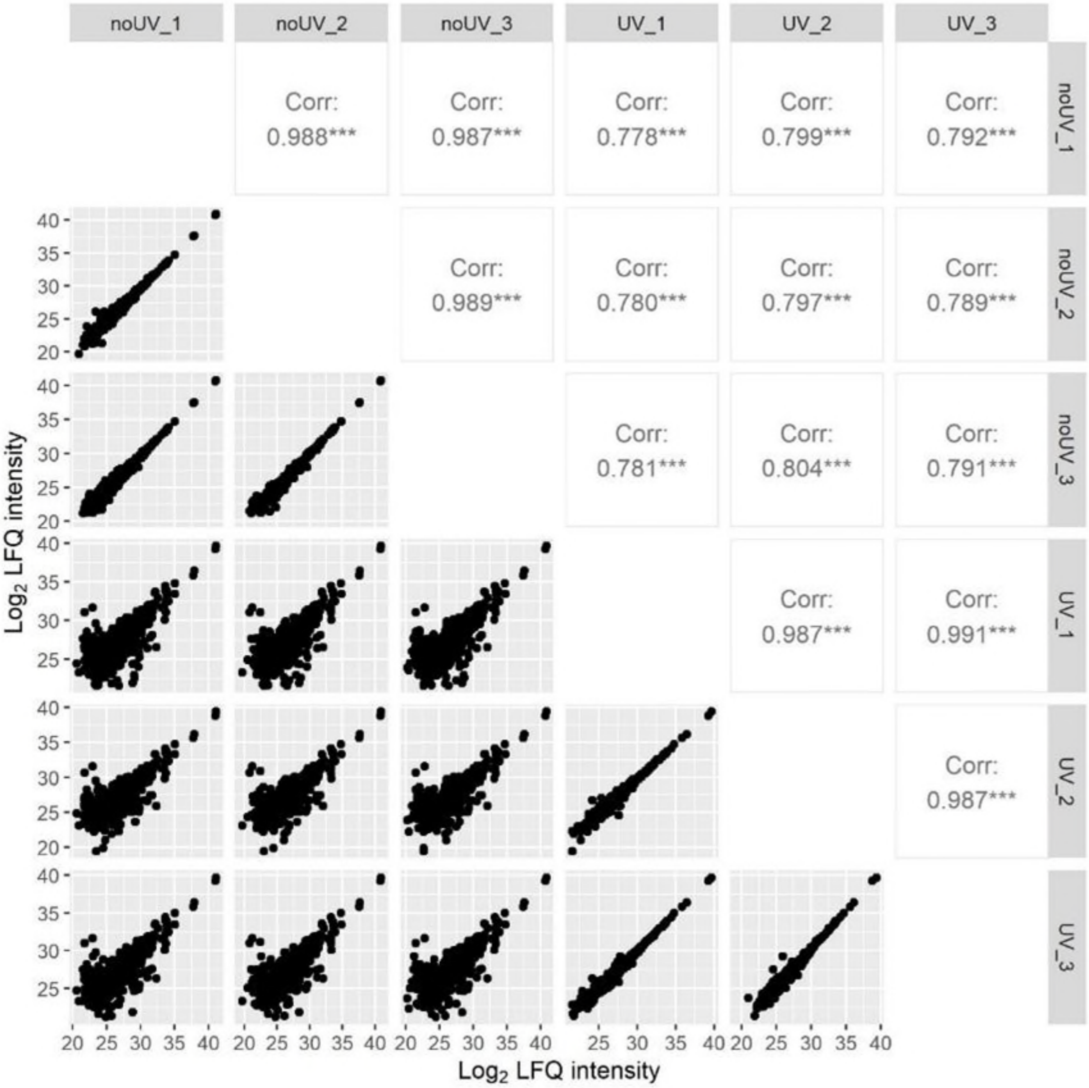

Supplementary Fig. 78 Pairwise Pearson correlation of the Log_2 transformed LFQ intensity before normalization and imputation.

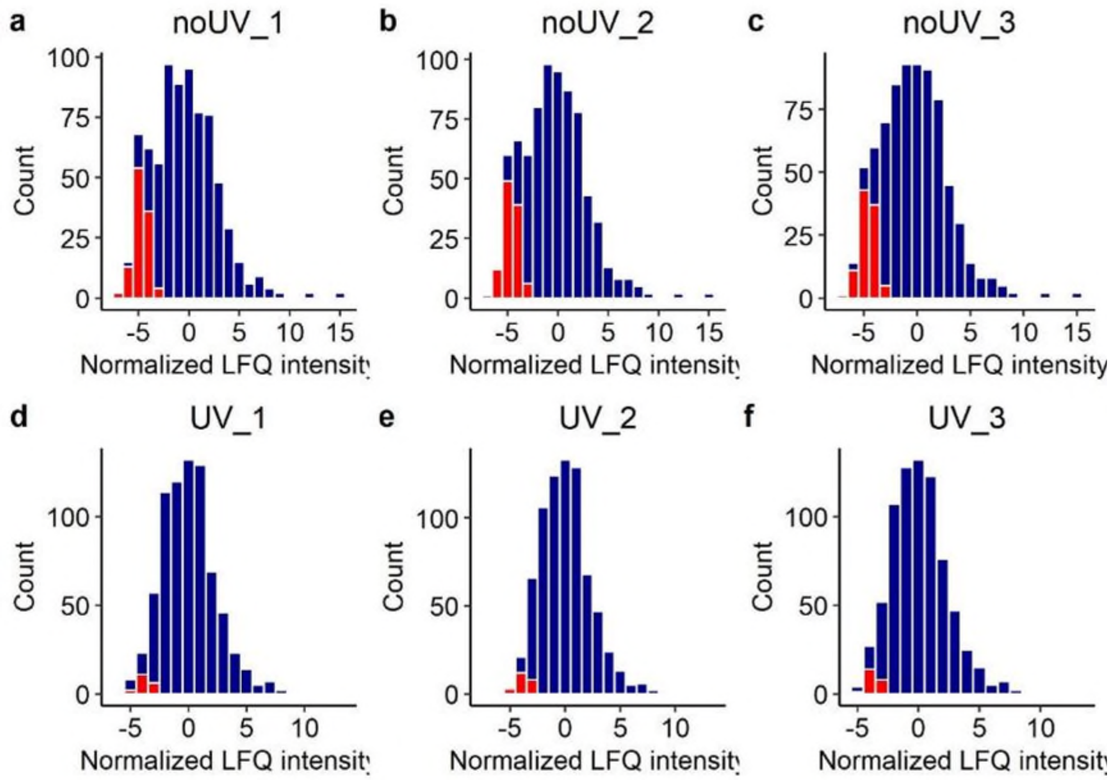

Supplementary Fig. 79 Histograms show the distribution of the normalized LFQ intensities in each sample (marked in blue), and the missing values that were imputed from the normal distribution are marked in red.

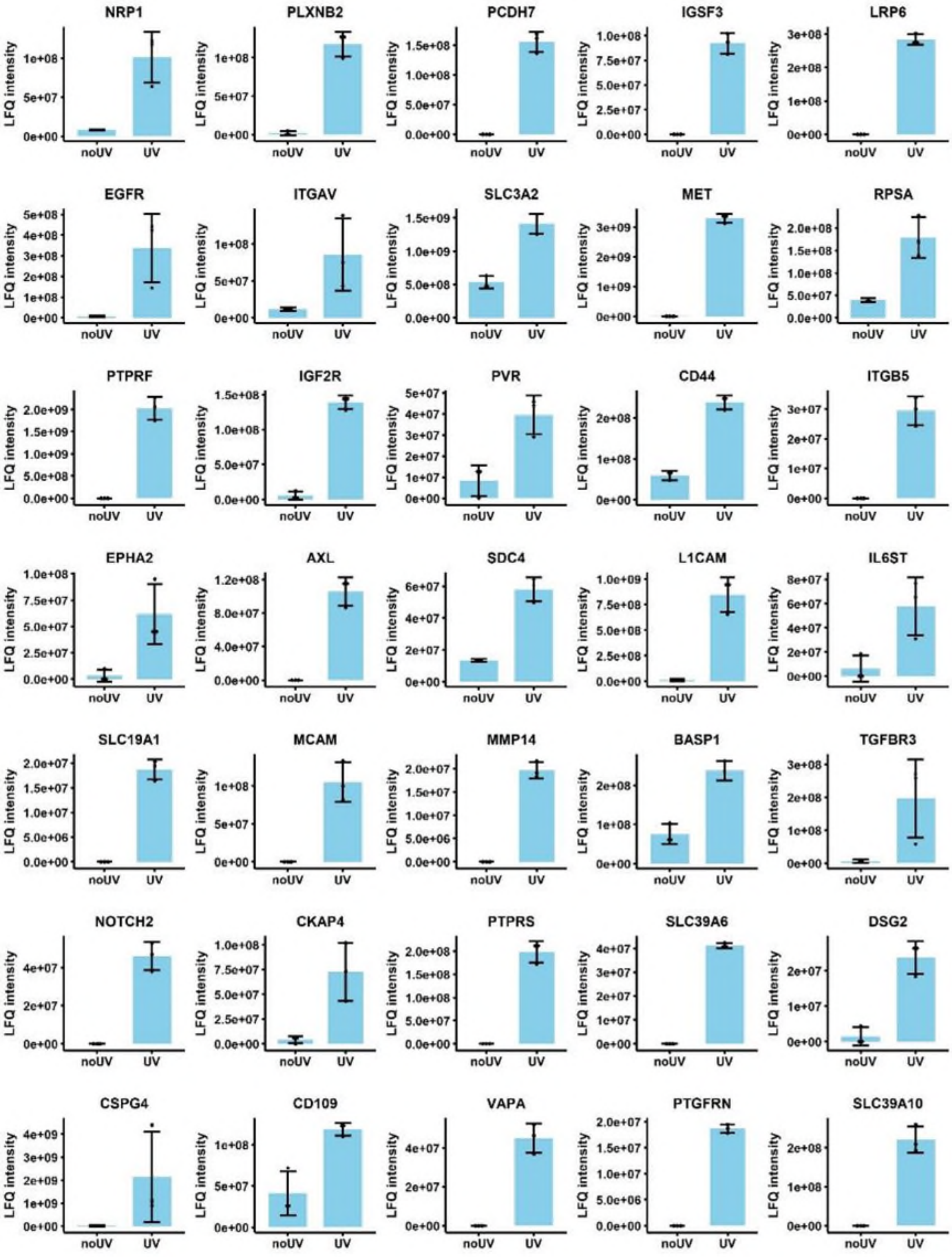

Supplementary Fig. 80 LFQ intensities of the significant receptors identified in the volcano plot. Data are presented as mean ± SD ($n = 3$ biological replicates).

Quality control of the MS data in Supplementary Fig. 10b

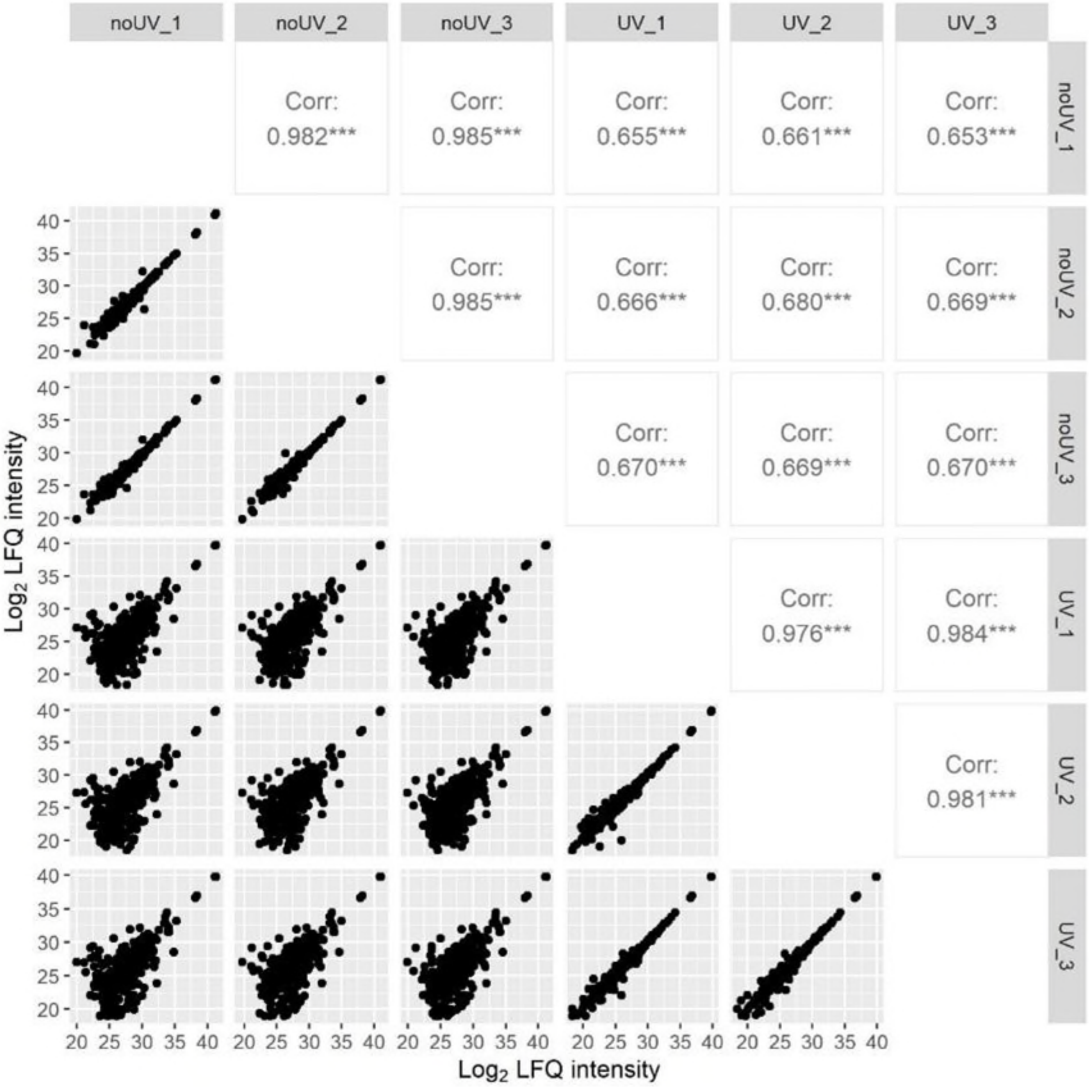

Supplementary Fig. 81 Pairwise Pearson correlation of the Log_2 transformed LFQ intensity before normalization and imputation.

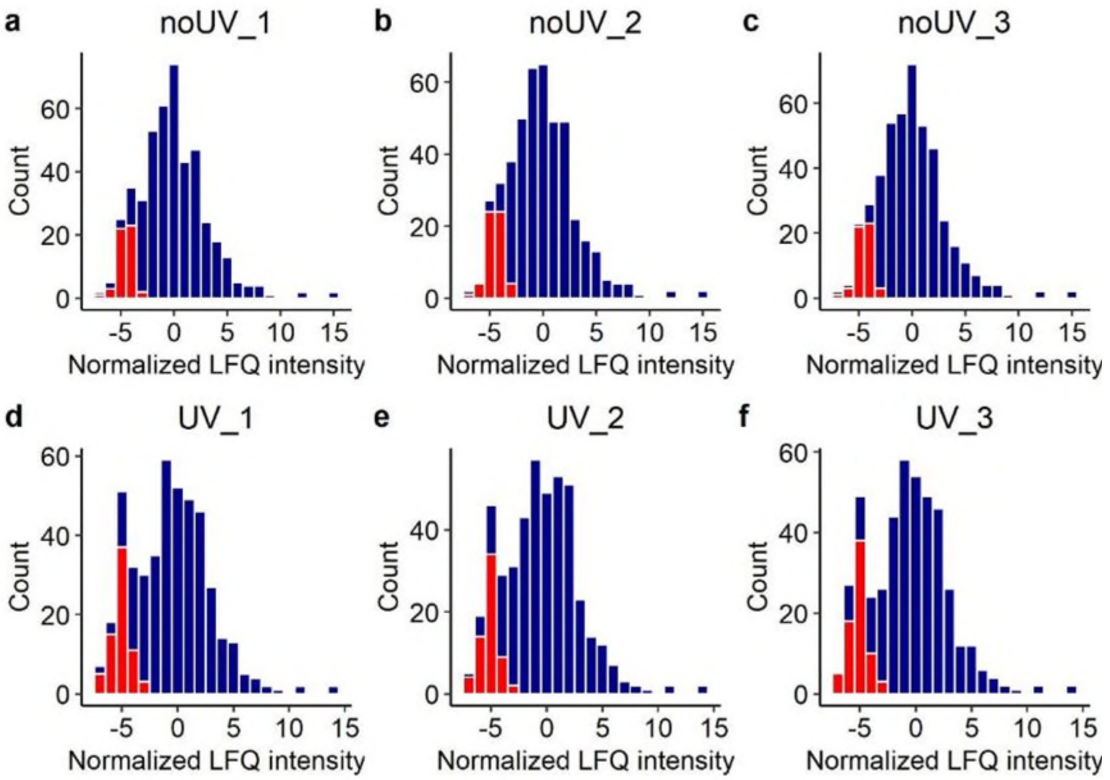

Supplementary Fig. 82 Histograms show the distribution of the normalized LFQ intensities in each sample (marked in blue), and the missing values that were imputed from the normal distribution are marked in red.

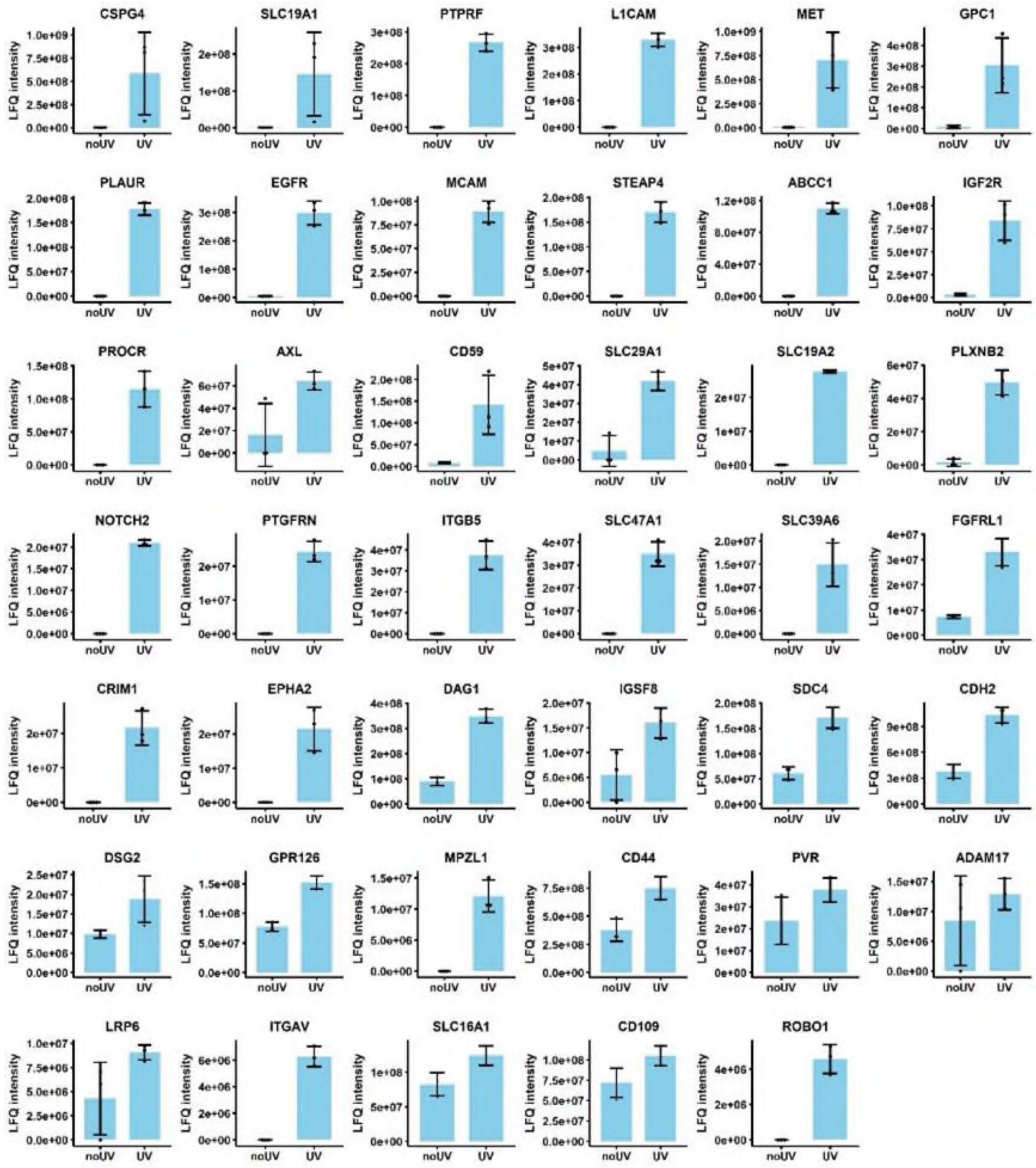

Supplementary Fig. 83 LFQ intensities of the significant receptors identified in the volcano plot. Data are presented as mean ± SD ($n = 3$ biological replicates).

Quality control of the MS data in Supplementary Fig. 12a

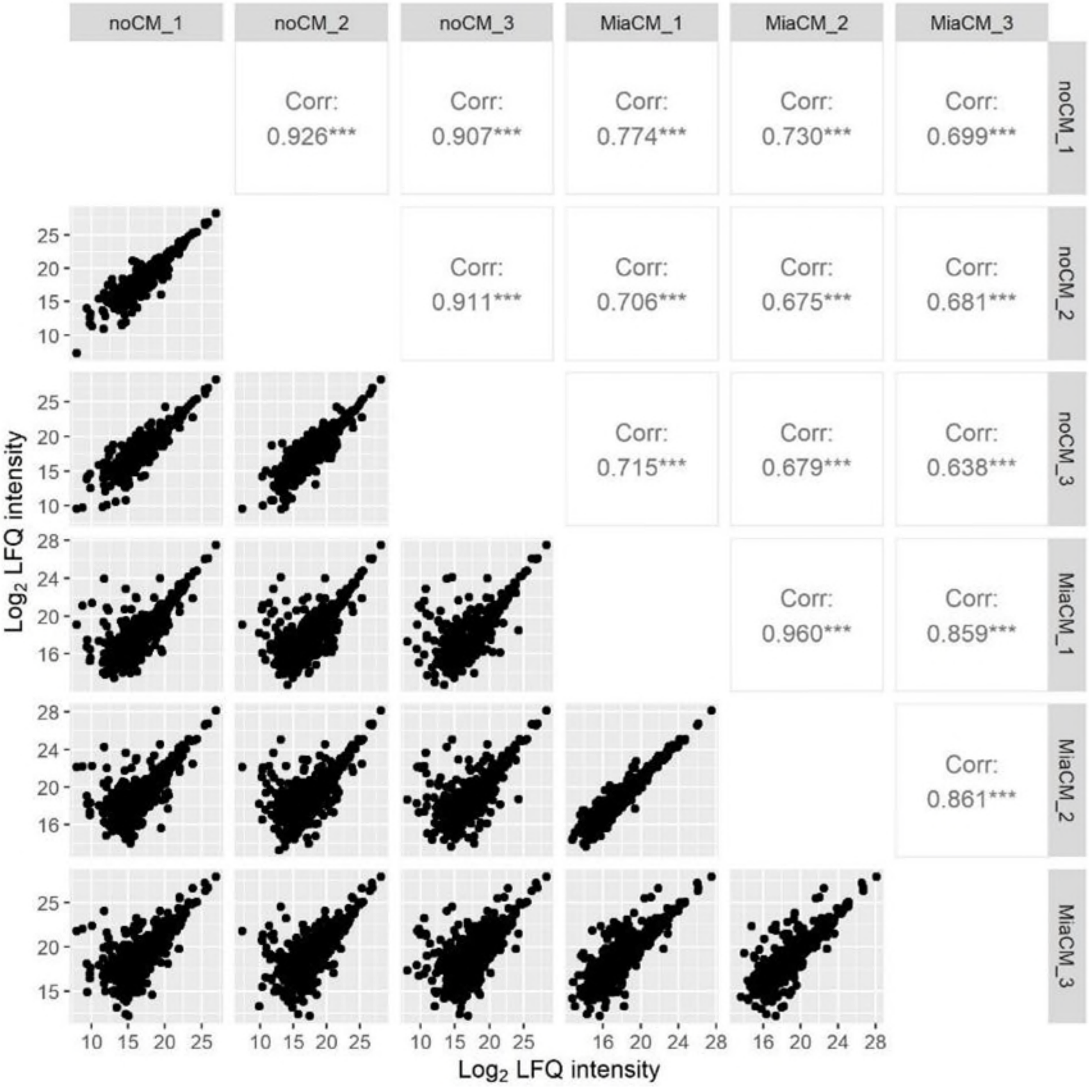

Supplementary Fig. 84 Pairwise Pearson correlation of the Log_2 transformed LFQ intensity before normalization and imputation.

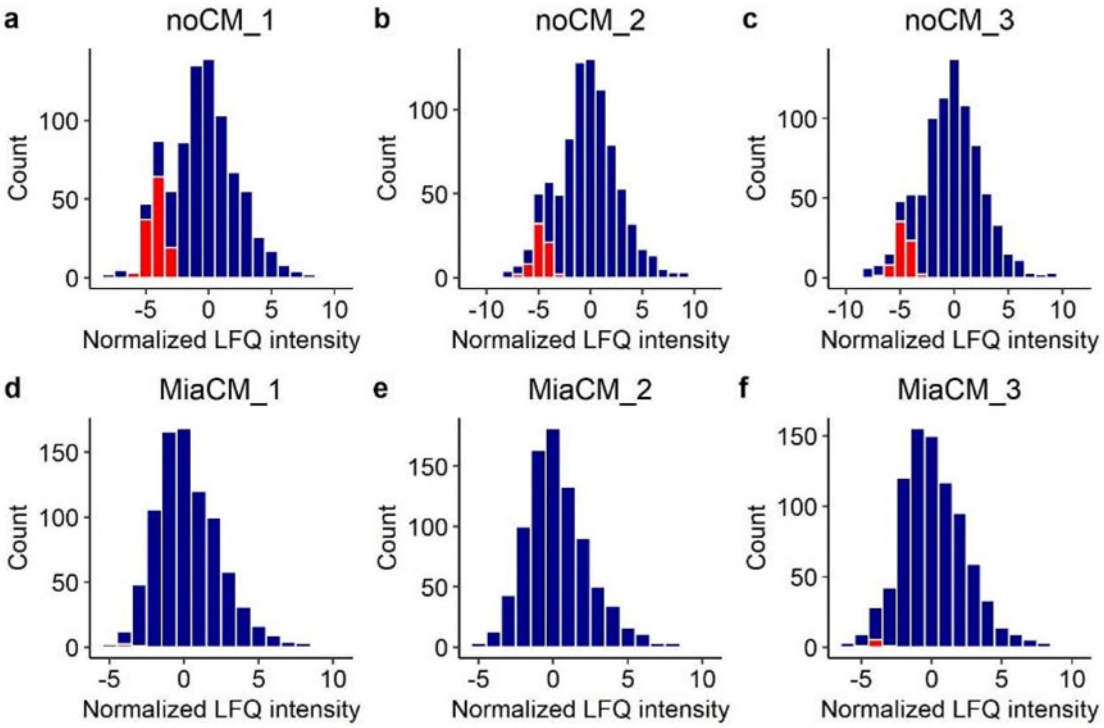

Supplementary Fig. 85 Histograms show the distribution of the normalized LFQ intensities in each sample (marked in blue), and the missing values that were imputed from the normal distribution are marked in red.

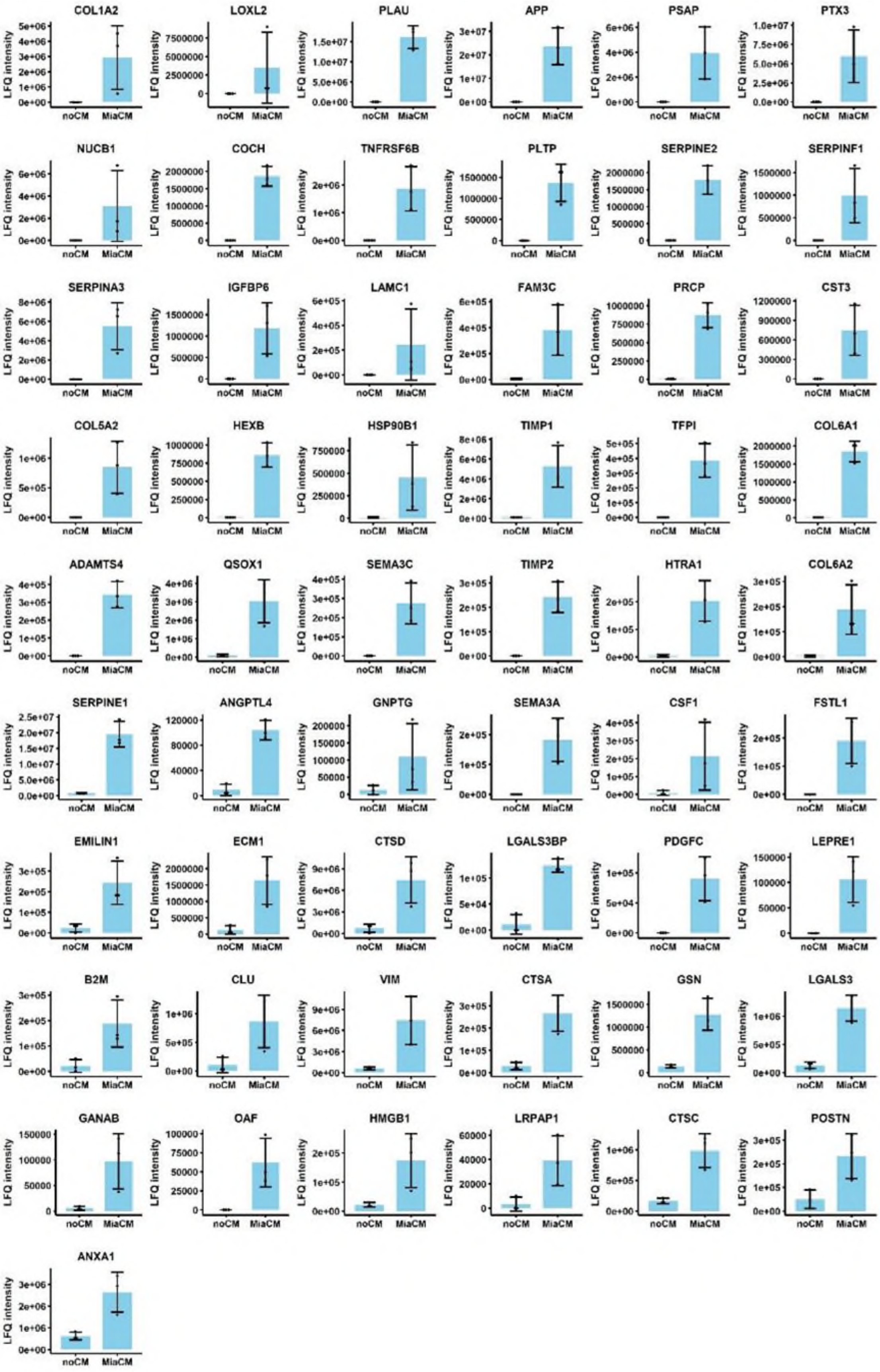

Supplementary Fig. 86 LFQ intensities of the significant ligands identified in the volcano plot. Data are presented as mean ± SD ($n = 3$ biological replicates).

Quality control of the MS data in Supplementary Fig. 12b

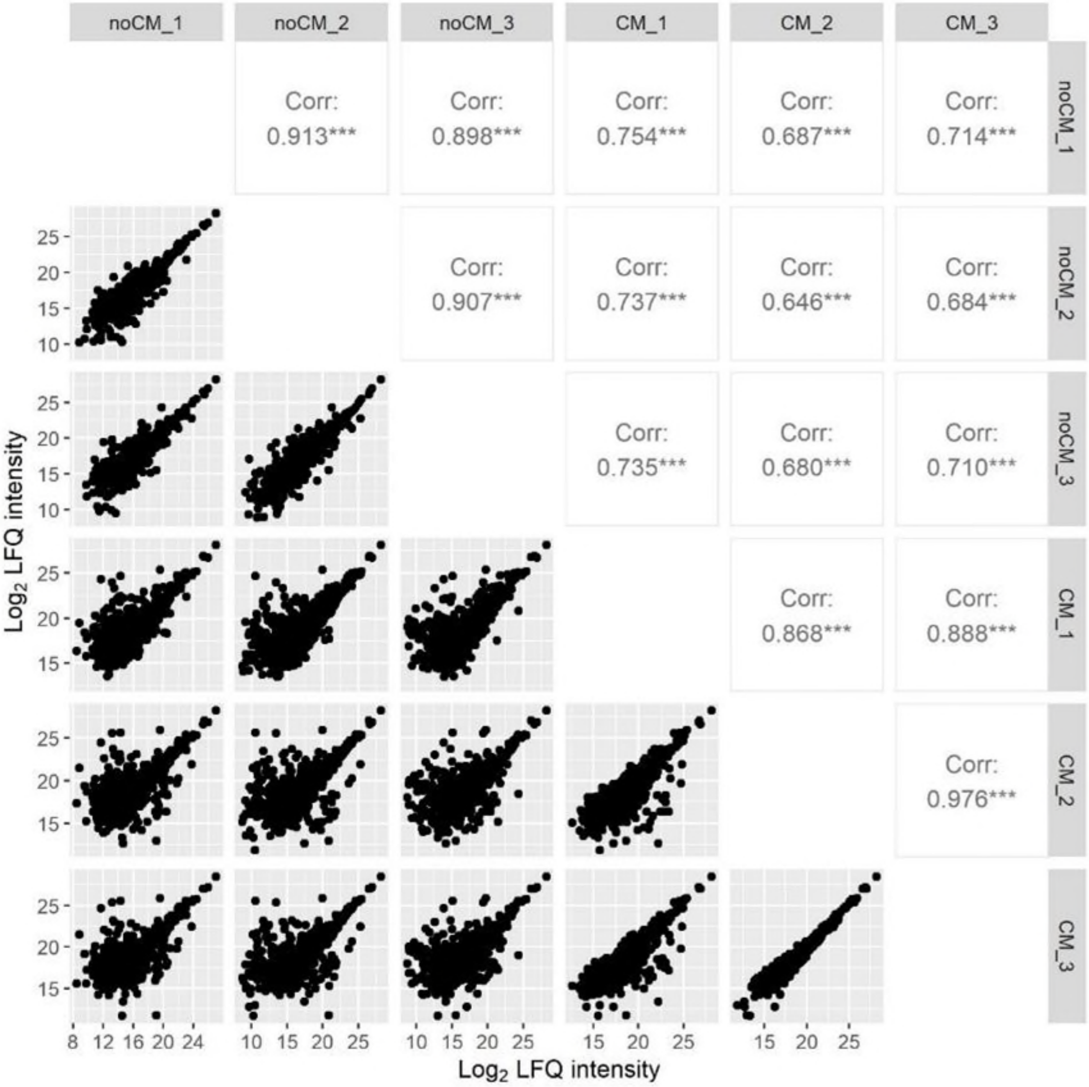

Supplementary Fig. 87 Pairwise Pearson correlation of the Log_2 transformed LFQ intensity before normalization and imputation.

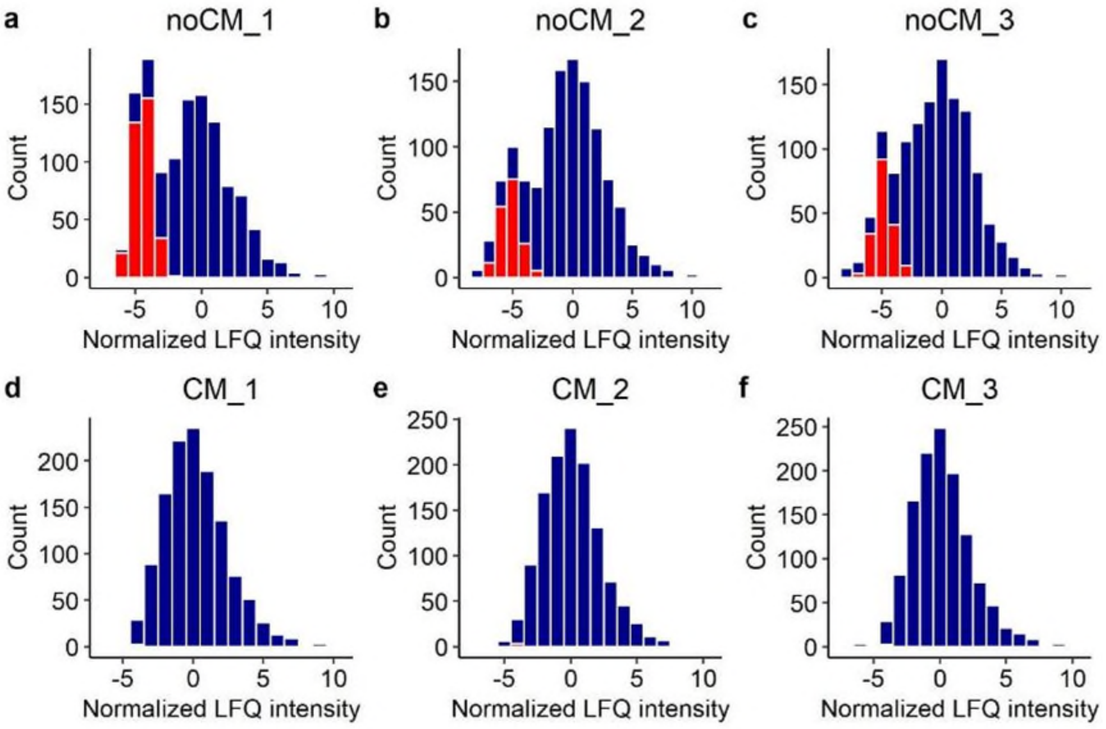

Supplementary Fig. 88 Histograms show the distribution of the normalized LFQ intensities in each sample (marked in blue), and the missing values that were imputed from the normal distribution are marked in red.

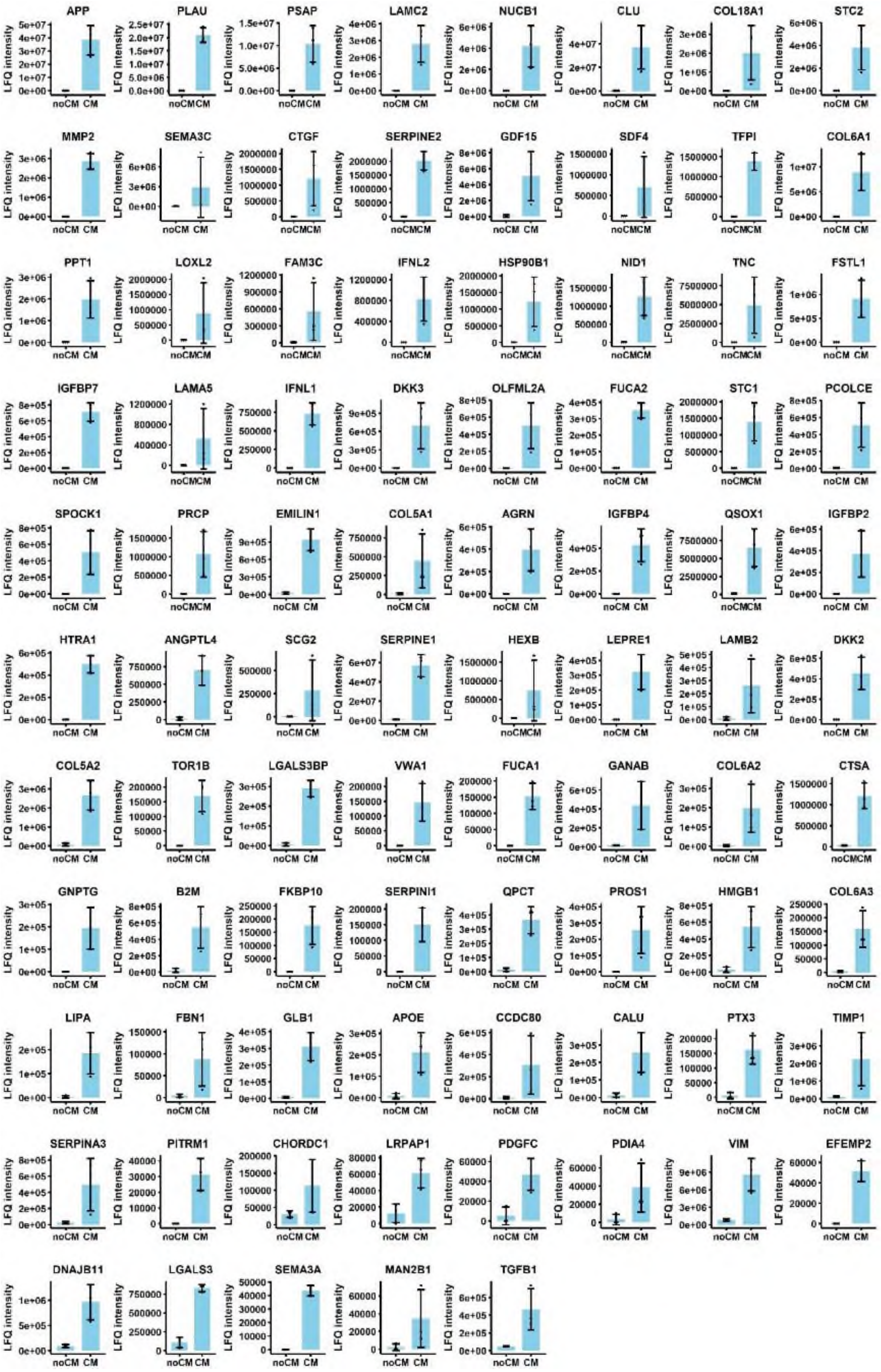

Supplementary Fig. 89 LFQ intensities of the significant ligands identified in the volcano plot. Data are

presented as mean ± SD ($n = 3$ biological replicates).

Quality control of the MS data in Supplementary Fig. 12c

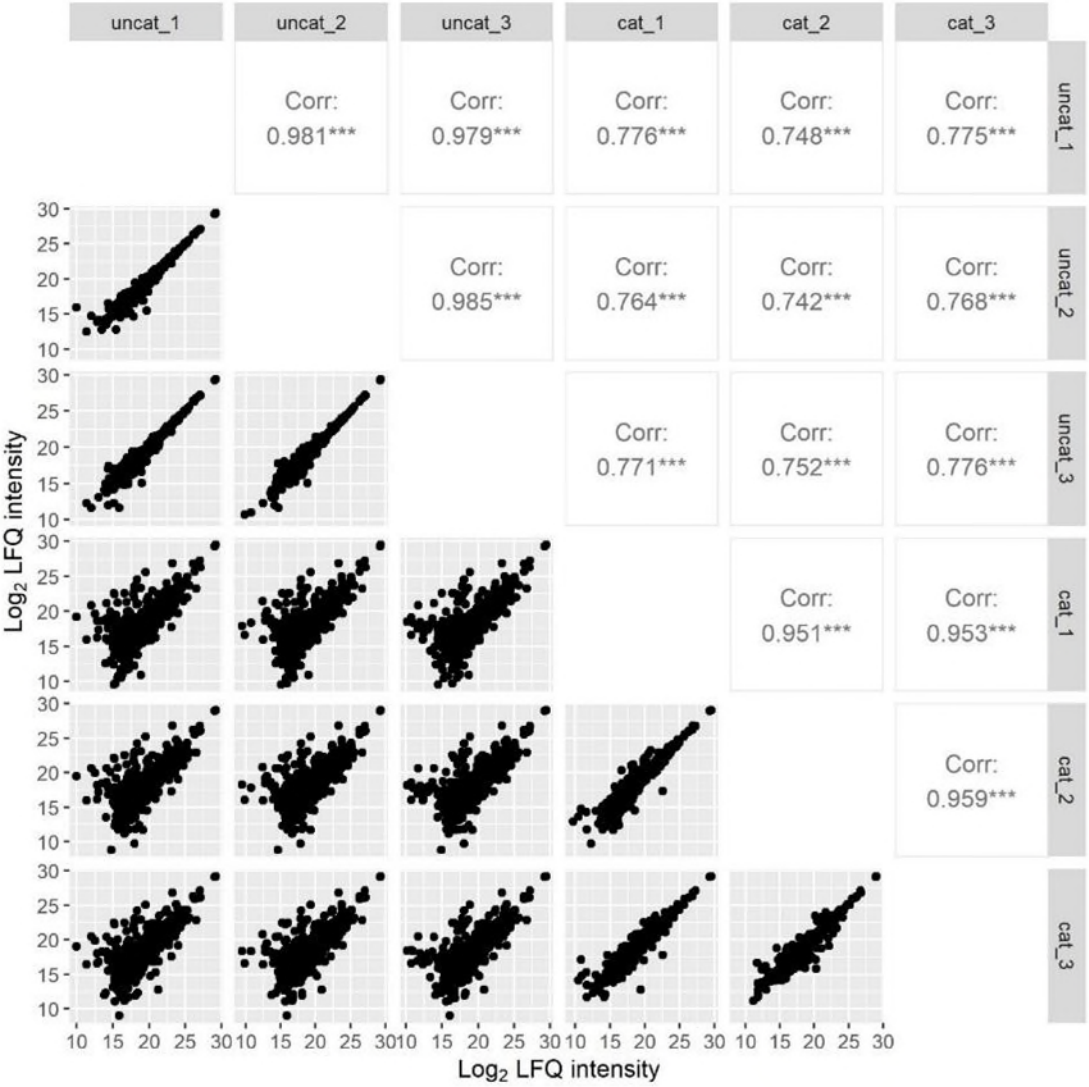

Supplementary Fig. 90 Pairwise Pearson correlation of the Log_2 transformed LFQ intensity before normalization and imputation.

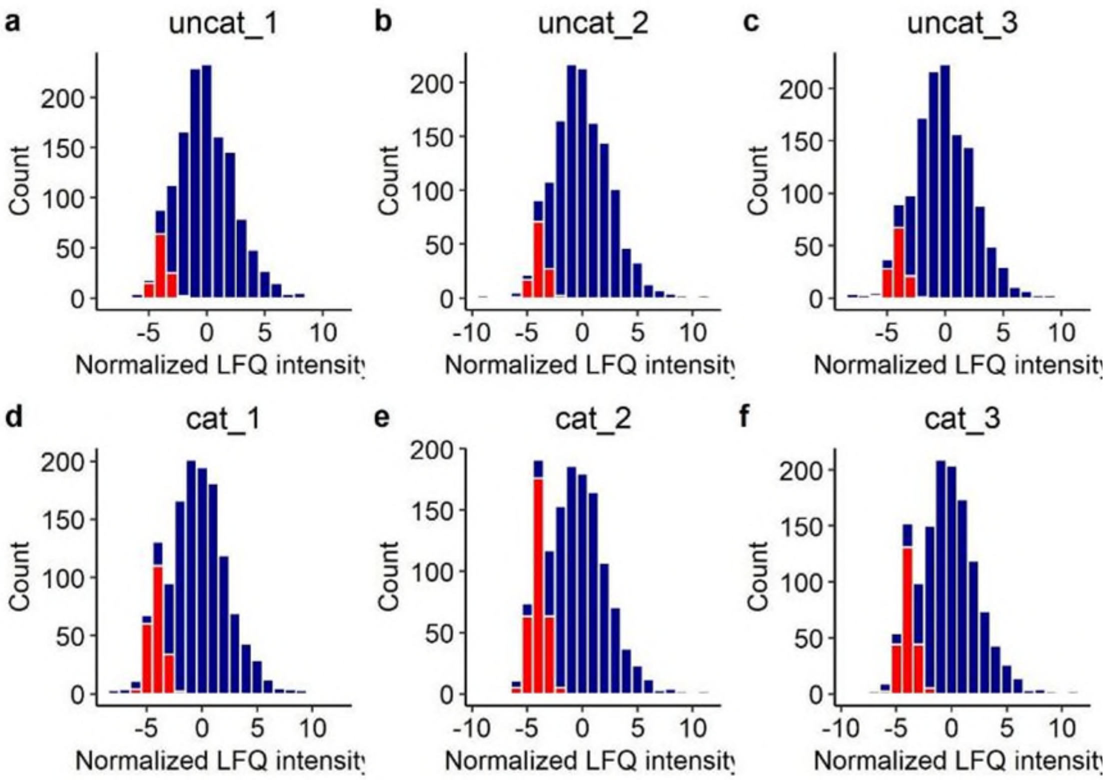

Supplementary Fig. 91 Histograms show the distribution of the normalized LFQ intensities in each sample (marked in blue), and the missing values that were imputed from the normal distribution are marked in red.

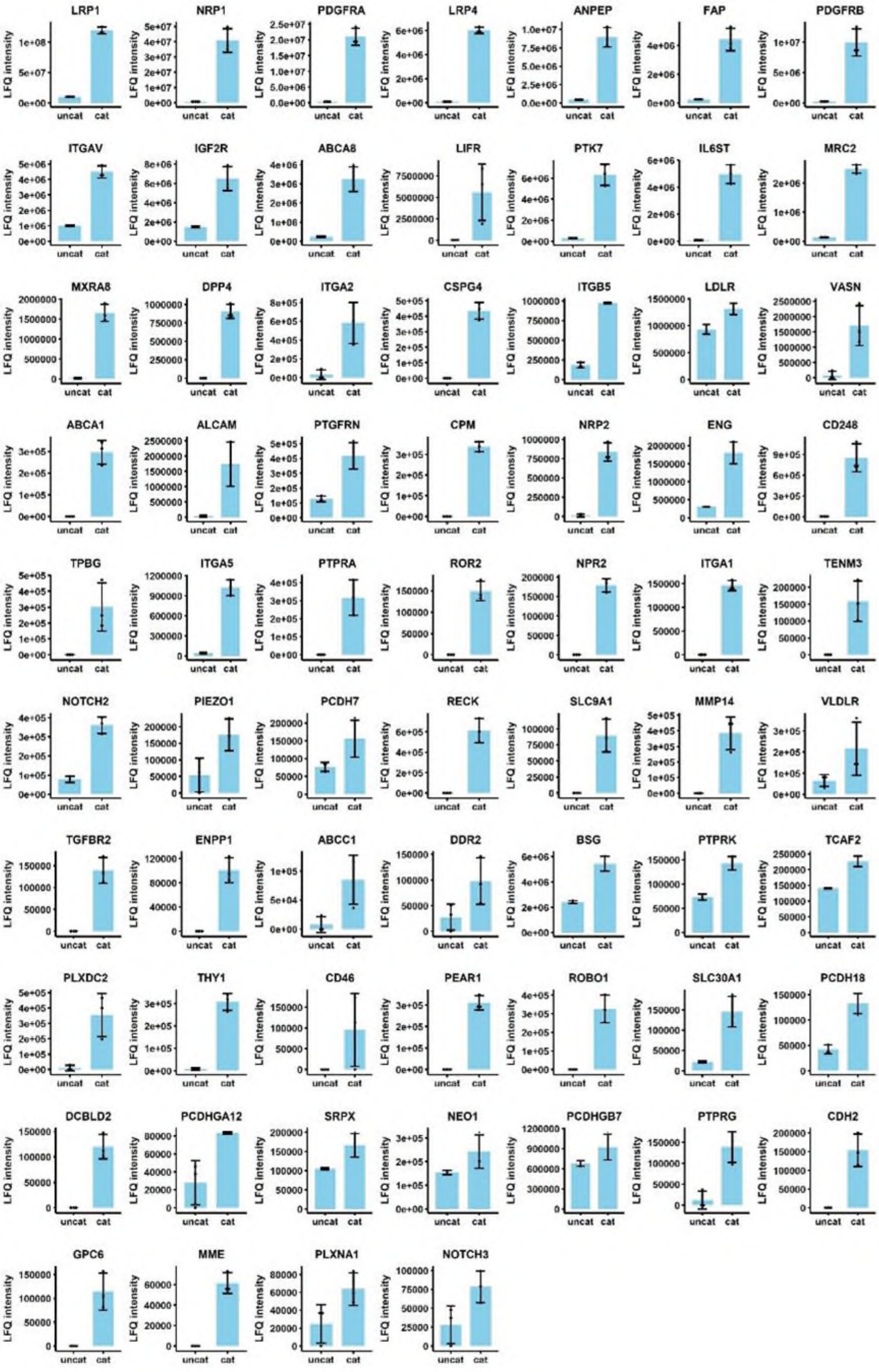

Supplementary Fig. 92 LFQ intensities of the significant receptors identified in the volcano plot. Data are presented as mean ± SD ($n = 3$ biological replicates).

Quality control of the MS data in Supplementary Fig. 12d

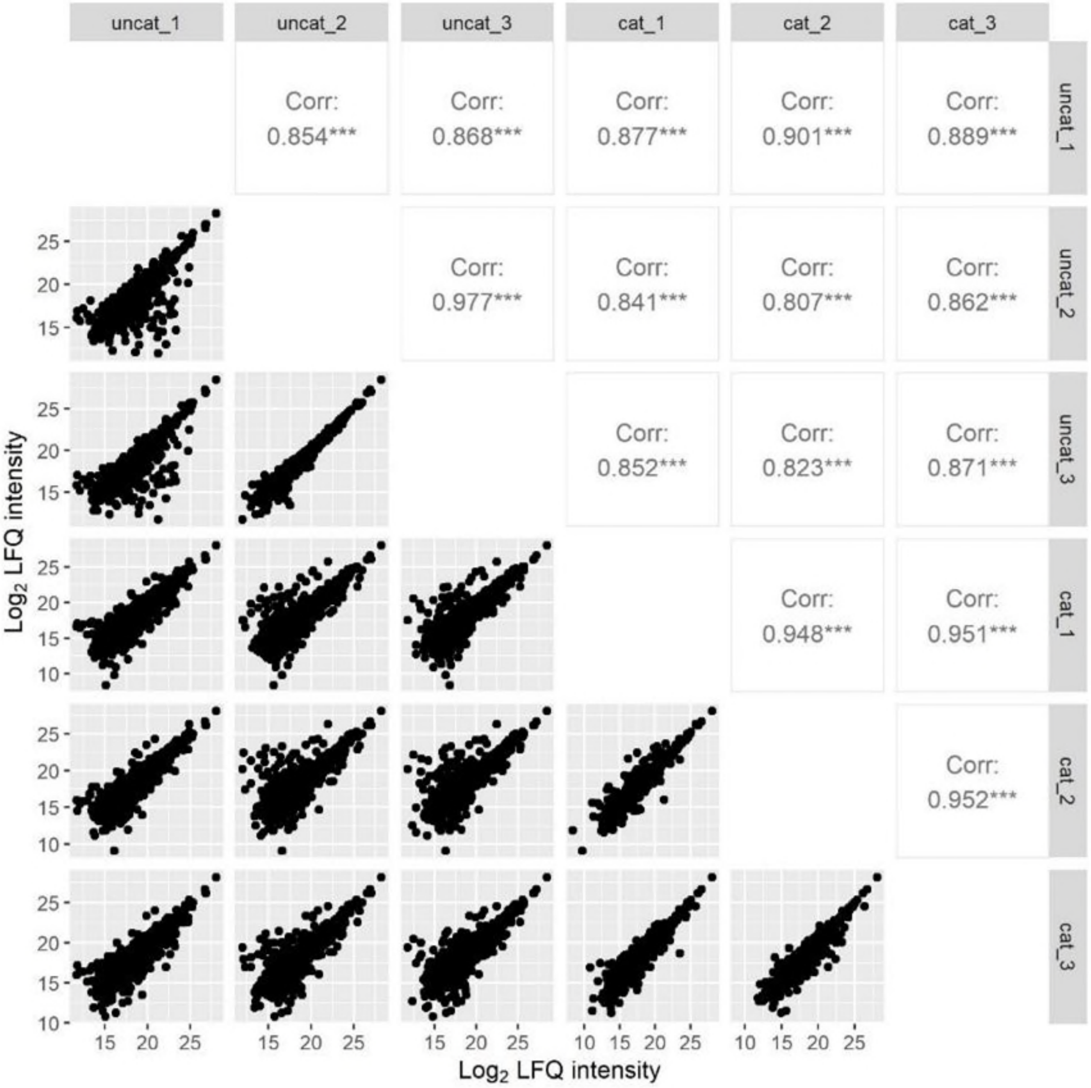

Supplementary Fig. 93 Pairwise Pearson correlation of the Log_2 transformed LFQ intensity before normalization and imputation.

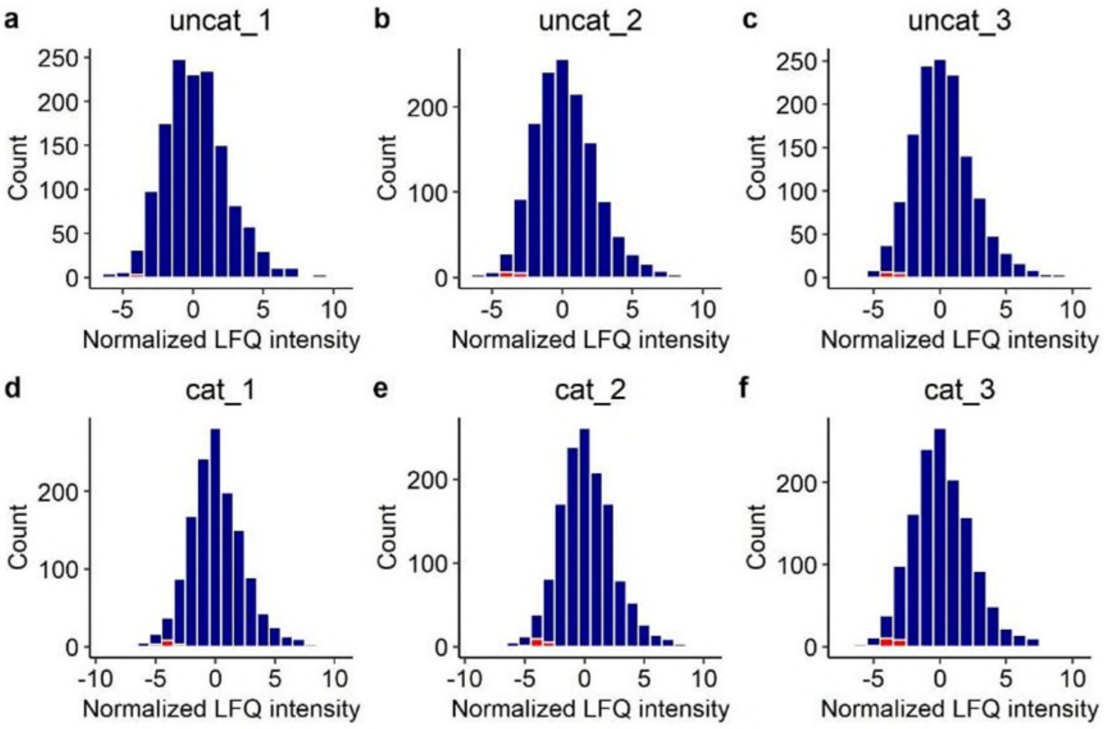

Supplementary Fig. 94 Histograms show the distribution of the normalized LFQ intensities in each sample (marked in blue), and the missing values that were imputed from the normal distribution are marked in red.

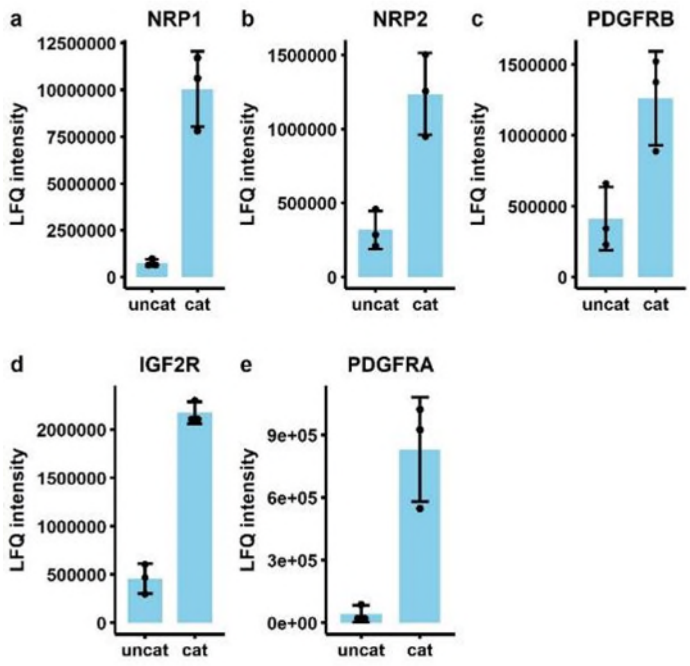

Supplementary Fig. 95 LFQ intensities of the significant receptors identified in the volcano plot. Data are

presented as mean ± SD ($n = 3$ biological replicates).

Quality control of the MS data in Supplementary Fig. 13a

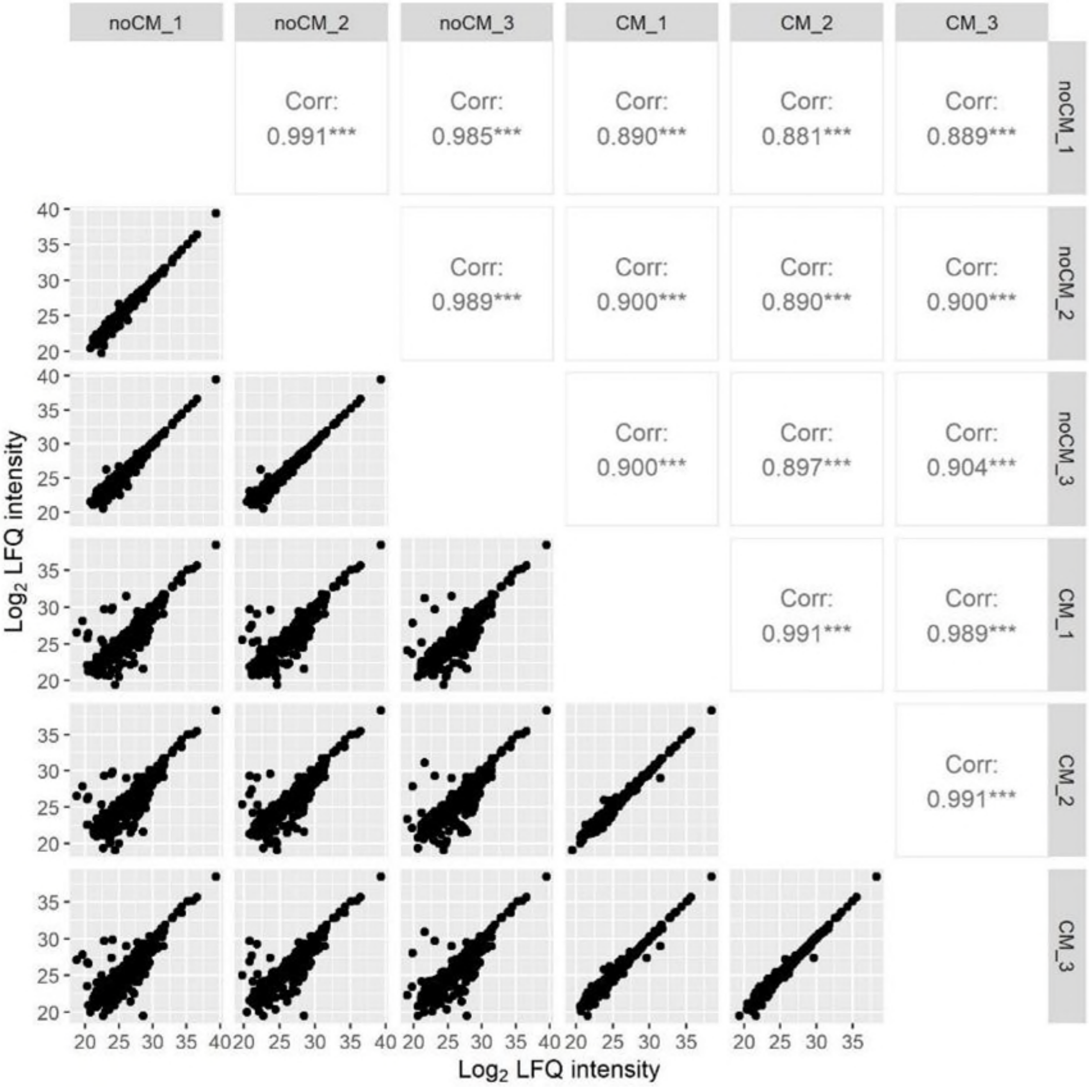

Supplementary Fig. 96 Pairwise Pearson correlation of the Log_2 transformed LFQ intensity before normalization and imputation.

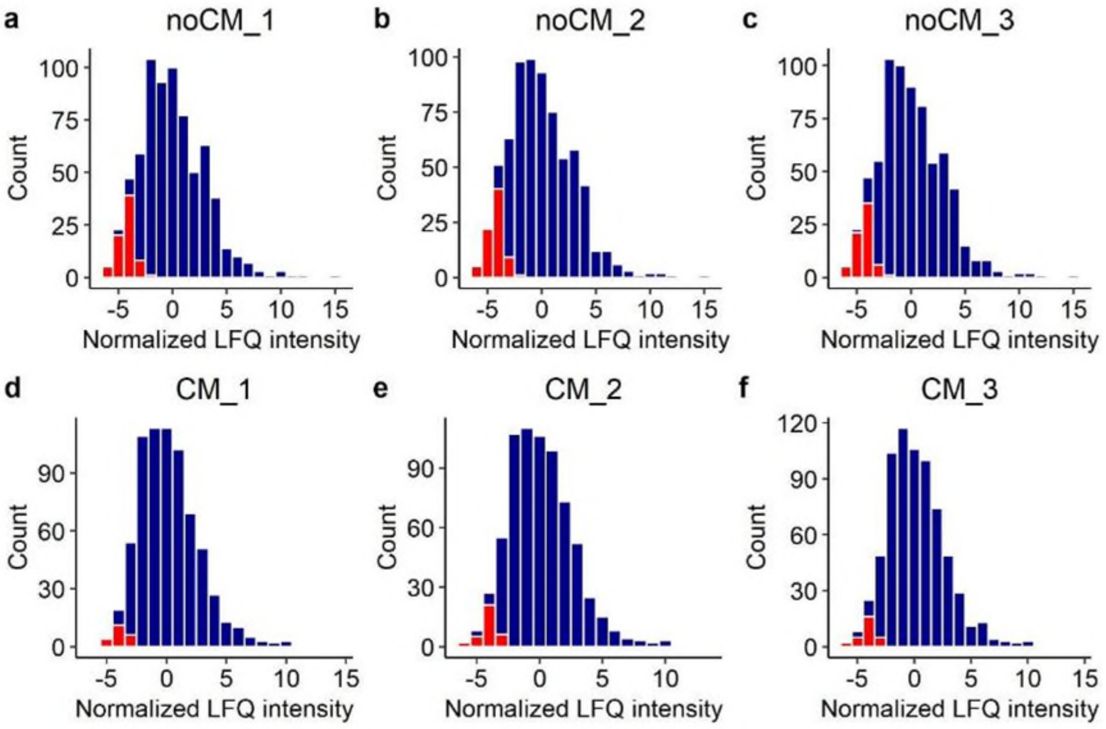

Supplementary Fig. 97 Histograms show the distribution of the normalized LFQ intensities in each sample (marked in blue), and the missing values that were imputed from the normal distribution are marked in red.

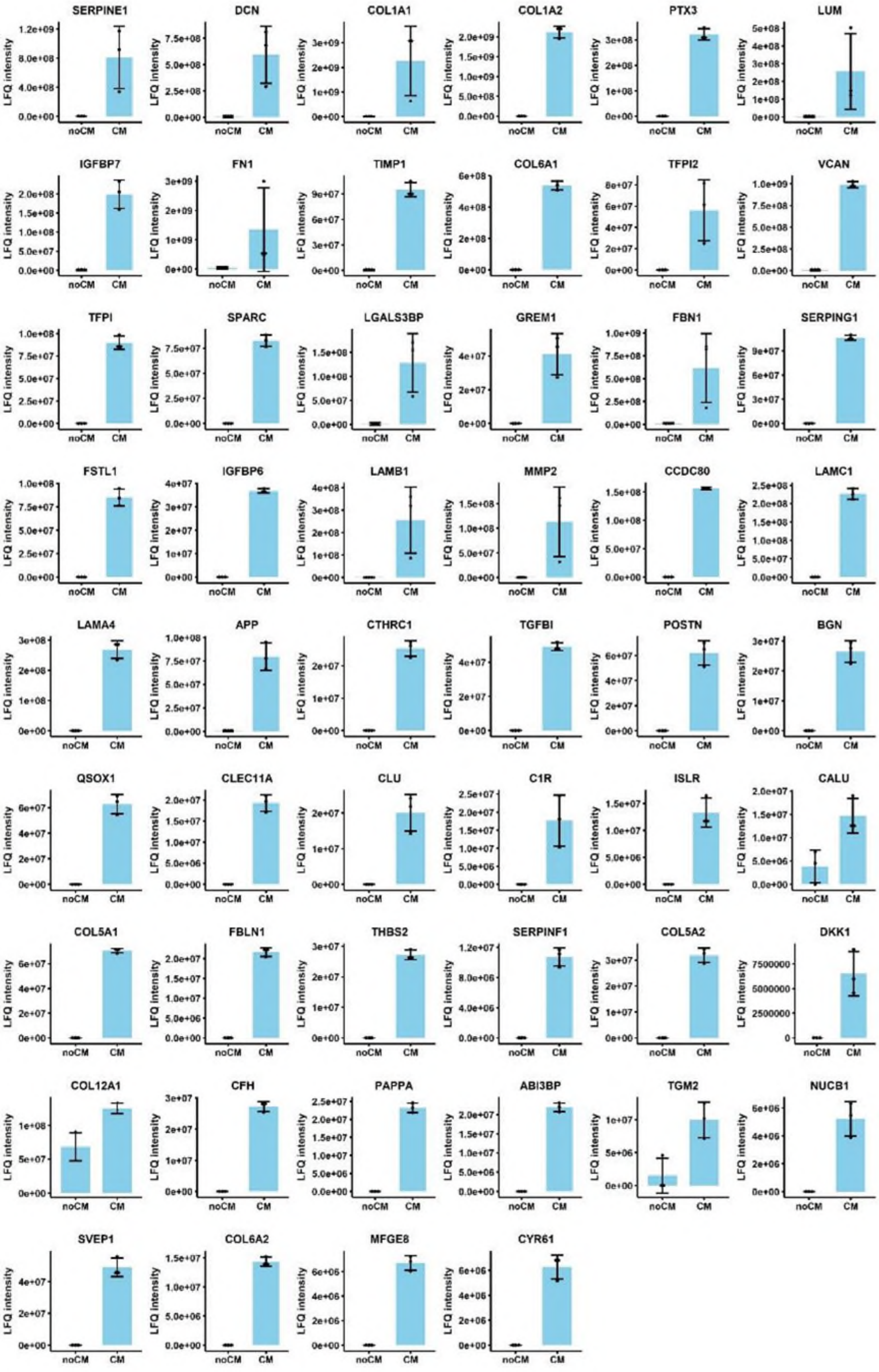

Supplementary Fig. 98 LFQ intensities of the significant ligands identified in the volcano plot. Data are presented as mean ± SD ($n = 3$ biological replicates).

Quality control of the MS data in Supplementary Fig. 13b

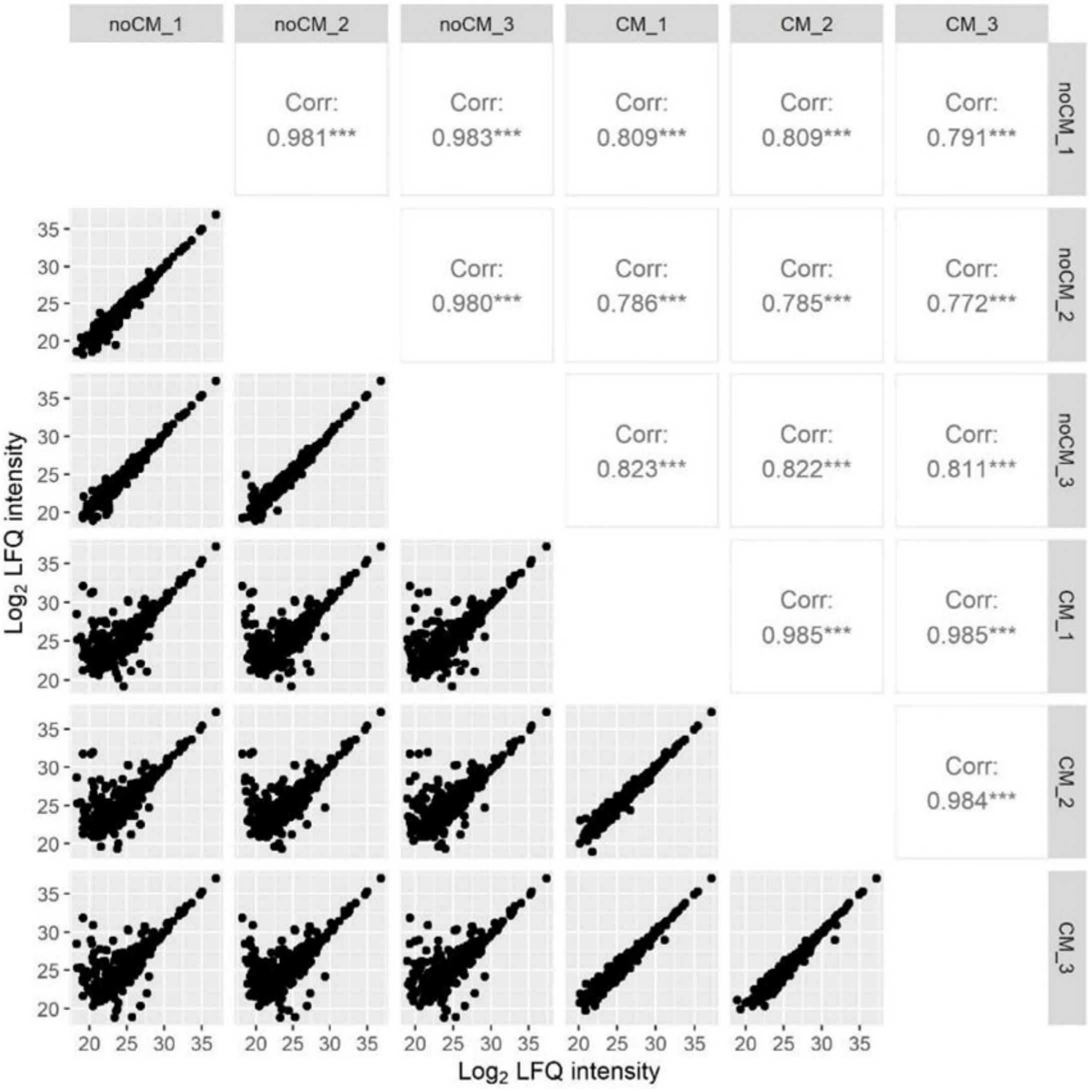

Supplementary Fig. 99 Pairwise Pearson correlation of the Log_2 transformed LFQ intensity before normalization and imputation.

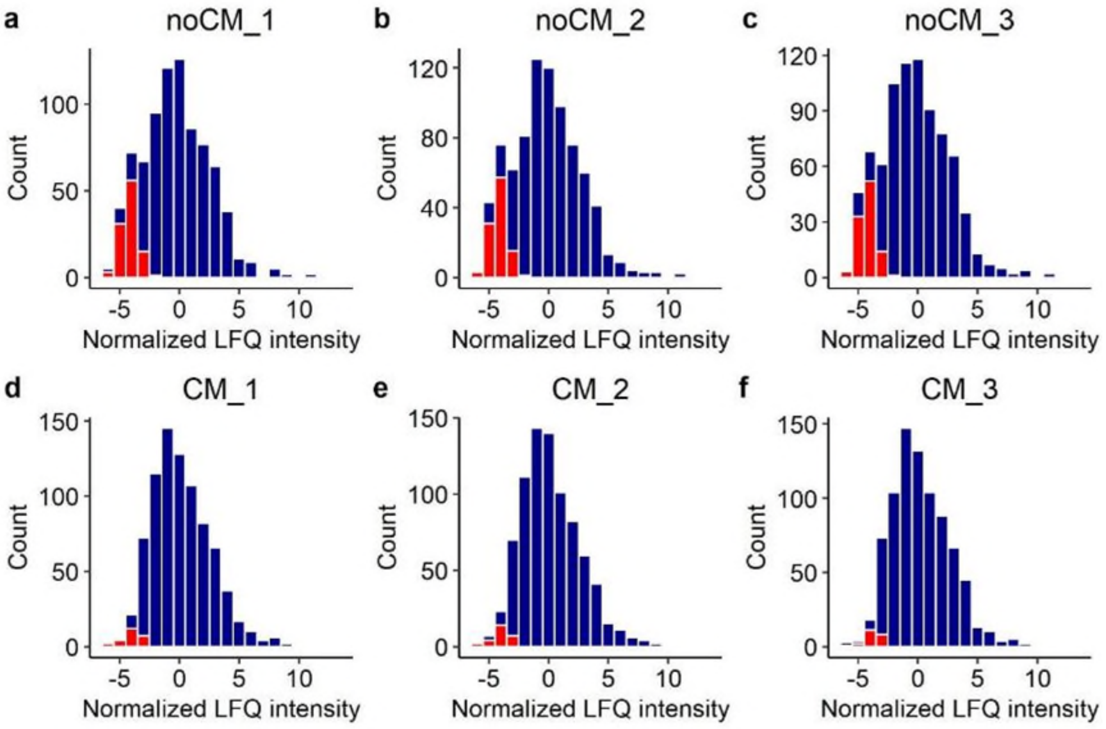

Supplementary Fig. 100 Histograms show the distribution of the normalized LFQ intensities in each sample (marked in blue), and the missing values that were imputed from the normal distribution are marked in red.

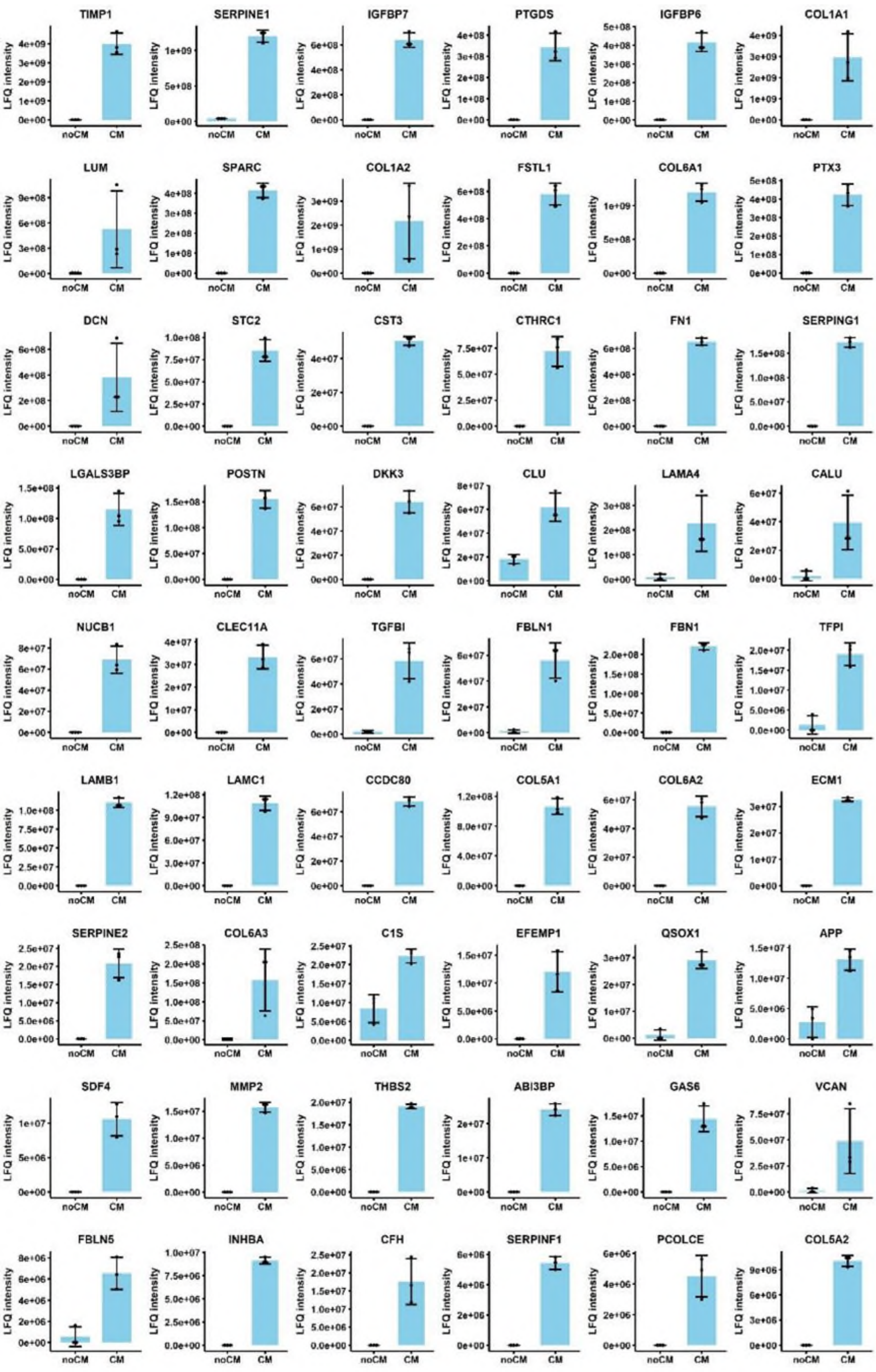

Supplementary Fig. 101 LFQ intensities of the significant ligands identified in the volcano plot. Data are presented as mean ± SD ($n = 3$ biological replicates).

Quality control of the MS data in Supplementary Fig. 13c

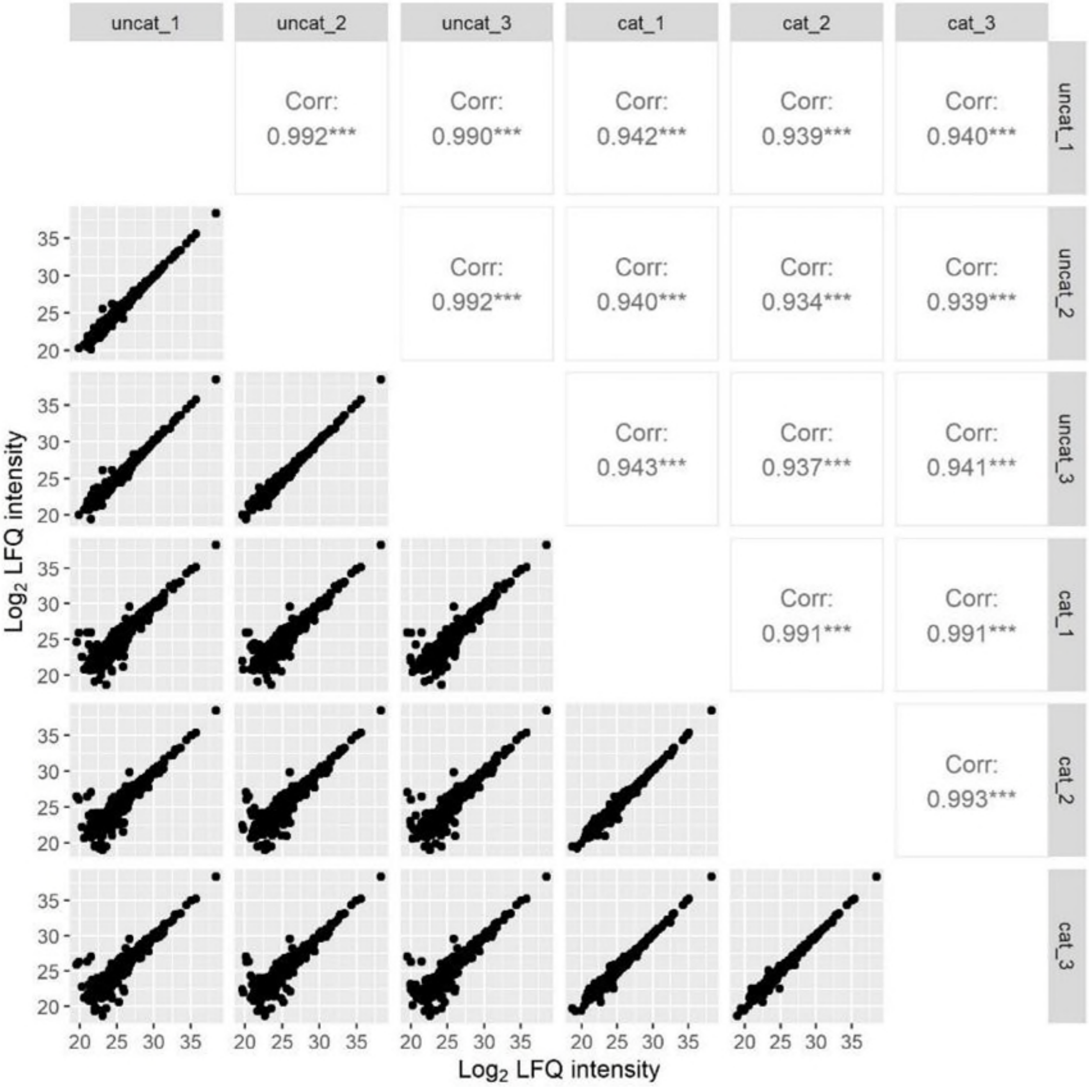

Supplementary Fig. 102 Pairwise Pearson correlation of the Log_2 transformed LFQ intensity before normalization and imputation.

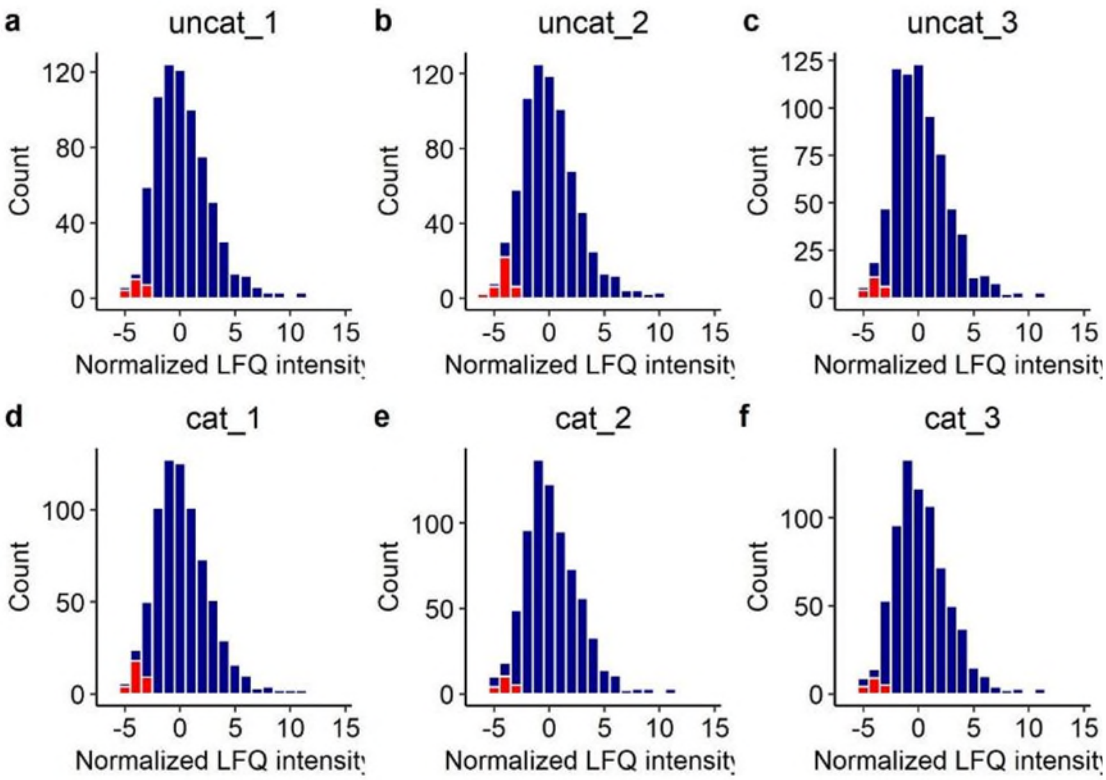

Supplementary Fig. 103 Histograms show the distribution of the normalized LFQ intensities in each sample (marked in blue), and the missing values that were imputed from the normal distribution are marked in red.

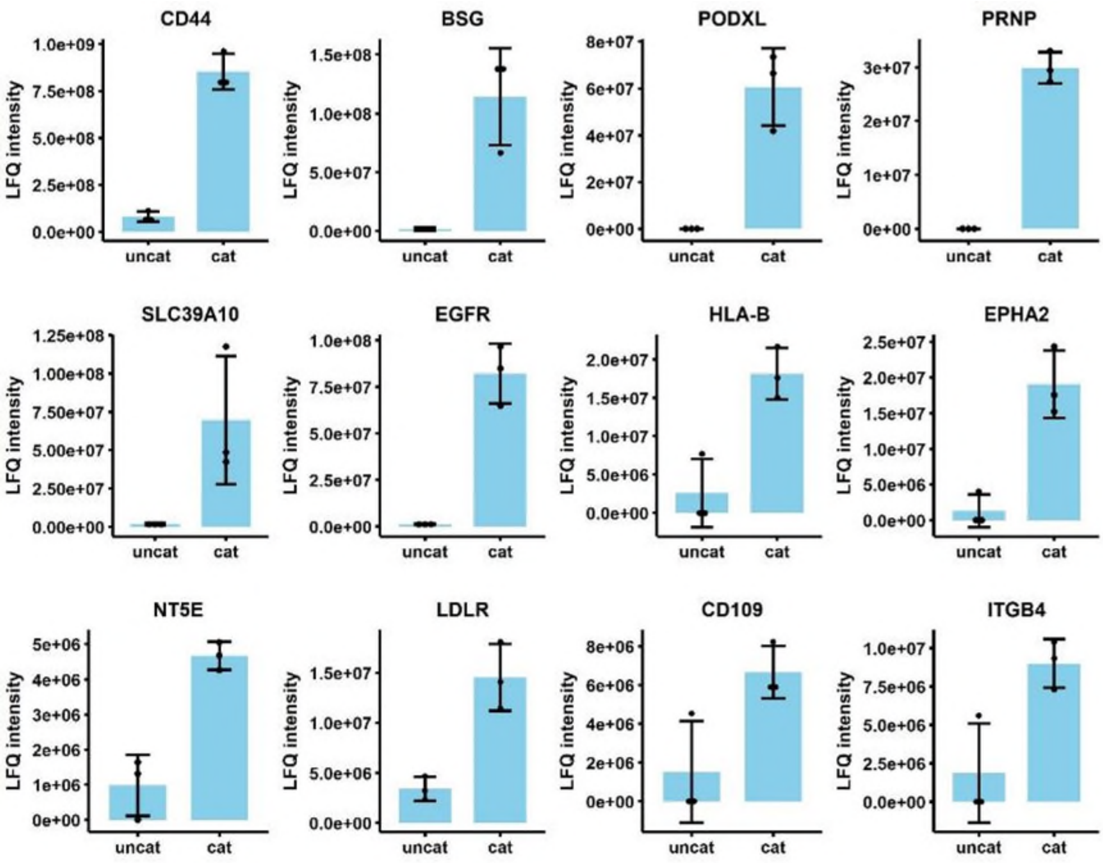

Supplementary Fig. 104 LFQ intensities of the significant receptors identified in the volcano plot. Data are presented as mean ± SD ($n = 3$ biological replicates).

Quality control of the MS data in Supplementary Fig. 13d

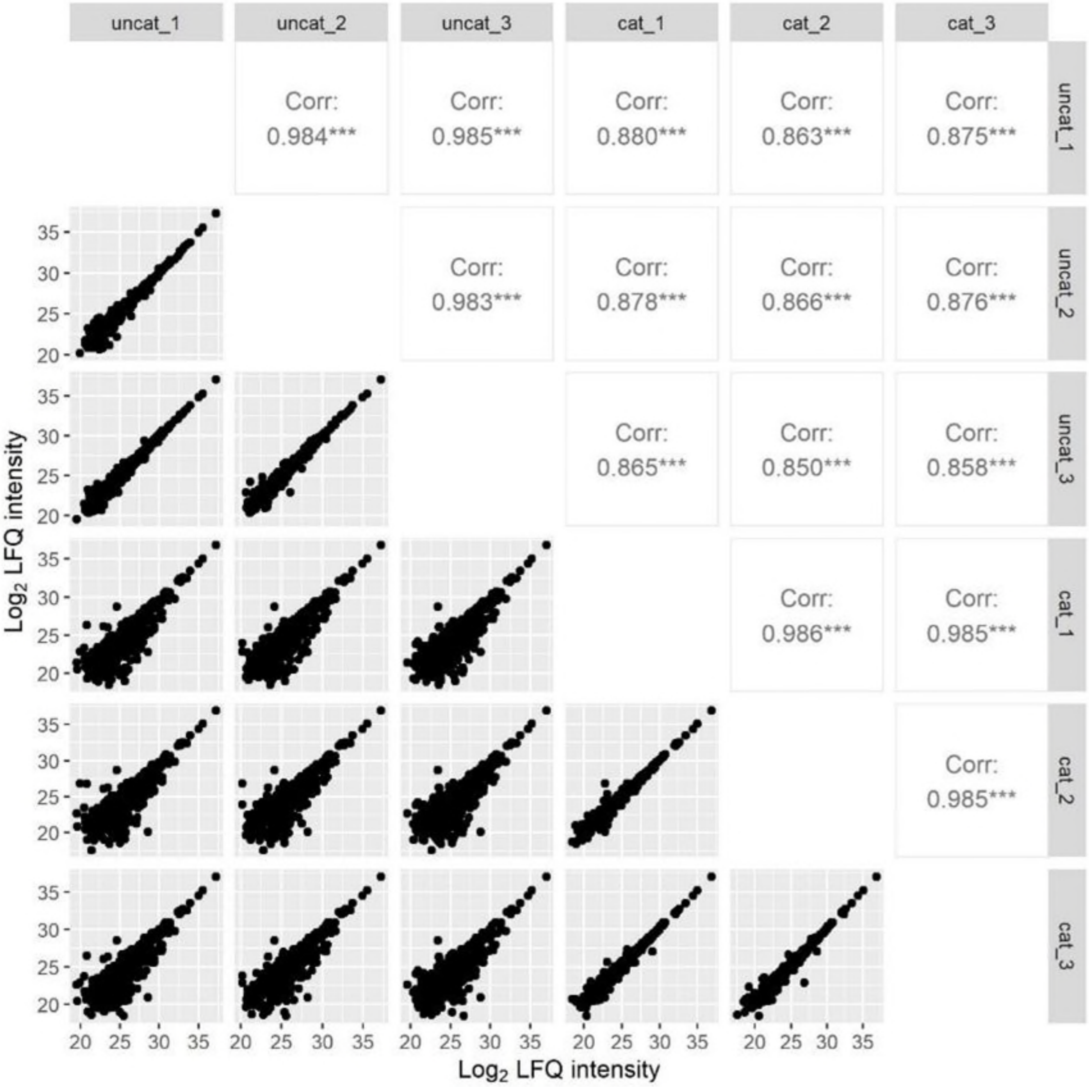

Supplementary Fig. 105 Pairwise Pearson correlation of the Log_2 transformed LFQ intensity before normalization and imputation.

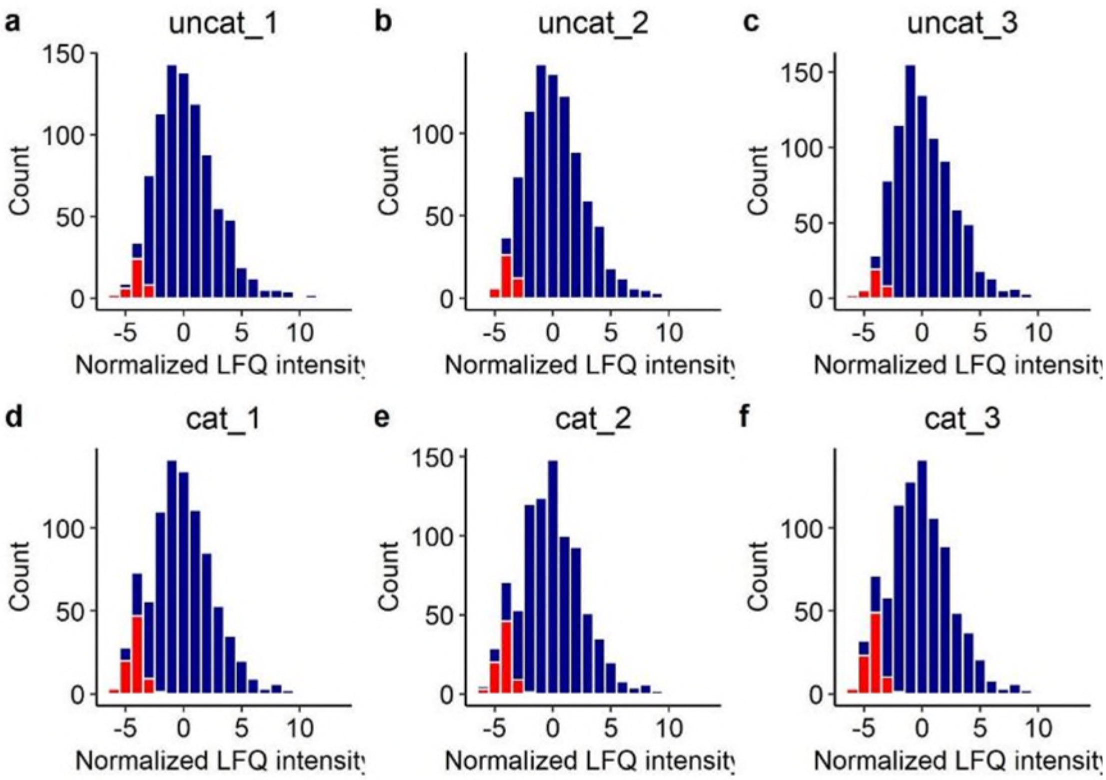

Supplementary Fig. 106 Histograms show the distribution of the normalized LFQ intensities in each sample (marked in blue), and the missing values that were imputed from the normal distribution are marked in red.

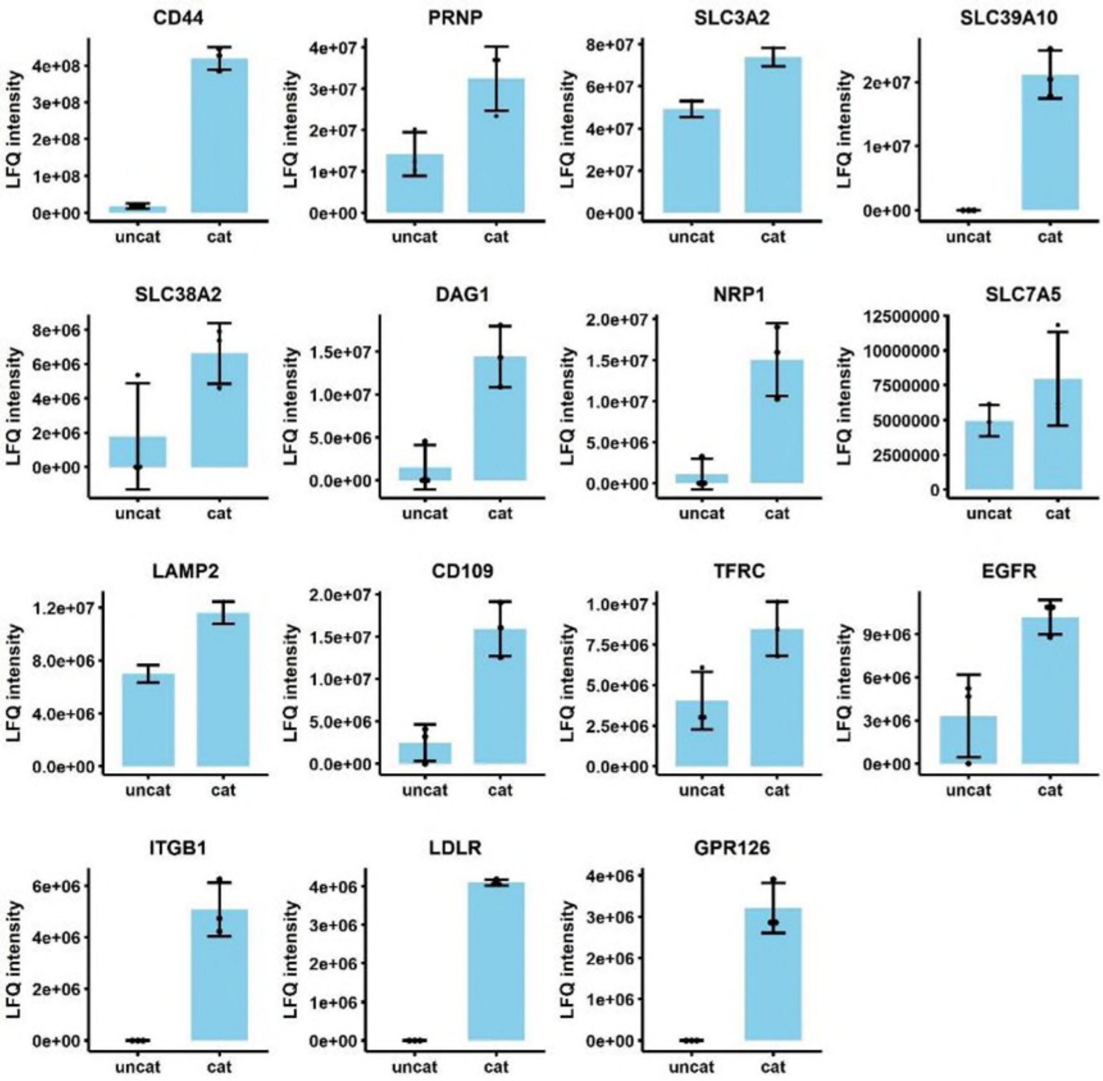

Supplementary Fig. 107 LFQ intensities of the significant receptors identified in the volcano plot. Data are presented as mean ± SD (*n* = 3 biological replicates).

Quality control of the MS data in Supplementary Fig. 14a

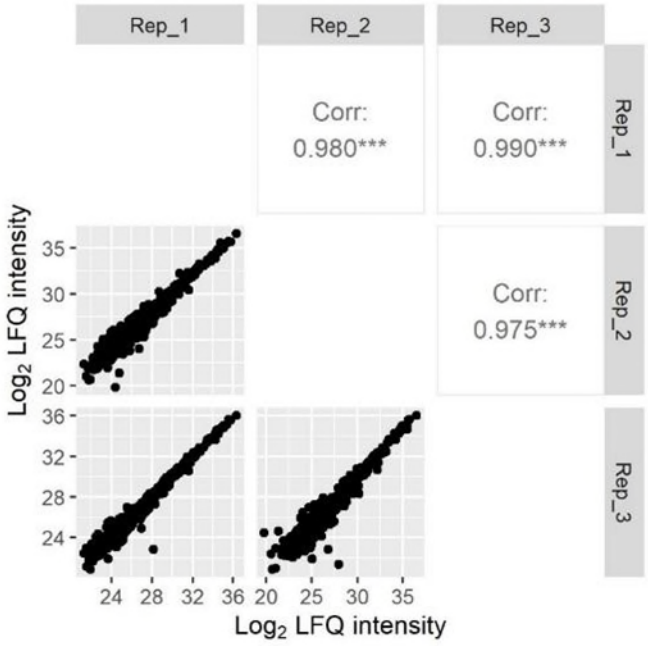

Supplementary Fig. 108 Pairwise Pearson correlation of the Log_2 transformed LFQ intensity before normalization and imputation.

Quality control of the MS data in Supplementary Fig. 14b

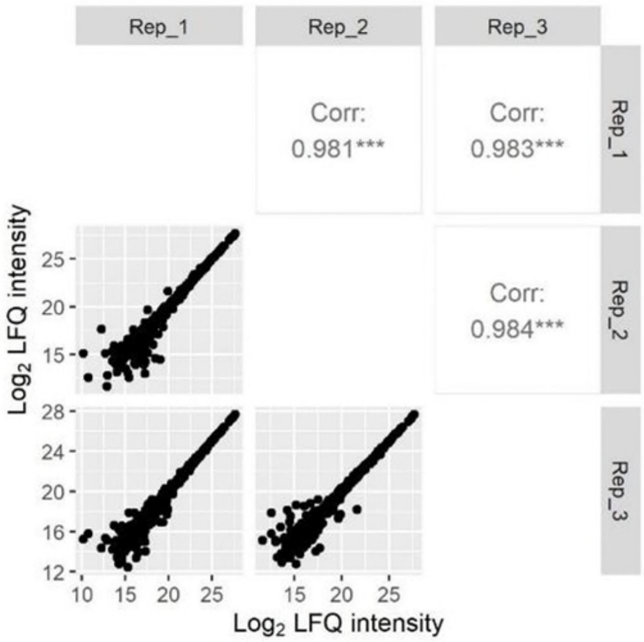

Supplementary Fig. 109 Pairwise Pearson correlation of the Log_2 transformed LFQ intensity before

normalization and imputation.

Quality control of the MS data in Supplementary Fig. 14c

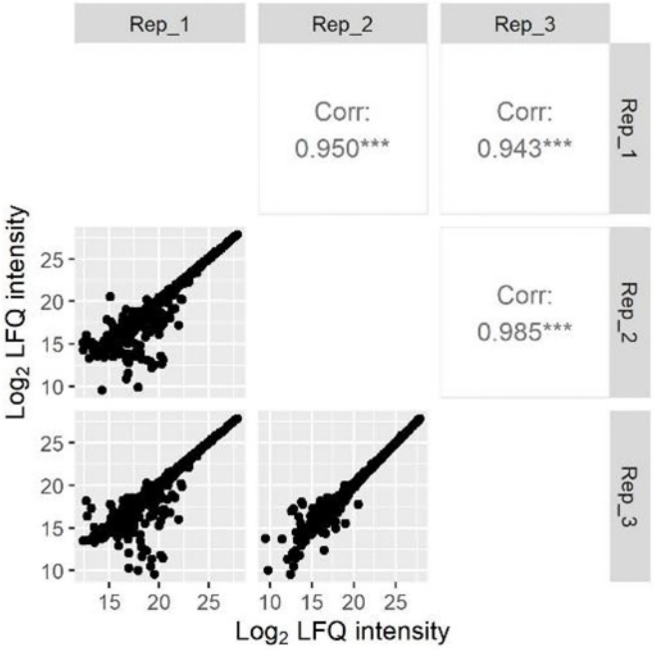

Supplementary Fig. 110 Pairwise Pearson correlation of the Log_2 transformed LFQ intensity before normalization and imputation.

Quality control of the MS data in Supplementary Fig. 14d

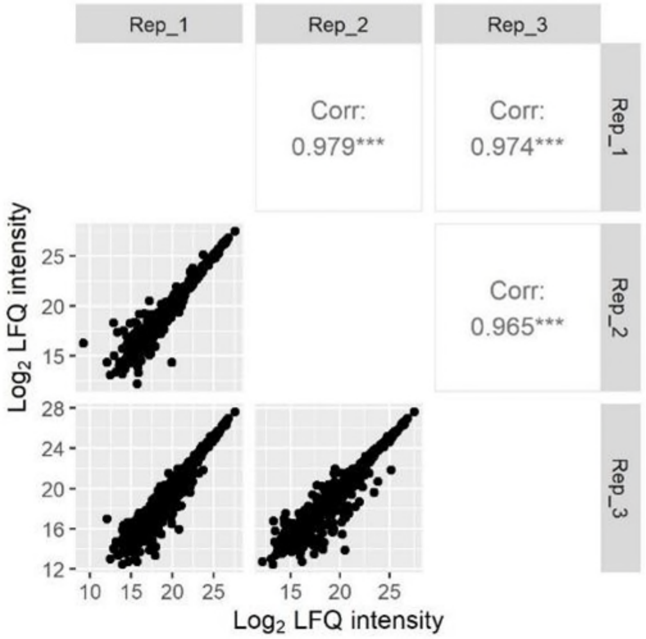

Supplementary Fig. 111 Pairwise Pearson correlation of the Log_2 transformed LFQ intensity before normalization and imputation.

Quality control of the MS data in Supplementary Fig. 14e

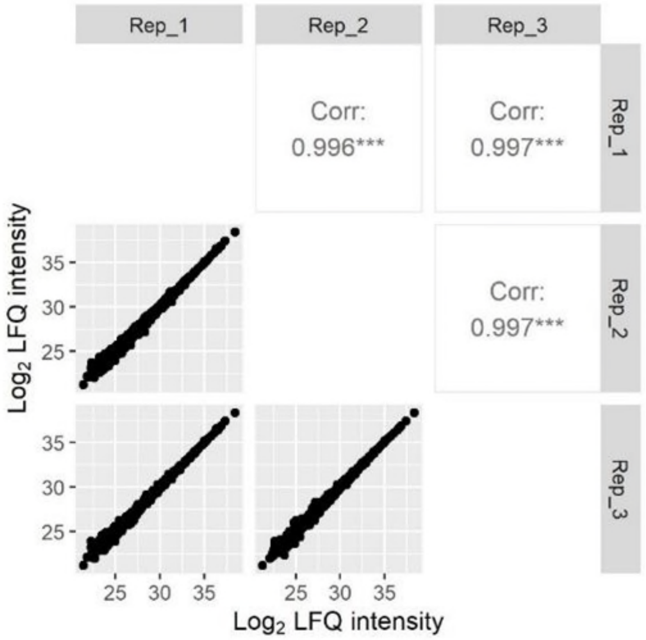

Supplementary Fig. 112 Pairwise Pearson correlation of the Log_2 transformed LFQ intensity before normalization and imputation.

Quality control of the MS data in Supplementary Fig. 14f

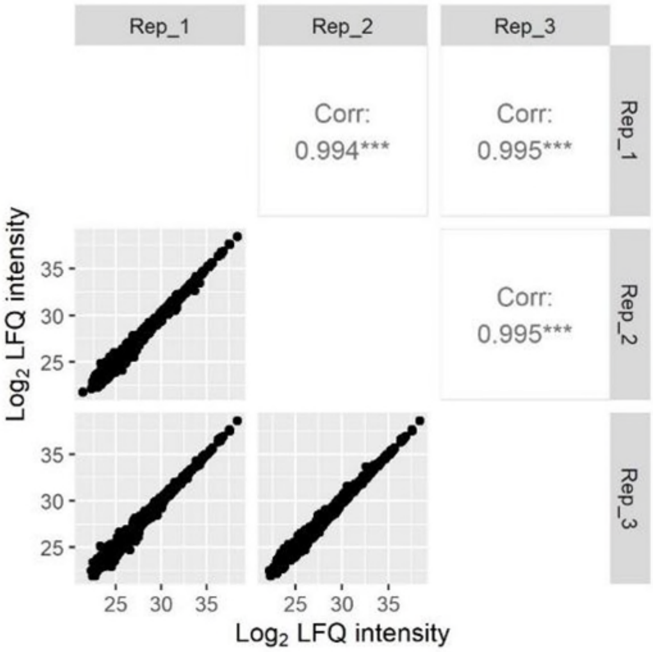

Supplementary Fig. 113 Pairwise Pearson correlation of the Log_2 transformed LFQ intensity before normalization and imputation.

Supplementary Tables

Supplementary Table 1. Primers used for real-time PCR

Gene Symbol	Direction	Primer sequences
PLAU	Forward	GGGAATGGTCACTTTTACCGAG
	Reverse	GGGCATGGTACGTTTGCTG
NRP1	Forward	ACGTGGAAGTCTTCGATGGAG
	Reverse	CACCATGTGTTTCGTAGTCAGA

Supplementary Table 2. siRNAs used for knock down

Gene Symbol	Target sequences (5'-3')
PLAU #1	GCUCAAGGCUUAACUCCAA
PLAU #2	GAAAAUGACUGUUGUGAAG
NRP1 #1	AAUCAGAGUUUCCAACAUA
NRP1 #2	GGAGAACUAUAACUUUGAA

References

1. Chu, B. *et al.* Photoaffinity-engineered protein scaffold for systematically exploring native phosphotyrosine signaling complexes in tumor samples. *Proc. Natl. Acad. Sci. U.S.A.* **115**, E8863-E8872 (2018).
2. Meier, F. *et al.* Online Parallel Accumulation-Serial Fragmentation (PASEF) with a Novel Trapped Ion Mobility Mass Spectrometer. *Mol. Cell Proteomics* **17**, 2534-2545 (2018).
