# Supplementary material for: Deciphering intercellular signaling complexes by interaction-guided chemical proteomics
Source: Nat Commun. 2023 Jul 12;14:4138. doi: 10.1038/s41467-023-39881-9 (PMC10338493; doi:10.1038/s41467-023-39881-9)

Replicate 1

PLAU treated hPSC cells

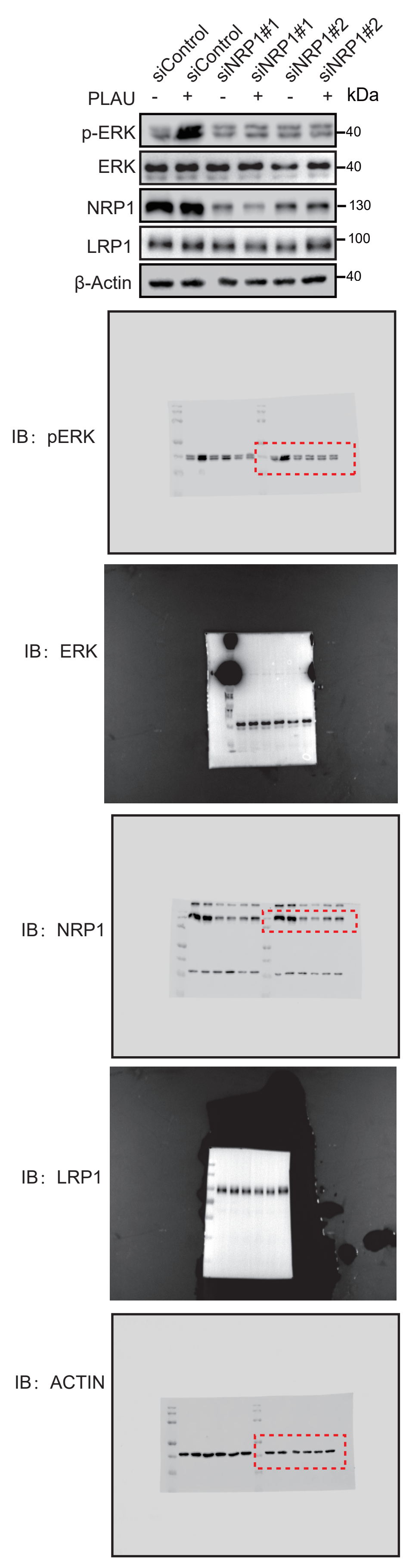

Replicate 2

PLAU treated hPSC cells

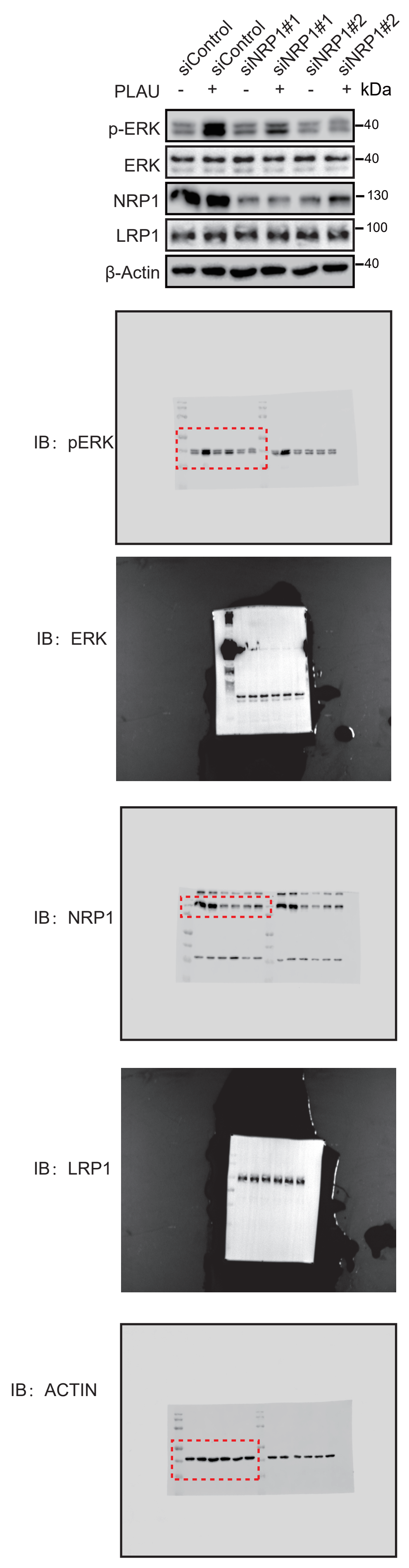

Replicate 3

PLAU treated hPSC cells

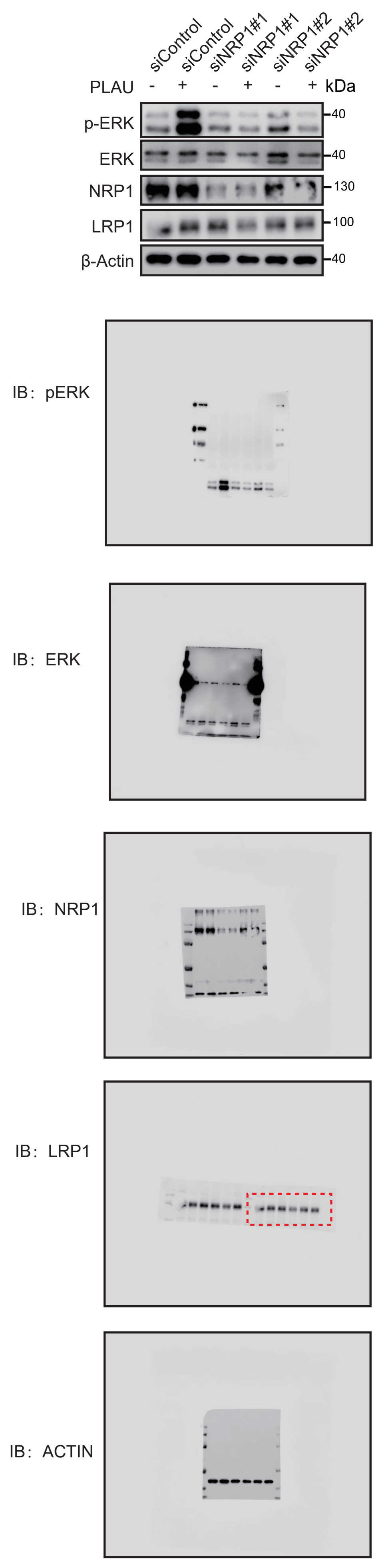

Supplement: Supplementary file 3 — Source Data [file 41467_2023_39881_MOESM3_ESM.zip › Source Data Fig. 6g.pdf]
