# Supplementary figures and images for: Deciphering intercellular signaling complexes by interaction-guided chemical proteomics
Source: Nat Commun. 2023 Jul 12;14:4138. doi: 10.1038/s41467-023-39881-9 (PMC10338493; doi:10.1038/s41467-023-39881-9)

Fig. 4h

Replicate 1

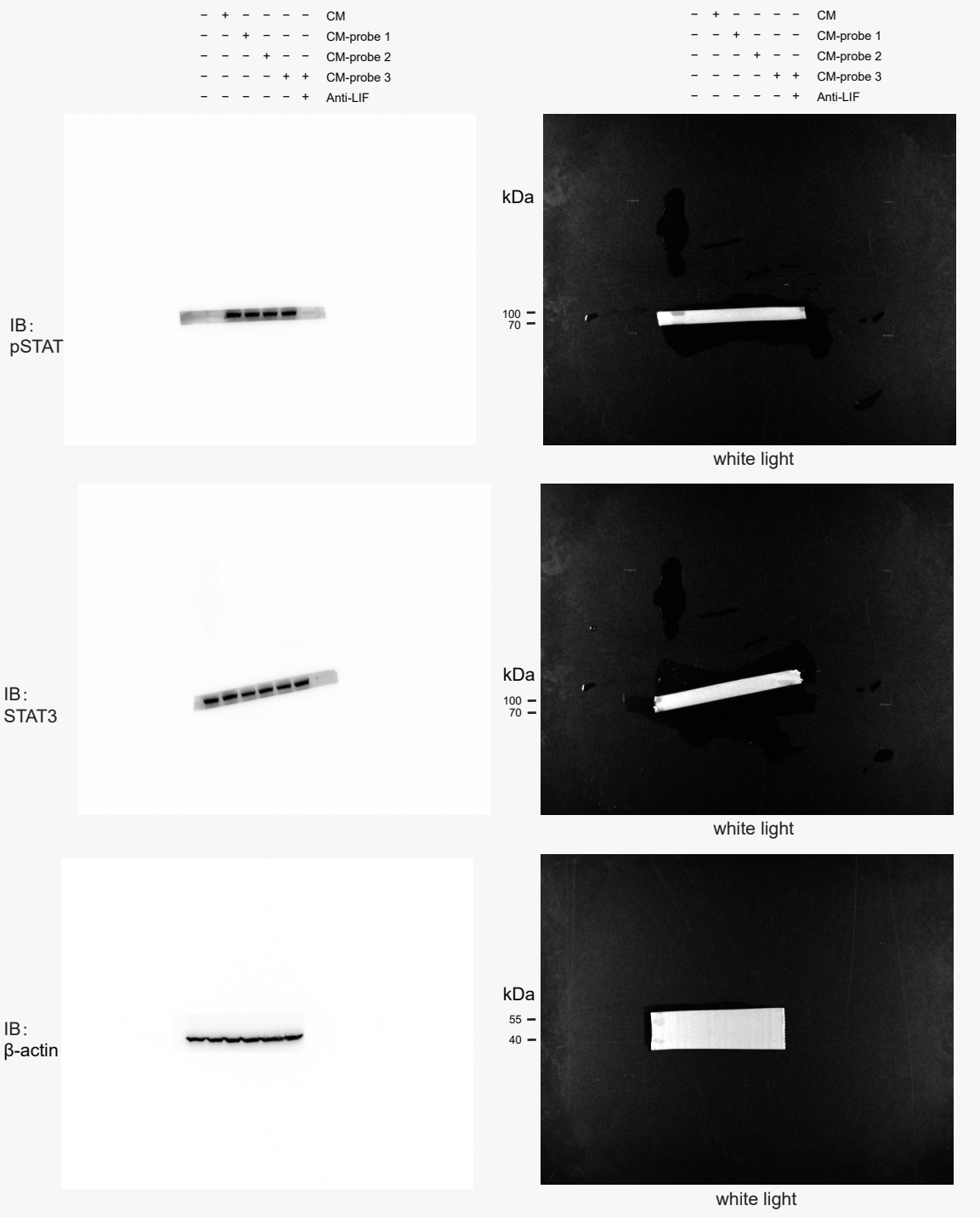

Replicate 2

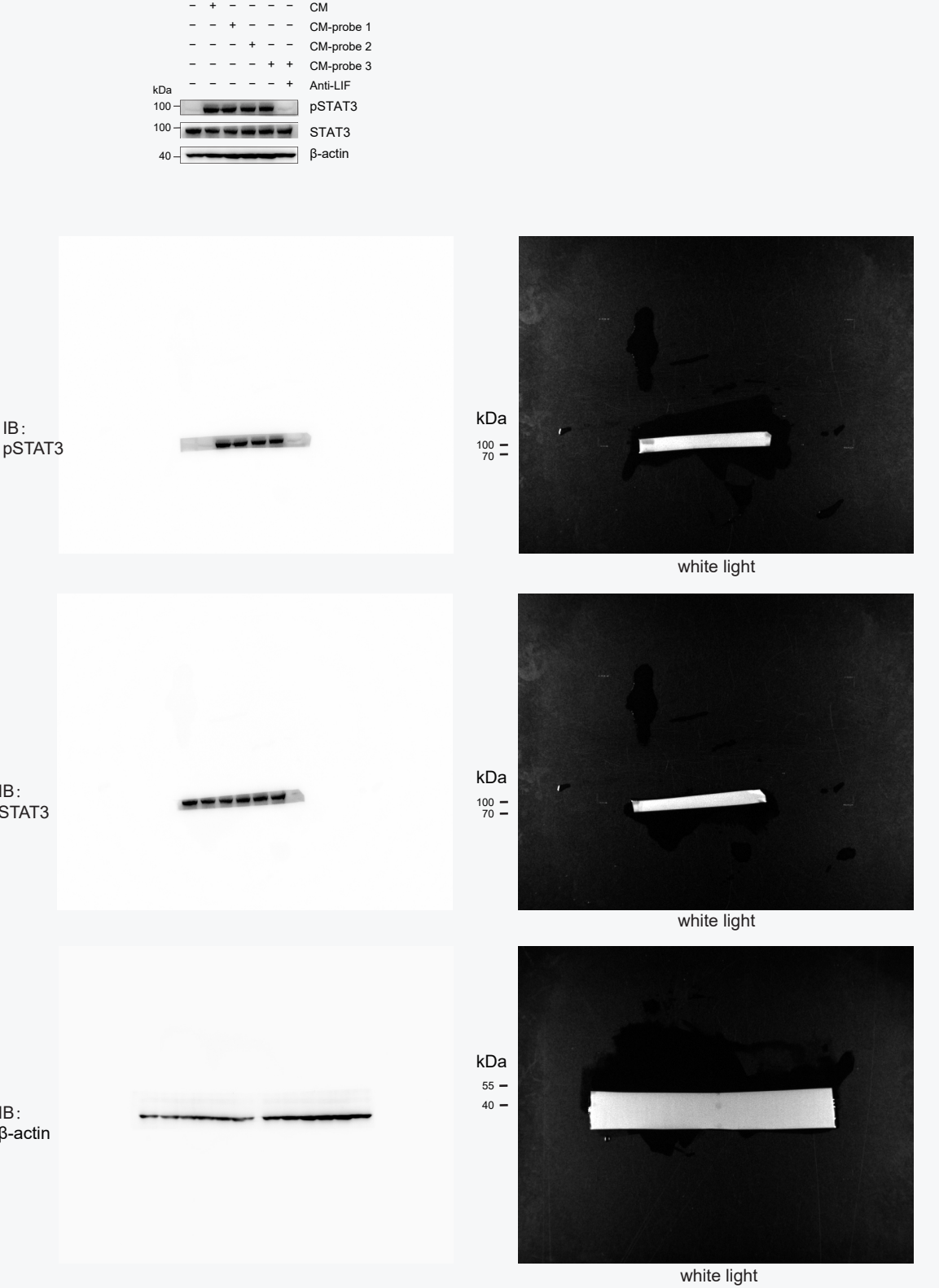

Replicate 3

Imaging on an Odyssey infrared scanner (LICOR Bioscience).

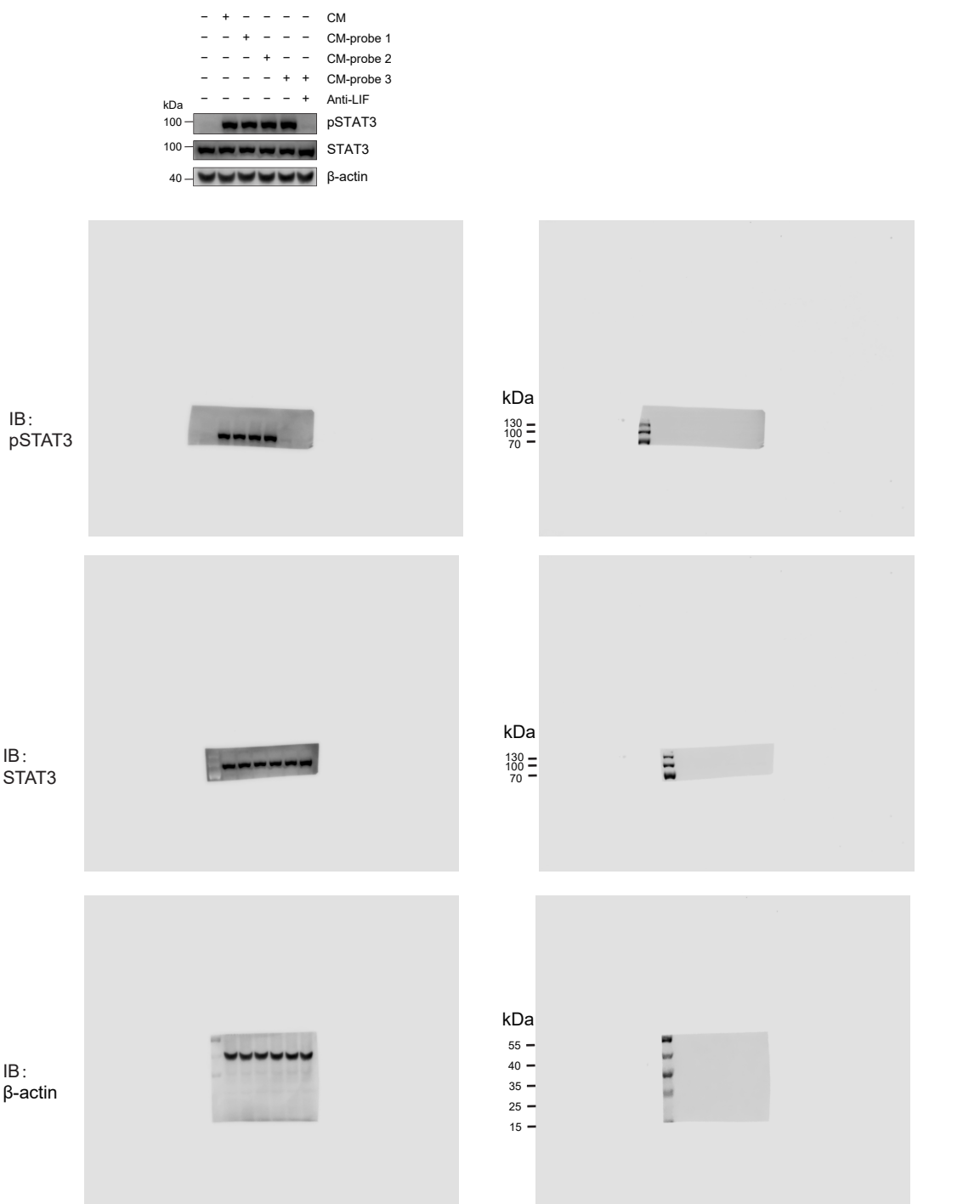

Supplement: Supplementary file 3 — Source Data [file 41467_2023_39881_MOESM3_ESM.zip › Source Data Fig. 4h.pdf]

Fig. 6b

Replicate 1

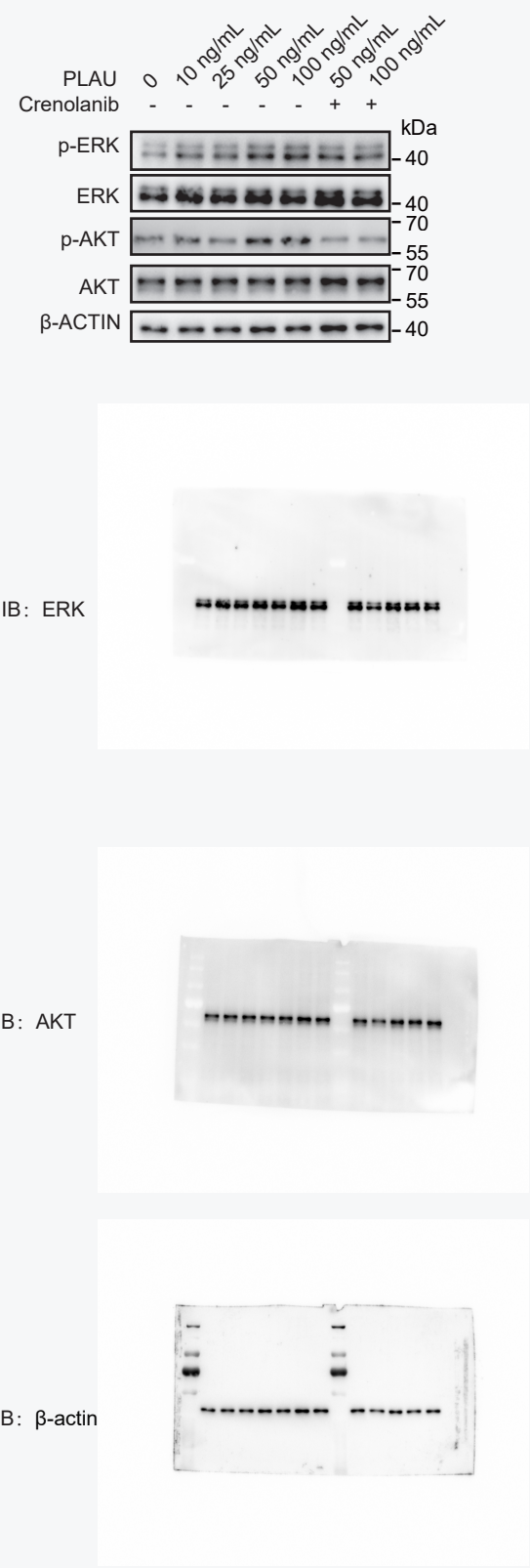

Replicate 2

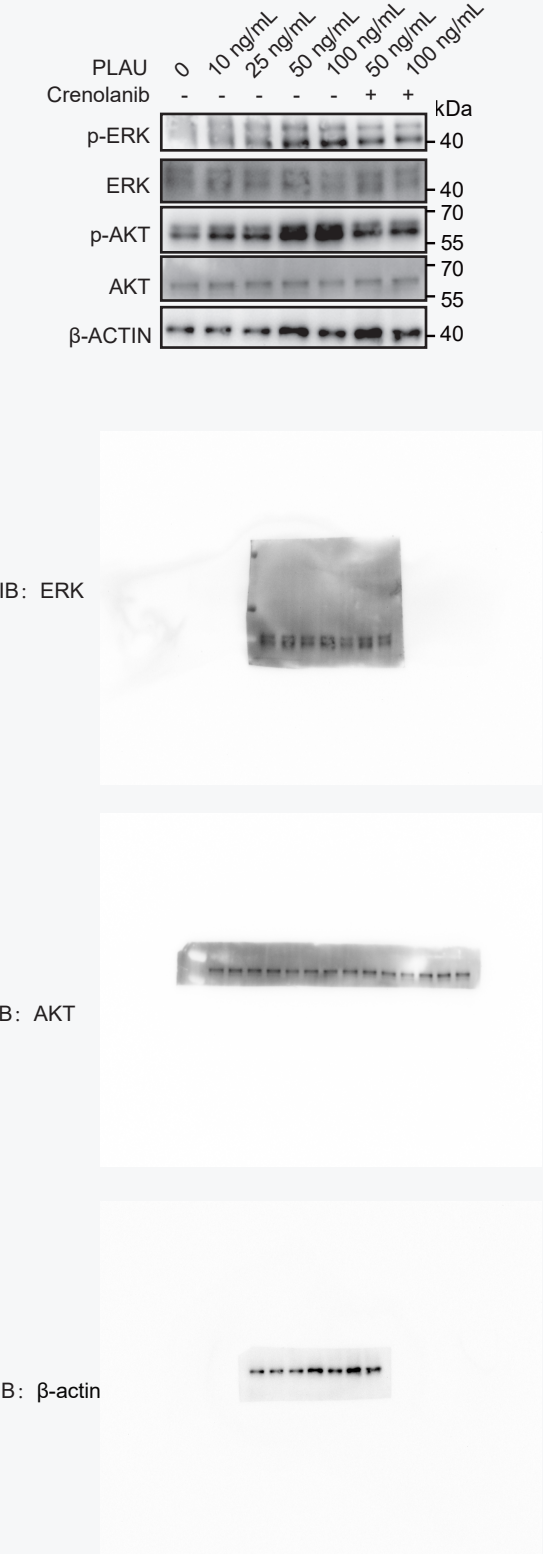

Replicate 3

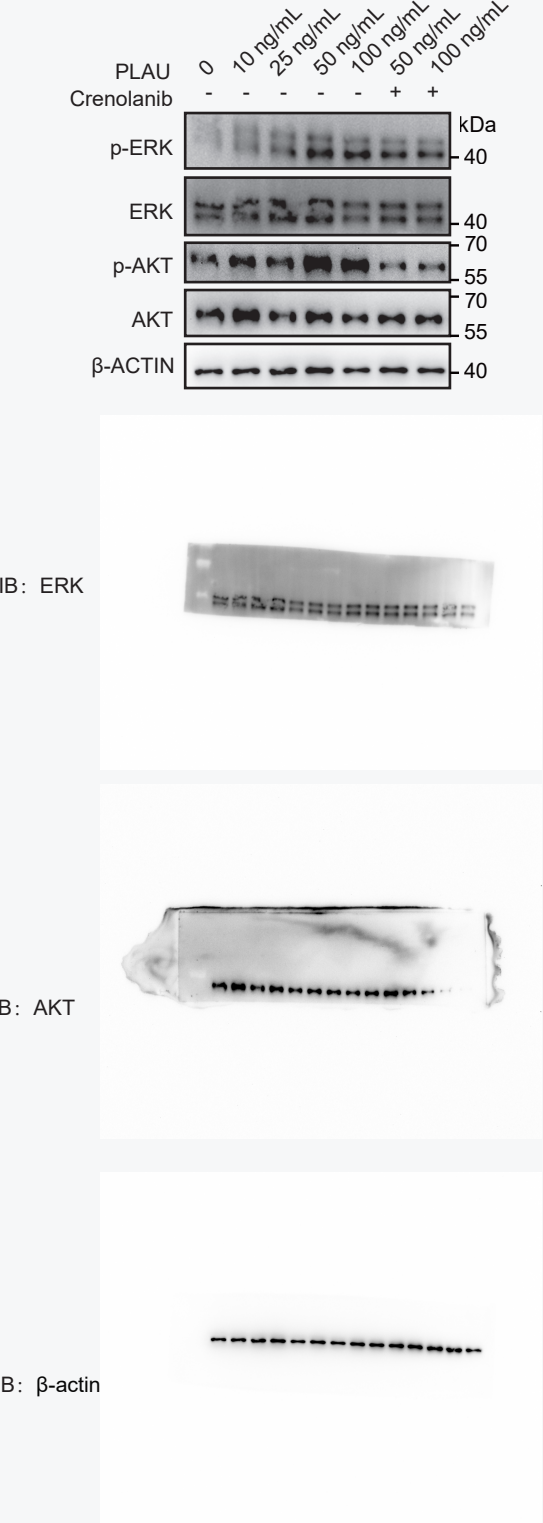

Supplement: Supplementary file 3 — Source Data [file 41467_2023_39881_MOESM3_ESM.zip › Source Data Fig. 6b.pdf]
